# Palladium-catalyzed carbonylative synthesis of acrylamides from alkenyl thianthrenium salts

**DOI:** 10.1039/d5sc06079f

**Published:** 2025-10-20

**Authors:** Ru-Han A, Jiajun Zhang, Xiao-Feng Wu

**Affiliations:** a Dalian National Laboratory for Clean Energy, Dalian Institute of Chemical Physics, Chinese Academy of Sciences 116023 Dalian Liaoning China xwu2020@dicp.ac.cn; b Leibniz-Institut für Katalyse e.V. Albert-Einstein-Straße 29a 18059 Rostock Germany Xiao-Feng.Wu@catalysis.de

## Abstract

Covalent drugs have emerged as powerful tools in modern therapeutics, with acrylamide being one of the most widely used electrophilic warheads in recent years. Herein, we report a practical and selective strategy for synthesizing acrylamides *via* Pd-catalyzed carbonylative amidation of vinyl thianthrenium salts with arylamines. These salts serve as stable and safe surrogates for alkynes or activated alkenes. The reaction proceeds under mild conditions using only 0.1 mol% Pd catalyst and 1 atm of CO, delivering a broad range of *N*-aryl acrylamides in high yields (>90%) with excellent chemoselectivity and functional group tolerance. Furthermore, the method is readily scalable to the gram level without compromising efficiency. This work demonstrates the synthetic potential of vinyl thianthrenium salts in carbonylation chemistry and offers a general, robust, and scalable approach for the preparation of functionalized acrylamides under mild conditions.

## Abstract 2

A practical strategy for synthesizing acrylamides *via* Pd-catalyzed carbonylative amidation of vinyl thianthrenium salts with arylamines has been developed.

## Graphical abstract

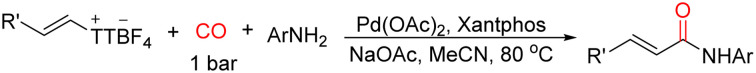

## Introduction

**Fig. 1:**
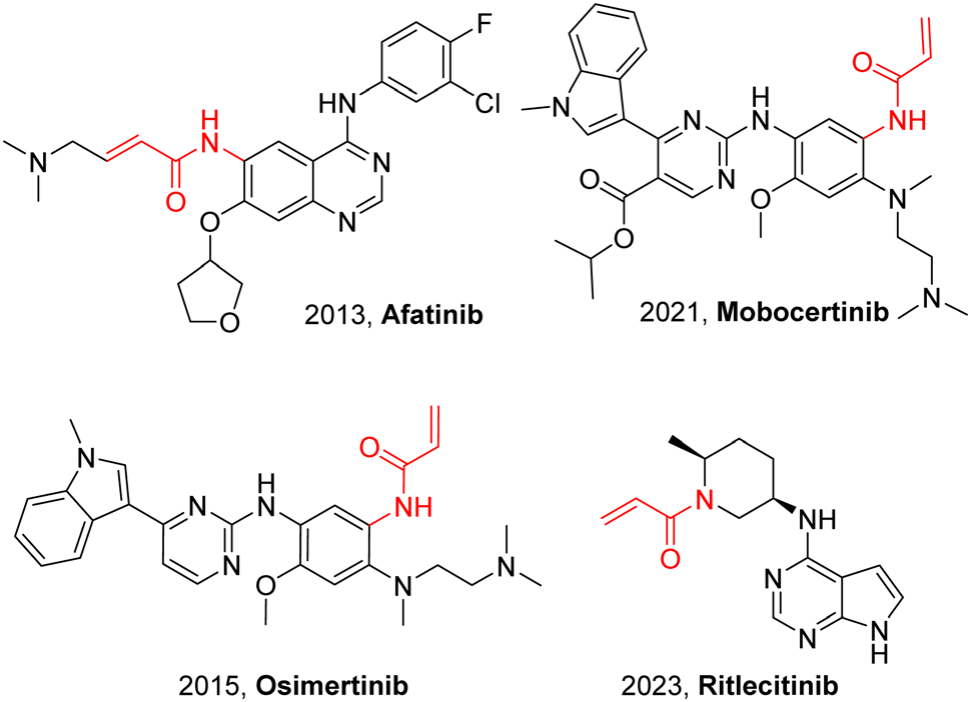
Selected covalent drugs containing acrylamide warheads.

Acrylamide has emerged as a versatile and valuable structural motif in both medicinal chemistry and organic synthesis due to its broad reactivity and ability to form covalent bonds with biomolecular targets.^1–4^ In particular, its acrylamide moiety serves as an efficient electrophilic warhead, undergoing selective Michael addition with nucleophilic residues in proteins.^5,6^ Over the past decades, covalent inhibitors bearing acrylamide motifs have emerged as a central strategy in drug discovery (Fig. 1).^7–10^ Beyond pharmaceuticals, acrylamide is a key intermediate in organic synthesis, participating in various transformations and acrylamide monomers are also the fundamental building blocks of polyacrylamides, which are widely used in water treatment, enhanced oil recovery, and biomedical materials.^11–16^ Thus, the development of efficient synthetic methods for acrylamide derivatives is of significant research and practical value.

Traditionally, acrylamides are prepared by nucleophilic condensation or substitution of amines with carboxylic acid derivatives in the presence of stoichiometric bases or coupling reagents;^17–21^ however, such protocols are often non-green, atom-inefficient, and occasionally suffer from chemoselectivity issues. The hydration of acrylonitrile remains the dominant industrial route,^22–25^ especially when combined with nitrile hydratase (NHase),^25–29^ which enables highly efficient production under mild conditions. In recent years, new methods for synthesizing acrylamide were reported,^30–34^ though they remain largely at the research stage with limited industrial implementation.

**Scheme 1:**
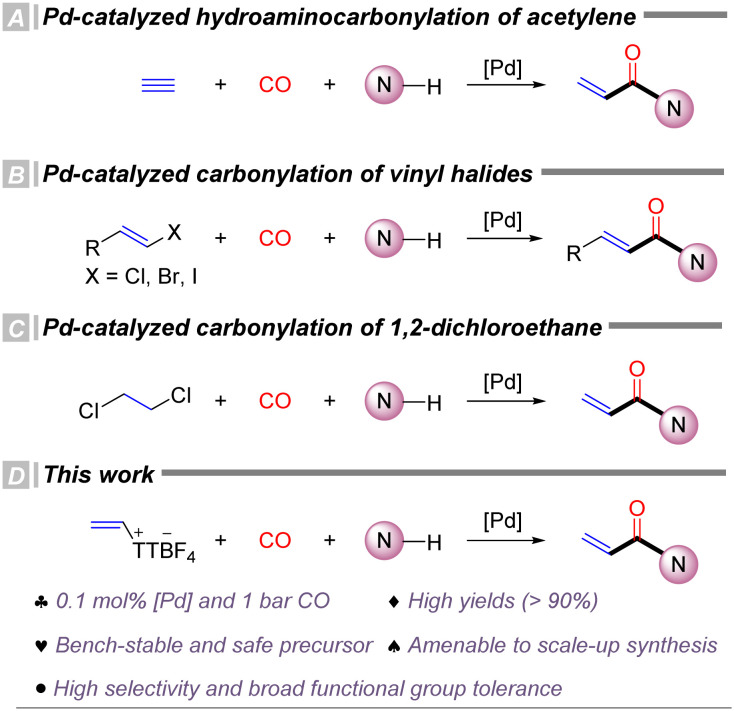
Studies on carbonylation for acrylamide synthesis.

Carbon monoxide (CO) is one of the most atom-economical, inexpensive, and efficient C1 sources for constructing carbonyl-containing functionalities,^35,36^ including esters, amides, carboxylic acids, ketones, and acyl halides.^37–40^ Over the past several decades, CO has been extensively utilized in transition metal catalyzed carbonylation reactions, numerous studies have reported the synthesis of acrylamides *via* transition metal catalyzed carbonylation. Despite these advances, carbonylation reactions involving the simplest alkyne, acetylene, and *N*-nucleophiles remain relatively scarce. In 1953, Reppe *et al.* first reported the catalytic hydroamino–carbonylation of acetylene to yield acrylamides (Scheme 1, eqn (A)).^41^ However, this reaction was severely limited by the need for high temperatures (180 °C), high acetylene pressures (>12 bar), and highly toxic Ni(CO)_4_ catalysts. In 2024, Beller's group reported the first palladium-catalyzed hydroaminocarbonylation of acetylene, achieving moderate to excellent yields under milder conditions (Scheme 1, eqn (B)).^42^ Acrylamides can also be synthesized from activated alkenes,^43,44^ such as vinyl chloride or vinyl iodide, through carbonylation, thereby circumventing the use of highly hazardous acetylene gas and improving operational safety and scalability. In 2024, our group reported the first palladium-catalyzed carbonylation using 1,2-dichloroethane as the starting material,^45^ efficiently producing a range of acrylamides and acrylate ester derivatives, further avoiding the use of hazardous acetylene and vinyl chloride (Scheme 1, eqn (C)). Nonetheless, these methodologies still exhibit certain limitations concerning catalyst loading, operational safety, reaction selectivity and polymerization of the substrate. Therefore, there remains a strong demand for the development of greener, more efficient, and readily scalable synthetic strategies. On other hand, carbonylative transformation of thianthrenium salts has been recognized as an interesting topic and various procedures have been achieved.^46^ In our group, we also achieved the carbonylative reactions of aryl thianthrenium salts with arylboronic acids^47^ and terminal alkynes^48^ as the reaction partners. By using palladium as the catalyst, diaryl ketones, alkynones and furanones can be produced effectively. However, the carbonylative transformation with alkenyl thianthrenium salts has not been reported yet which can give different analogue of carbonyl-containing products. Under the above backgrounds, in this study, we developed a palladium-catalyzed carbonylative amidation using alkenyl thianthrenium salts as safer and more stable alternatives to alkynes or activated alkenes, allowing the synthesis of acrylamide derivatives from arylamines under mild conditions (Scheme 1, eqn (D)).

## Results and discussion

We began our investigations by employing vinyl thianthrenium salt 1a as a surrogate for alkynes, using aniline as the nucleophile to construct the corresponding acrylamide. A systematic screening was conducted with respect to the catalyst and its loading, ligands, bases, solvents, and CO pressure to identify optimal conditions for the transformation (for details see, SI). These studies ultimately led to the establishment of an efficient catalytic system, which laid a solid foundation for subsequent substrate scope exploration. Initially, catalyst and ligand screenings revealed that palladium was essential for the reaction, as demonstrated by the complete failure of the transformation in its absence. Among various combinations, Pd(OAc)_2_ and xantphos proved to be the most effective, delivering the highest yield of 3aa. Solvents screening showed that MeCN was the most suitable, offering good solubility for both the palladium complex and organic substrates. In contrast, aromatic solvents (Table 1, entry 5) and highly polar solvents (Table 1, entry 9) gave inferior results. Interestingly, the reaction tolerated the presence of water (Table 1, entries 5, 6, and 8), and in some cases, water may even improve the reaction by enhancing base solubility.

**Table 1:** Optimization of the reaction conditions^a^

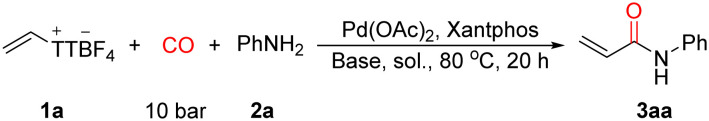					
Entry	[Pd] (mol%)	Ligand (mol%)	Base	Solvent	Yield^b^ (%)
1	4.0	6.0	Na_2_CO_3_	1,4-Dioxane	86
2^c^	4.0	0	Na_2_CO_3_	1,4-Dioxane	84
3	4.0	6.0	NaOAc	1,4-Dioxane	95
4	1.0	1.2	NaOAc	MeCN	95^d^
5	1.0	1.2	NaOAc	Tol.	36
6	1.0	1.2	NaOAc	Tol. + H_2_O	69
7	1.0	1.2	NaOAc	DMF	13
8	1.0	1.2	NaOAc	H_2_O	35
9	1.0	1.2	NaOAc	DMSO	n.d.
10	1.0	1.2	NaOH	MeCN	19
11	1.0	1.2	Li_2_CO_3_	MeCN	26
12	1.0	1.2	Cs_2_CO_3_	MeCN	81
13	1.0	1.2	—	MeCN	65
14	1.0	1.2	TEA	MeCN	14
15^e^	1.0	1.2	NaOAc	MeCN	97
16^e^	0.1	0.12	NaOAc	MeCN	99
17^e^	0.01	0.012	NaOAc	MeCN	42
18^e^	—	0.12	NaOAc	MeCN	n.d.
19^e^^,^^f^	0.1	0.12	NaOAc	MeCN	77
20^e^^,^^g^	0.1	0.12	NaOAc	MeCN	72

a Reaction conditions: 1a (0.1 mmol), 2a (1.2 equiv.), base (2.0 equiv.), solvent (1.0 mL), CO (10 bar), 20 h.

b The yields were determined by GC using *n*-hexadecane as the internal standard.

c Pd(PPh_3_)_4_.

d Isolated yield.

e CO (1 bar).

f Reaction time 10 h.

g At room temperature.

In terms of base selection, the reaction was found to be relatively base-tolerant, achieving 65% yield even under base-free conditions, suggesting that while a base is not strictly necessary, it can enhance efficiency. Among various bases evaluated, sodium acetate (NaOAc) emerged as the optimal choice, likely due to its balanced basicity and coordination ability, which may promote the Pd(ii)/Pd(0) cycle or stabilize key intermediates. Strong bases (Table 1, entry 10) or organic bases (Table 1, entry 14) were detrimental to the reaction. We also evaluated the influence of CO pressure, reaction time and reaction temperature (Table 1, entries 15–20). While the initial optimization was performed under 10 bar of CO, we were pleased to find that the yield remained high even under 1 bar CO, significantly improving the operational safety and scalability of the protocol (Table 1, entry 15). Notably, the catalyst loading could be reduced from 4.0 mol% to as low as 0.1 mol% without compromising the yield, which even reached 99%, underscoring the high efficiency of the catalytic system (Table 1, entry 16). However, when the Pd loading was further decreased to 0.01 mol%, the yield dropped significantly, and prolonging the reaction time failed to improve the outcome (Table 1, entry 17), and no desired produced detectable in the absence of palladium (Table 1, entry 18). This may be attributed to a slower reaction rate under ultra-low Pd concentrations, leading to premature decomposition of the vinyl-TT salt before productive turnover. Moreover, the reaction maintained over 70% yield when either the temperature was lowered to room temperature or the reaction time was halved, further highlighting the high catalytic activity and practical potential of the system (Table 1, entries 19 and 20). In addition, Pd(PPh_3_)_4_ or Pd_2_dba_3_ can be applied as pre-catalyst as well and similar results can be obtained in the presence of xantphos. The yield of the desired product was formed in 81% in the presence of 1 equivalent of base. Decreased yields were obtained with shorten reaction time or decreased reaction temperature. Taken together, the optimized conditions were established as follows: Pd(OAc)_2_ (0.1 mol%), xantphos (0.12 mol%), NaOAc (2.0 equiv.), MeCN (1.0 mL) as solvent, 1 bar CO, at 80 °C for 20 h.

**Scheme 2:**
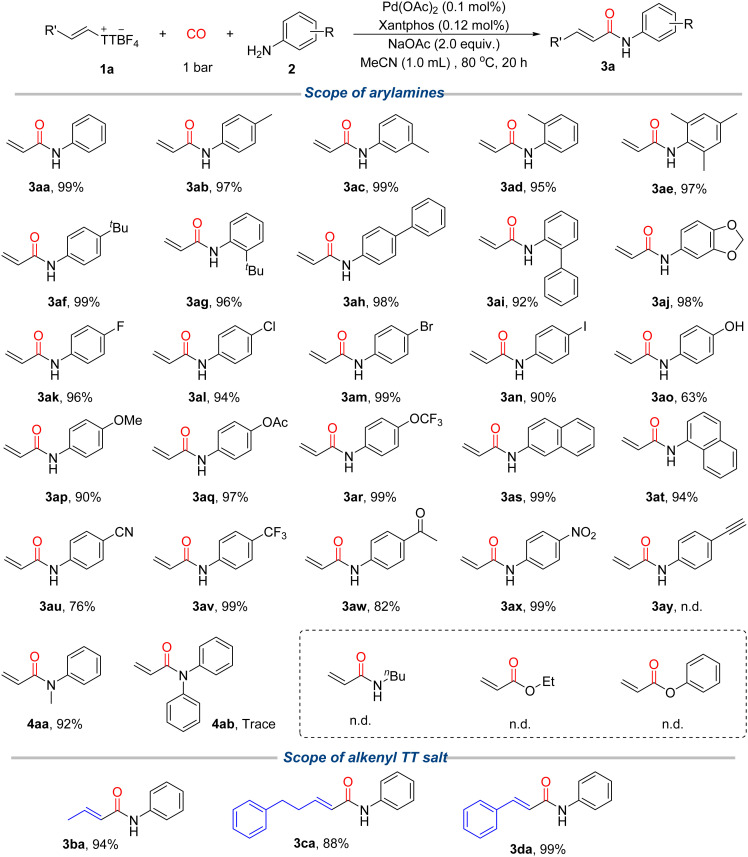
Substate scope of arylamines and alkenyl thianthrenium salts. Reaction conditions: 1a (0.1 mmol), 2a (1.2 equiv.), NaOAc (2.0 equiv.), MeCN (1.0 mL), CO (1 bar), 80 °C, 20 h, isolated yield.

With the optimized conditions in hand, we next explored the substrate scope of this palladium catalyzed carbonylative amidation, focusing on both amines and alkenyl thianthrenium salts (Scheme 2). A wide range of arylamines proved compatible with this transformation, delivering the corresponding *N*-aryl acrylamides in generally excellent yields. Electron-donating substituents such as methyl, *tert*-butyl, methoxy, and acetoxy at the *para*- or *meta*-positions were well tolerated, affording products 3aa–3af and 3ap–3aq in 90–99% yields. These substituents typically enhance the nucleophilicity of anilines, facilitating the amidation and indicating that the reaction proceeds efficiently even with moderately nucleophilic amines. Moreover, substitution at different positions on the aryl ring, including *para*-, *meta*-, and even a sterically hindered group at *ortho*, did not significantly affect the yield, demonstrating the excellent steric tolerance of the catalytic system. Interestingly, when both amino and hydroxyl groups are present on the aromatic ring, the reaction proceeds selectively at the amino group to afford product 3ao. Electron-deficient arylamines bearing halogens, cyano, trifluoromethyl, or nitro groups also participated smoothly in the coupling, giving products 3ak–3an and 3au–3ax in 76–99% yields. Notably, strongly electron-withdrawing groups such as NO_2_ and CF_3_ were well tolerated without compromising yield, reflecting the high functional group compatibility and robustness of the catalytic system. The excellent chemoselectivity is further demonstrated by the fact that even in the presence of highly reactive iodide substituents, the reaction proceeded exclusively at the amino moiety with high efficiency, providing opportunities for further late-stage derivatization and cross-coupling. Heterocyclic arylamines such as 2-aminobenzofuran and fused aromatic systems like 2-naphthylamine also reacted smoothly, affording the desired products in over 90% yield, highlighting the method's potential utility for the construction of bioactive heterocyclic motifs. In contrast, when an alkynyl-substituted arylamine was employed, the desired product 3ay was not observed. Instead, the hydrolysis product 3aw was obtained in large quantities, indicating that 3ay is highly sensitive to trace amounts of water under the reaction conditions and readily undergoes hydrolysis. In addition to primary arylamines, we also evaluated the reactivity of tertiary amines. For instance, *N*-methylaniline delivered the corresponding product 4aa in excellent yield, whereas bulky tertiary amines such as diphenylamine failed to give any product (4ab), likely due to steric hindrance. Furthermore, aliphatic amines, alcohols, and phenols were not viable nucleophiles under these conditions, indicating that the protocol is highly selective for arylamines. In the case of aliphatic amine and benzyl amine, only the corresponding formamide compounds were detected. In the cases of 2-aminopyridine and 4-aminopyridine, the desired product was detected on trace amount. We further examined the generality of this reaction with respect to the alkenyl thianthrenium salt component. A series of alkenes bearing either aryl or alkyl side chains were successfully converted *via* their corresponding TT salts, giving products 3ba–3da in 88–99% yields. These results underscore the versatility of alkenyl thianthrenium salts as robust electrophilic surrogates in carbonylation chemistry.

**Fig. 2:**
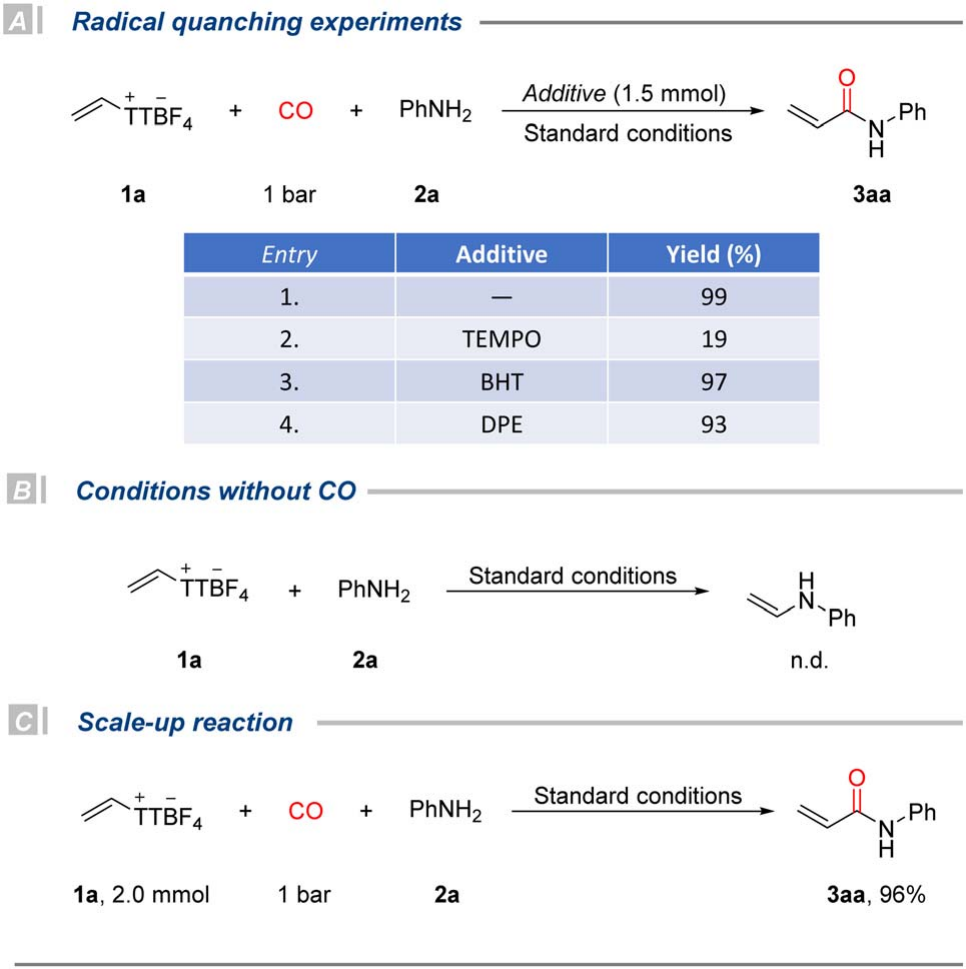
Control experiments and scale-up reaction.

To gain some insights into the reaction mechanism, radical quenching experiments were conducted under standard conditions (Fig. 2A). The addition of TEMPO (2,2,6,6-tetramethylpiperidine-1-oxyl) significantly suppressed the formation of the desired amide product 3aa, resulting in only 19% yield. This result suggests the possible involvement of a radical intermediate, but the oxidation property of TEMPO should also be considered as it might oxidize phosphine or the *in situ* generated Pd(0) intermediate. The reaction proceeded smoothly, when BHT (2,6-di-*tert*-butyl-4-methylphenol) or DPE (1,1-diphenylethylene) was added, furnishing 3aa in 97% and 93% yields, respectively. These results imply that the process likely proceeds through a non-radical mechanism. In addition, a control experiment conducted in the absence of CO resulted in no product formation (Fig. 2B), highlighting the essential role of carbon monoxide in the reaction. To evaluate the practicality and scalability of the developed method, we performed a gram–scale reaction using 2.0 mmol of vinyl thianthrenium salt 1a (Fig. 2C). The reaction proceeded efficiently under the optimized conditions, affording the desired product 3aa in 96% isolated yield, demonstrating the excellent robustness and scalability of the protocol for potential synthetic applications.

**Scheme 3:**
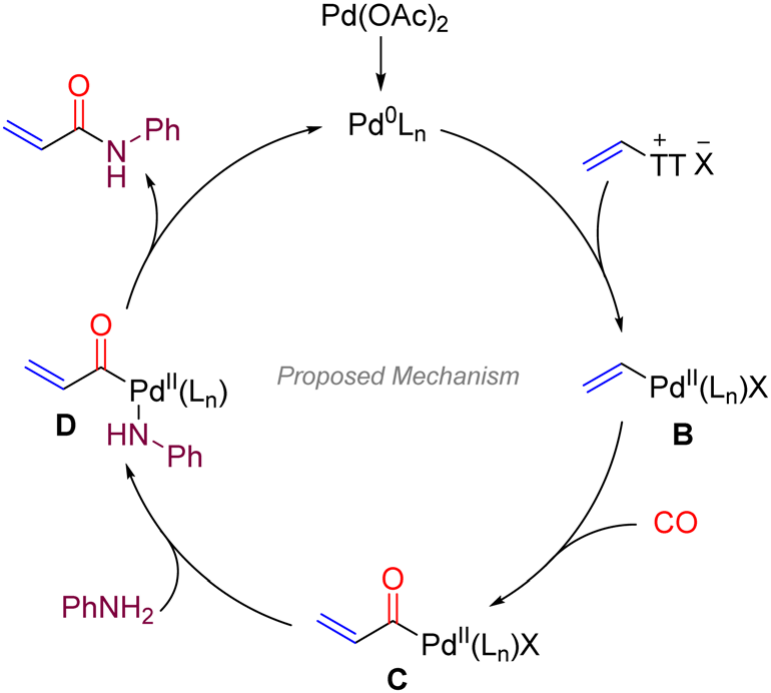
Proposed mechanism.

Based on our results and understanding, a plausible catalytic cycle for this carbonylative amidation is outlined in Scheme 3. The reaction is proposed to be initiated by oxidative addition of Pd(0) into the C–S bond of the vinyl thianthrenium salt, forming a vinyl-Pd(ii) complex (B). The coordinating of CO with vinyl-Pd(ii) complex (B) supposed to be important for its reactivity and stability which also explains no product was detected in the absence of CO in control experiment. Then, this intermediate undergoes migratory insertion of carbon monoxide to generate an acyl-Pd(ii) species (C). Coordination and nucleophilic attack by the arylamine then affords intermediate D, which undergoes reductive elimination to deliver the final acrylamide product and regenerate the catalytically active Pd(0) species. This pathway is consistent with the requirement of CO for product formation and supports the critical role of TT^+^ as a leaving group in initiating the Pd-catalyzed cycle.

## Conclusions

In summary, we have developed an efficient and highly selective Pd-catalyzed carbonylative amidation of vinyl thianthrenium salts with arylamines under mild conditions, requiring only 0.1 mol% of catalyst and 1 bar of CO. This strategy enables the synthesis of a broad range of *N*-aryl acrylamide derivatives in excellent yields, typically exceeding 90%, and shows broad functional group compatibility. Importantly, vinyl thianthrenium salts serve as effective electrophilic surrogates for alkynes or activated alkenes, enabling carbonylative amidation under mild conditions and without the need for alkyne substrates. The method also demonstrates excellent scalability, affording the desired product in 96% yield on gram scale. This work highlights the synthetic potential of vinyl thianthrenium salts in carbonylation chemistry, and offers a practical and general approach for the synthesis of functionalized acrylamides under mild and scalable conditions.

## Author contributions

R. H. A. performed all the experiments and prepared the manuscript and SI. J. Z. prepared some substrates. X. F. W. conceived the project, supervised the research, and revised the manuscript.

## Conflicts of interest

There are no conflicts to declare.

## Supplementary Material

SC-016-D5SC06079F-s001

## Data Availability

The data supporting this article have been included as part of the supplementary information (SI). Supplementary information: general comments, general procedure, analytic data, and NMR spectra. See DOI: https://doi.org/10.1039/d5sc06079f.

## References

[cit1] Singh J., Petter R. C., Baillie T. A., Whitty A. (2011). Nat. Rev. Drug Discovery.

[cit2] Cohen P., Cross D., Jänne P. A. (2021). Nat. Rev. Drug Discovery.

[cit3] Boike L., Henning N. J., Nomura D. K. (2022). Nat. Rev. Drug Discovery.

[cit4] Schaefer D., Cheng X. (2023). Pharmaceuticals.

[cit5] Zheng L., Li Y., Wu D., Xiao H., Zheng S., Wang G., Sun Q. (2023). MedComm: Oncol..

[cit6] Lee C. S., Milone M., Seetharamu N. (2021). OncoTargets Ther..

[cit7] Ran F., Liu Y., Wang C., Xu Z., Zhang Y., Liu Y., Zhao G., Ling Y. (2022). Eur. J. Med. Chem..

[cit8] Duan X., Zheng N., Li M., Liu G., Sun X., Wu Q., Song W. (2022). Nat. Commun..

[cit9] Schaefer D., Cheng X. (2023). Pharmaceuticals.

[cit10] Syed Y. Y. (2020). Drugs.

[cit11] Huo Y. W., Yao L., Qi X., Wu X. F. (2021). Org. Chem. Front..

[cit12] Blair H. A. (2023). Drugs.

[cit13] Gou S., He Y., Ma Y., Luo S., Zhang Q., Jing D., Guo Q. (2015). RSC Adv..

[cit14] de Cássia Novaes W., Berg A. (2003). Aesthetic Plast. Surg..

[cit15] Liang S., Liu Y., Hu S., Shen A., Yu Q., Yan H., Bai M. (2019). Energies.

[cit16] Fouda A. S., Khalil E. M., El-Mahdy G. A., Shaban M. M., Mohammed A. S., Abdelsatar N. A. (2023). Sci. Rep..

[cit17] Wang X. (2006). J. Chem. Res..

[cit18] Luo Q. L., Lv L., Li Y., Tan J. P., Nan W., Hui Q. (2011). Eur. J. Org Chem..

[cit19] Abe Y., Emori K. (2022). Org. Process Res. Dev..

[cit20] Zhang L., Bai S., Zheng L. (2023). Organometallics.

[cit21] Hossain M., Habib I., Singha K., Kumar A. (2024). Heliyon.

[cit22] Ahmed T. J., Knapp S. M. M., Tyler D. R. (2011). Coord. Chem. Rev..

[cit23] Ichikawa S., Miyazoe S., Matsuoka O. (2011). Chem. Lett..

[cit24] Battilocchio C., Hawkins J. M., Ley S. V. (2014). Org. Lett..

[cit25] Cirri D., Marzo T., Pratesi A. (2023). Front. Chem..

[cit26] Yamada H., Kobayashi M. (1996). Biosci. Biotechnol. Biochem..

[cit27] Martinez S., Wu R., Sanishvili R., Liu D., Holz R. (2014). J. Am. Chem. Soc..

[cit28] Cheng Z., Xia Y., Zhou Z. (2020). Front. Bioeng. Biotechnol..

[cit29] Ma D., Cheng Z., Peplowski L. (2022). et. al.. Chem. Sci..

[cit30] Fouda F., Khalil E., El-Mahdy G. (2023). et al.. Sci. Rep..

[cit31] Luo Q. L., Lv L., Li Y., Tan J. P., Nan W., Hui Q. (2011). Eur. J. Org Chem..

[cit32] Quan Z. J., Xia H. D., Zhang Z., Da Y. X., Wang X. C. (2014). Appl. Organomet. Chem..

[cit33] Pandolfi F., Chiarotto I., Mattiello L., Petrucci R., Feroci M. (2019). ChemistrySelect.

[cit34] Yang K., Li Y., Ma Z., Tang L., Yin Y., Zhang H., Li Z., Sun X. (2019). Eur. J. Org Chem..

[cit35] Wang L. C., Chen B., Wu X. F. (2022). Angew. Chem., Int. Ed..

[cit36] Wang Y., Yang H., Zheng Y. (2024). et al.. Nat. Catal..

[cit37] Wang L. C., Chen B., Zhang Y., Wu X. F. (2022). Angew. Chem., Int. Ed..

[cit38] Xu R. R., Wen D., Qi X., Wu X. F. (2022). Org. Biomol. Chem..

[cit39] Wu F. P., Yang Y., Fuentes D. P., Wu X. F. (2022). Chem.

[cit40] A R. H., Bao Z. P., Huo Y. W., Wu X. F. (2024). Chem.–Asian J..

[cit41] Reppe W. (1953). Adv. Cycloaddit..

[cit42] Cao Z., Wang Q., Neumann H., Beller M. (2024). Angew. Chem., Int. Ed..

[cit43] Eriksson J., Åberg O., Långström B. (2007). Eur. J. Org Chem..

[cit44] Li Y., Jing Y., Shi Y. R., Li H., Yang M. G., Kou Y. L., Fan Q. W. (2024). Eur. J. Org Chem..

[cit45] Xu R. R., Kuai C. S., Wu X. F. (2024). Catal. Sci. Technol..

[cit46] Zhao Y.-H., Wu X.-F. (2025). Chem.: Methods.

[cit47] Zhang J., Wu X.-F. (2023). Org. Lett..

[cit48] Zhao Y.-H., Gu X.-W., Wu X.-F. (2024). Org. Lett..

