# Supplementary material for: Palladium-catalyzed carbonylative synthesis of acrylamides from alkenyl thianthrenium salts
Source: Chem Sci. 2025 Oct 20;16(46):22168–73. doi: 10.1039/d5sc06079f (PMC12548753; doi:10.1039/d5sc06079f)
Supplement: SC-016-D5SC06079F-s001 [file SC-016-D5SC06079F-s001.pdf]

Supplementary Information (SI) for Chemical Science.
This journal is © The Royal Society of Chemistry 2025

Supporting Information

Palladium-Catalyzed Carbonylative Synthesis of Acrylamides from Alkenyl Thianthrenium Salts

Ru-Han A[a,b], Jiajun Zhang[a,b], Xiao-Feng Wu[a,b,*]

[a]*Dalian National Laboratory for Clean Energy, Dalian Institute of Chemical Physics, Chinese Academy of Sciences, Dalian 116023, Liaoning, China*

[b]*Leibniz-Institut für Katalyse e. V., Albert-Einstein-Straße 29a, 18059 Rostock, Germany*

Email: xwu2020@dicp.ac.cn (X.-F. Wu)

Table of Contents

1. General information 2
2. Optimization of reaction conditions 3
3. General Procedures for Synthesis of TT salt 5
   3.1 Synthesis of thianthrenium salts **1a**, **1b**. 5
   3.2 Synthesis of thianthrenium salt **1c**. 5
   3.3 Synthesis of thianthrenium salts **1d**. 6
4. General Procedures 7
   4.1 General Procedures for 7
   4.2 Procedure for 2.0 mmol Scale Reaction 7
5. Characterization Data 8
6. Mechanism study experiment 15
   6.1 Radical quenching experiment 15
   6.2 Conditions without CO. 15
7. References 16
8. Copies of NMR Spectra 17

1. General information

Unless otherwise noted, all reactions were carried out under monoxide (CO) or nitrogen (N_2) atmosphere. All reagents were purchased from commercial suppliers and used without further purification. Column chromatography was performed on silica gel (200-300 meshes) using petroleum ether (bp. 60~90 °C), ethyl acetate, dichloromethane as eluent. All NMR spectra were recorded at ambient temperature using Bruker Avance III 700 MHz NMR spectrometers and spectral data were reported in ppm relative to tetramethylsilane (TMS) as the internal standard and DMSO-d_6 as solvent. All coupling constants (J) were reported in Hertz unite (Hz) with the following abbreviations: s = singlet, d = doublet, dd = double doublet, t = triplet, dt = double triplet, q = quatriplet, m = multiplet, br = broad. All reactions were monitored by GC-FID or NMR analysis, GC-yields were calculated using *n*-hexadecane as internal standard. All measurements were carried out at room temperature unless otherwise stated.

Because of the high toxicity of carbon monoxide, all the reactions should be performed in an autoclave. The laboratory should be well-equipped with a CO detector and alarm system.

2. Optimization of reaction conditions

Table S1. Effect of catalyst and ligand

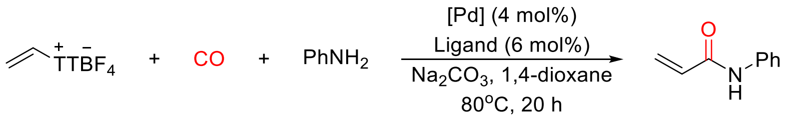

1a 10 bar 2a 3aa

Entry	Cat.	Ligand	Yield%[a]
1	$Pd(OAc)_2$	PPh_3	71
2	$Pd(OAc)_2$	DPPP	78
3	$Pd(OAc)_2$	BINAP	84
4	$Pd(OAc)_2$	DPPF	88
5	$Pd(OAc)_2$	DPEPhos	84
6	$Pd(OAc)_2$	Xantphos	86
7	$Pd(PPh_3)_4$	—	84
8[b]	$Pd(OAc)_2$	Xantphos	95[c]

Reaction conditions: **1a** (0.1 mmol, 1.0 equiv.), **2a** (0.12 mmol, 1.2 equiv.), $Pd(OAc)_2$ (4.0 mol%), Xantphos (6.0 mol%), NaOAc (0.2 mmol, 2.0 equiv.), solvent (1.0 mL), CO (10 bar), 20 h. [a] The yields were determined by GC using *n*-hexadecane as the internal standard. [b] base : NaOAc. [c] isolated yield.

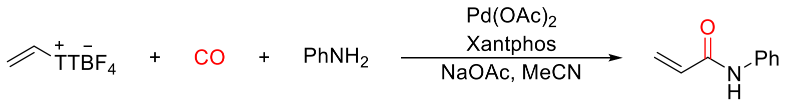

1a 2a 3aa

Entry	[Pd]/mol%	Xantphos/mol%	CO (bar)	Time (h)	Temp. (°C)	Yield%[a]
1	4	6	10	20	80	96
2	1	1.2	10	20	80	97
3	1	1.2	1	20	80	97
4	0.1	0.12	1	20	80	99
5	0.01	0.012	1	20	80	42
6	0.001	0.0012	1	20	80	6
7	—	—	1	20	80	n.d.
8	—	0.12	1	20	80	n.d.
9	0.01	0.012	1	48	80	64
10	0.001	0.0012	1	48	80	7
11	0.1	0.12	1	10	80	77
12	0.1	0.12	1	20	r.t.	72

Reaction conditions: **1a** (0.1 mmol, 1.0 equiv.), **2a** (0.12 mmol, 1.2 equiv.), NaOAc (0.2 mmol, 2.0 equiv.), solvent (1.0 mL). [a] The yields were determined by GC using *n*-hexadecane as the internal standard.

Table S2. Effect of solvent

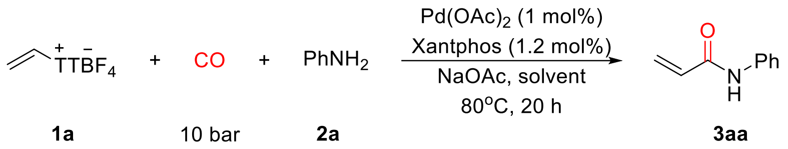

Entry	Solvent	Yield (%)[a]
1	1,4-dioxane	95
2	Tol.	36
3	Tol.+H_2O (1:1)	69
4	DMF	13
5	THF	82
6	DCE	85
7	MeCN	99
8	H_2O	35
9	DMSO	n.d.

Reaction conditions: **1a** (0.1 mmol, 1.0 equiv.), **2a** (0.12 mmol, 1.2 equiv.), $Pd(OAc)_2$ (1.0 mol%), Xantphos (1.2 mol%), NaOAc (0.2 mmol, 2.0 equiv.), solvent (1.0 mL), CO (10 bar), 20 h. [a] The yields were determined by GC using *n*-hexadecane as the internal standard.

Table S3. Effect of base

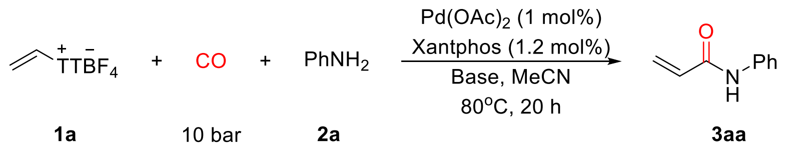

Entry	Base	Yield%[a]
1	—	65
2	NaOAc	99
3	HCOONa	96
4	Na_2CO_3	98
5	$NaHCO_3$	97
6	NaOH	19
7	*t*BuOLi	68
8	Li_2CO_3	26
9	Cs_2CO_3	81
10	K_3PO_4	92
11	TEA	14

Reaction conditions: **1a** (0.1 mmol, 1.0 equiv.), **2a** (0.12 mmol, 1.2 equiv.), $Pd(OAc)_2$ (1.0 mol%), Xantphos (1.2 mol%), base (0.2 mmol, 2.0 equiv.), MeCN (1.0 mL), CO (10 bar), 20 h. [a] The yields were determined by GC using *n*-hexadecane as the internal standard.

3. General Procedures for Synthesis of TT salt

Table S4 **Alkenyl Sulfonium Salts Substrates.**

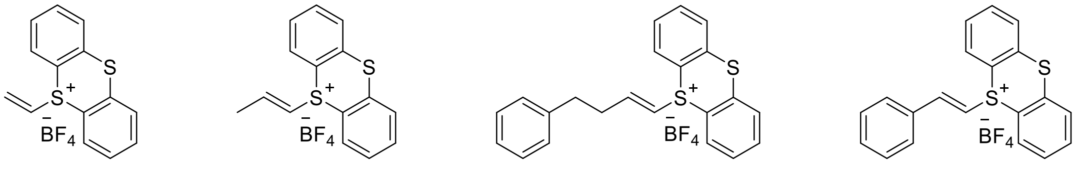

1a **1b** **1c** **1d**

3.1 Synthesis of thianthrenium salts 1a,[1, 2] 1b.[2]

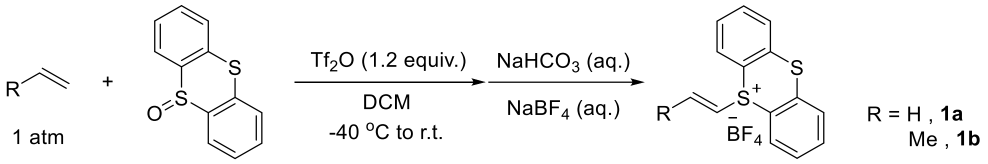

The known thianthrenium salts **1a** and **1b** were prepared according to the reported procedures.[1, 2] To a round-bottom flask equipped with a stirring bar was charged with alkene, thianthrene-S-oxide 2 (5.9 g, 25 mmol, 1.0 equiv.) and DCM (250 mL). The flask was capped with a rubber septum and cooled down to –40°C. A balloon filled with alkene was connected to the flask to maintain the alkene atmosphere throughout the reaction. Triflic anhydride (5.1 mL, 30.2 mmol, 1.20 equiv.) was added dropwise to the reaction, and a dark purple suspension was progressively formed. After 30 minutes, the cooling bath was removed and the mixture was stirred for 1.5 hour at room temperature. The balloon and the rubber septum were removed, and sat. aqueous $NaHCO_3$ (250 mL) was added carefully. The mixture was vigorously shaken in a separation funnel, phases were separated and the aqueous layer was extracted with DCM (3 × 100 mL). All organic phases were combined, washed with aqueous solutions of $NaBF_4$ (3 × 100 mL, 5 % w/w), dried over Na_2SO_4, filtered, and the solvent was removed under reduced pressure. The residue was purified by chromatography on silica gel eluting with MeOH/DCM = 0-10% to afford the thianthrenium salt **1a**, **1b**.

3.2 Synthesis of thianthrenium salt 1c.[2, 3]

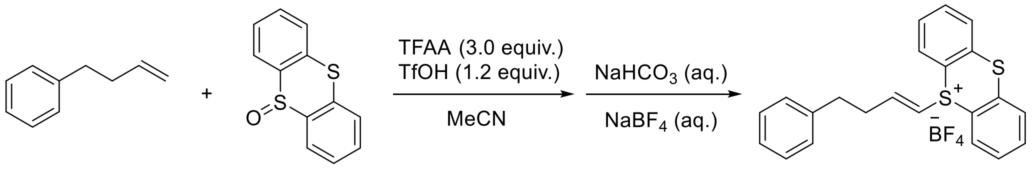

1c

The known thianthrenium salts **1c** were prepared according to the reported procedures.[2, 3] Under nitrogen atmosphere, a 50 mL Schlenk vial equipped with a magnetic stir bar was charged with 4-phenyl-1-butene (1.32 g, 10.0 mmol, 1.0 equiv), thianthrene 5-oxide (2.32 g, 10.0 mmol, 1.0 equiv), and MeCN (50 mL). After cooling to 0 °C, trifluoroacetic anhydride (6.3 g, 30.0 mmol, 3.0 equiv) was added dropwise within 2 minutes, followed by dropwise addition of TfOH (1.8 g, 12.0 mmol, 1.2 equiv) within 10 minutes. After stirring the lilac mixture at 0 °C for 1h followed by stirring for 1h at room temperature, the resulting mixture was concentrated under reduced pressure, and subsequently diluted with DCM. The

saturated aqueous $NaHCO_3$ solution (450 mL) pour into DCM solution, and the layers were separated with separatory funnel. Then, organic phases were washed with aqueous $NaBF_4$ solution (3 × 150 mL, 5 % w/w), dried over Na_2SO_4, filtered, and the solvent was removed under reduced pressure. The residue was purified by chromatography on silica gel eluting with MeOH/DCM = 0-10% to afford the alkenyl thianthrenium salt **1c**.

3.3 Synthesis of thianthrenium salts 1d.[2, 3]

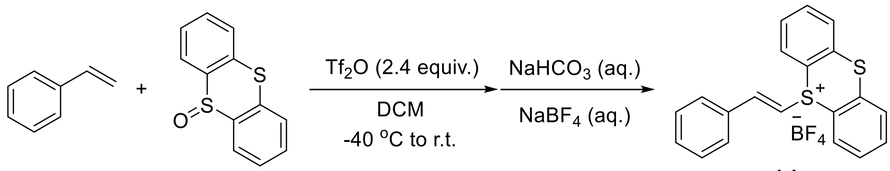

1d

The known thianthrenium salts **1d** were prepared according to the reported procedures.[2, 3] To a round-bottom flask equipped with a stirring bar was charged with styrene (3.0 mmol, 1.0 equiv.), thianthrene-S-oxide (767 mg, 3.3 mmol, 1.1 equiv.) and DCM (20 mL). The flask was capped with a rubber septum and cooled down to -40°C. Triflic anhydride (0.44 mL, 3.0 mmol, 1.0 equiv.) was added dropwise to the reaction, and a dark purple suspension was progressively formed. After 30 minutes, the cooling bath was removed and the mixture was stirred for 1.5 hour at room temperature. The rubber septum was removed, and sat. aqueous $NaHCO_3$ (25 mL) was added carefully. The mixture was vigorously shaken in a separation funnel, phases were separated and the aqueous layer was extracted with DCM (3 × 10 mL). All organic phases were combined, washed with aqueous solutions of $NaBF_4$ (3 × 15 mL, 5 % w/w), dried over Na_2SO_4, filtered, and the solvent was removed under reduced pressure. The residue was purified by chromatography on silica gel eluting with MeOH/DCM = 0-10% to afford the thianthrenium salt **1d**.

4. General Procedures

4.1 General Procedures for

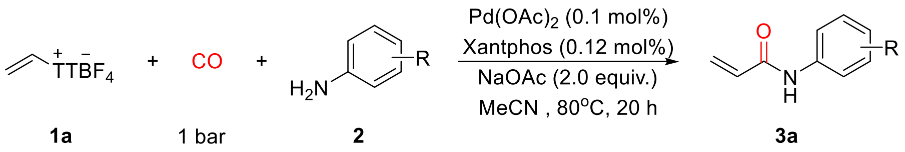

A 4 mL screw-cap vial equipped with a magnetic stirrer bar was charged with $Pd(OAc)_2$ (0.1 mol%), Xantphos (0.12 mol%), TT salt **1** (0.1 mmol, 1.0 equiv.), phenyl amine **2** (0.12 mmol, 1.2 equiv.), NaOAc (16.4 mg, 0.2 mmol, 2.0 equiv.). The vial was closed by PTFE/white rubber septum (Wheaton 13 mm Septa) and phenolic cap and connected with atmosphere with a needle. The vial was evacuated under vacuum and recharged with N_2 for three times. Then, MeCN (1.0 mL) was injected under N_2 atmosphere by using a syringe. After that the vial (or several vials) was placed in an alloy plate, which was transferred into a 300 mL autoclave of the 4560 series from Parr Instruments under N_2 atmosphere. After flushing the autoclave three times with CO, a pressure of 1 bar of CO was adjusted at ambient temperature. Then, the reaction was performed for 20 h at 80 °C. After the reaction was complete, the autoclave was cooled down to room temperature and the pressure was released carefully. The mixture was concentrated under reduced pressure. The crude residue was purified by flash chromatography using petroleum ether/ethyl acetate to afford the corresponding products.

4.2 Procedure for 2.0 mmol Scale Reaction

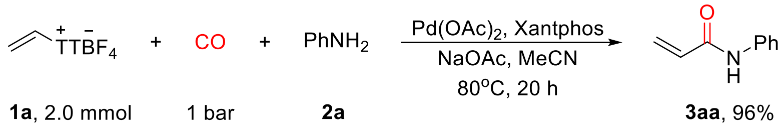

A 25 mL screw-cap vial equipped with a magnetic stirrer bar was charged with $Pd(OAc)_2$ (0.1 mol%), Xantphos (0.12 mol%), TT salt **1a** (660 mg, 2.0 mmol, 1.0 equiv.), phenyl amine **2a** (220 μL, 2.2 mmol, 1.1 equiv.), NaOAc (16.4 mg, 0.2 mmol, 2.0 equiv.). The vial was closed by PTFE/white rubber septum (Wheaton 13 mm Septa) and phenolic cap and connected with atmosphere with a needle. The vial was evacuated under vacuum and recharged with N_2 for three times. Then, MeCN (10 mL) was injected under N_2 atmosphere by using a syringe. After that the vial (or several vials) was placed in an alloy plate, which was transferred into a 300 mL autoclave of the 4560 series from Parr Instruments under N_2 atmosphere. After flushing the autoclave three times with CO, a pressure of 1 bar of CO was adjusted at ambient temperature. Then, the reaction was performed for 20 h at 80 °C. After the reaction was complete, the autoclave was cooled down to room temperature and the pressure was released carefully. The mixture was concentrated under reduced pressure. The crude residue was purified by flash chromatography using petroleum ether/ethyl acetate 3/1 to afford the pure products (282 mg, white solid, 96% yield).

5. Characterization Data

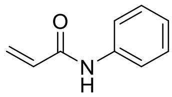

***N*-phenylacrylamide (3aa)**

14.7 mg, 99% yield, white solid. Eluent: petroleum ether/ethyl acetate = 3:1.

^{1}H NMR (700 MHz, DMSO) δ 10.17 (s, 1H), 7.72 (d, *J* = 17.9 Hz, 2H), 7.34 (t, *J* = 7.9 Hz, 2H), 7.08 (t, *J* = 7.4 Hz, 1H), 6.52 – 6.44 (m, 1H), 6.34 – 6.27 (m, 1H), 5.78 – 5.75 (m, 1H).

^{13}C NMR (176 MHz, DMSO) δ 163.1, 139.0, 131.9, 128.7, 126.7, 123.4, 119.3.

HRMS (ESI-TOF) m/z: [M+H]$^+$: Calcd. for $C_9H_{10}NO^+$ 148.0757, Found: 148.0758.

m.p. 103-106 ºC

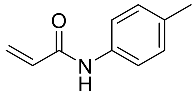

***N*-(*p*-tolyl)acrylamide (3ab)**

15.6 mg, 97% yield, white solid. Eluent: petroleum ether/ethyl acetate = 3:1.

^{1}H NMR (700 MHz, DMSO) δ 10.05 (s, 1H), 7.50 (s, 1H), 7.46 (d, *J* = 8.1 Hz, 1H), 7.20 (t, *J* = 7.8 Hz, 1H), 6.89 (d, *J* = 7.5 Hz, 1H), 6.43 (dd, *J* = 17.0, 10.1 Hz, 1H), 6.25 (dd, *J* = 17.0, 1.9 Hz, 1H), 5.74 (dd, *J* = 10.2, 1.9 Hz, 1H), 2.28 (s, 3H).

^{13}C NMR (176 MHz, DMSO) δ 163.0, 138.8, 137.8, 131.8, 128.5, 126.6, 124.1, 119.7, 116.4, 21.1.

HRMS (ESI-TOF) m/z: [M+H]$^+$: Calcd. for $C_{10}H_{12}NO^+$ 162.0913, Found: 162.0912.

m.p. 141-145 ºC

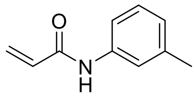

***N*-(*m*-tolyl)acrylamide (3ac)**

16.0 mg, 99% yield, white solid. Eluent: petroleum ether/ethyl acetate = 3:1.

^{1}H NMR (700 MHz, DMSO) δ 10.04 (s, 1H), 7.55 (d, *J* = 8.3 Hz, 2H), 7.12 (d, *J* = 8.2 Hz, 2H), 6.42 (dd, *J* = 16.9, 10.2 Hz, 1H), 6.24 (dd, *J* = 17.0, 2.0 Hz, 1H), 5.73 (dd, *J* = 10.1, 1.9 Hz, 1H), 2.26 (s, 3H).

^{13}C NMR (176 MHz, DMSO) δ 162.8, 136.4, 132.3, 131.9, 129.0, 126.4, 119.2, 20.4.

HRMS (ESI-TOF) m/z: [M+H]$^+$: Calcd. for $C_{10}H_{12}NO^+$ 162.0913, Found: 162.0912.

m.p. 136-137 ºC

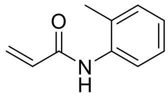

***N*-(*o*-tolyl)acrylamide (3ad)**

15.3 mg, 95% yield, white solid. Eluent: petroleum ether/ethyl acetate = 3:1.

^{1}H NMR (700 MHz, DMSO) δ 9.47 (s, 1H), 7.47 (d, *J* = 7.8 Hz, 1H), 7.22 (d, *J* = 7.4 Hz, 1H), 7.17 (t, *J* = 7.5 Hz, 1H), 7.09 (t, *J* = 7.4 Hz, 1H), 6.57 – 6.50 (m, 1H), 6.24 (dd, *J* = 17.0, 1.8 Hz, 1H), 5.76 – 5.73 (m, 1H), 2.21 (s, 3H).

^{13}C NMR (176 MHz, DMSO) δ 163.1, 136.0, 131.7, 131.5, 130.2, 126.4, 125.8, 125.2, 124.8, 17.8.

HRMS (ESI-TOF) m/z: [M+H]$^+$: Calcd. for $C_{10}H_{12}NO^+$ 162.0913, Found: 162.0918.

m.p. 109-112 ºC

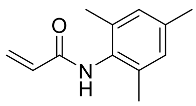

***N*-mesitylacrylamide (3ae)**

18.8 mg, 97% yield, white solid. Eluent: petroleum ether/ethyl acetate = 3:1.

^{1}H NMR (700 MHz, DMSO) δ 9.36 (s, 1H), 6.88 (s, 2H), 6.46 (dd, *J* = 17.1, 10.3 Hz, 1H), 6.20 (dd, *J* = 17.1, 1.7 Hz, 1H), 5.71 (dd, *J* = 10.3, 1.8 Hz, 1H), 2.22 (s, 3H), 2.08 (s, 6H).

^{13}C NMR (176 MHz, DMSO) δ 163.0, 135.3, 134.6, 132.1, 131.4, 128.2, 125.9, 20.4, 17.9.

HRMS (ESI-TOF) m/z: [M+H]$^{+}$: Calcd. for $C_{12}H_{16}NO^{+}$ 190.1226, Found: 190.1265.

m.p. 157-159 ºC

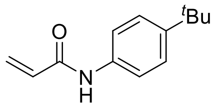

***N*-(4-(*tert*-butyl)phenyl)acrylamide (3af)**

20.2 mg, 99% yield, white solid. Eluent: petroleum ether/ethyl acetate = 3:1.

^{1}H NMR (700 MHz, DMSO) δ 10.06 (s, 1H), 7.58 (d, *J* = 8.6 Hz, 2H), 7.33 (d, *J* = 8.6 Hz, 2H), 6.42 (dd, *J* = 17.0, 10.2 Hz, 1H), 6.24 (dd, *J* = 17.0, 1.9 Hz, 1H), 5.73 (dd, *J* = 10.1, 1.8 Hz, 1H), 1.26 (s, 9H).

^{13}C NMR (176 MHz, DMSO) δ 162.8, 145.7, 136.3, 131.9, 126.5, 125.3, 119.0, 33.9, 31.1.

HRMS (ESI-TOF) m/z: [M+H]$^{+}$: Calcd. for $C_{13}H_{18}NO^{+}$ 204.1383, Found: 204.1384.

m.p. 96-98 ºC

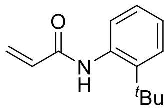

***N*-(2-(*tert*-butyl)phenyl)acrylamide (3ag)**

19.5 mg, 96% yield, white solid. Eluent: petroleum ether/ethyl acetate = 3:1.

^{1}H NMR (700 MHz, DMSO) δ 9.42 (s, 1H), 7.43 – 7.39 (m, 1H), 7.22 (dt, *J* = 13.0, 7.2 Hz, 2H), 7.05 (d, *J* = 7.0 Hz, 1H), 6.54 (dd, *J* = 17.1, 10.3 Hz, 1H), 6.20 (dd, *J* = 17.1, 1.9 Hz, 1H), 5.72 (d, *J* = 10.3 Hz, 1H), 1.31 (s, 9H).

^{13}C NMR (176 MHz, DMSO) δ 164.3, 146.3, 135.5, 131.8, 131.4, 126.9, 126.5, 126.2, 126.1, 34.6, 30.6.

HRMS (ESI-TOF) m/z: [M+H]$^{+}$: Calcd. for $C_{13}H_{18}NO^{+}$ 204.1383, Found: 204.1384.

m.p. 110-112 ºC

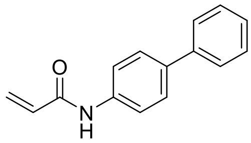

***N*-([1,1'-biphenyl]-4-yl)acrylamide (3ah)**

22.0 mg, 98% yield, white solid. Eluent: petroleum ether/ethyl acetate = 3:1.

^{1}H NMR (700 MHz, DMSO) δ 10.25 (s, 1H), 7.78 (d, *J* = 8.5 Hz, 2H), 7.65 (d, *J* = 8.3 Hz, 4H), 7.45 (t, *J* = 7.7 Hz, 2H), 7.33 (t, *J* = 7.4 Hz, 1H), 6.47 (dd, *J* = 17.0, 10.2 Hz, 1H), 6.29 (dd, *J* = 17.0, 1.7 Hz, 1H), 5.78 (dd, *J* = 10.1, 1.7 Hz, 1H).

^{13}C NMR (176 MHz, DMSO) δ 163.1, 139.6, 138.4, 135.0, 131.7, 128.8, 127.0, 126.9, 126.2, 119.6.

HRMS (ESI-TOF) m/z: [M+H]$^{+}$: Calcd. for $C_{15}H_{14}NO^{+}$ 224.1070, Found: 224.1070.

m.p. 164-169 ºC

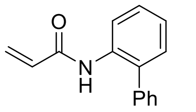

***N*-([1,1'-biphenyl]-2-yl)acrylamide (3ai)**

21.0 mg, 92% yield, white solid. Eluent: petroleum ether/ethyl acetate = 3:1.

^{1}H NMR (700 MHz, DMSO) δ 9.50 (s, 1H), 7.50 (d, *J* = 7.8 Hz, 1H), 7.43 – 7.41 (m, 2H), 7.38 – 7.32 (m, 6H), 6.31 (dd, *J* = 17.0, 10.2 Hz, 1H), 6.15 (d, *J* = 16.8 Hz, 1H), 5.66 (d, *J* = 10.2 Hz, 1H).

^{13}C NMR (176 MHz, DMSO) δ 164.3, 139.0, 137.1, 134.5, 131.7, 130.6, 128.9, 128.6, 128.0, 127.5, 126.9, 126.6.

HRMS (ESI-TOF) m/z: [M+H]$^{+}$: Calcd. for $C_{15}H_{14}NO^{+}$ 224.1070, Found: 224.1072.

m.p. 130-132 °C

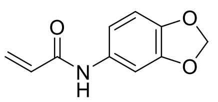

***N*-(benzo[*d*][1,3]dioxol-5-yl)acrylamide (3aj)**

18.7 mg, 98% yield, white solid. Eluent: petroleum ether/ethyl acetate = 3:1.

^{1}H NMR (700 MHz, DMSO) δ 10.05 (s, 1H), 7.40 (d, *J* = 1.7 Hz, 1H), 7.03 (dd, *J* = 8.4, 1.7 Hz, 1H), 6.87 (d, *J* = 8.4 Hz, 1H), 6.38 (dd, *J* = 17.0, 10.1 Hz, 1H), 6.23 (dd, *J* = 17.0, 1.7 Hz, 1H), 5.99 (s, 2H), 5.73 (dd, *J* = 10.1, 1.7 Hz, 1H).

^{13}C NMR (176 MHz, DMSO) δ 162.7, 147.0, 143.0, 133.3, 131.7, 126.5, 112.1, 108.0, 101.3, 100.9.

HRMS (ESI-TOF) m/z: [M+H]$^{+}$: Calcd. for $C_{10}H_{9}NO_{3}^{+}$ 192.0655, Found: 192.0660.

m.p. 157-159 °C

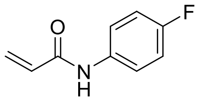

***N*-(4-fluorophenyl)acrylamide (3ak)**

16.0 mg, 96% yield, white solid. Eluent: petroleum ether/ethyl acetate = 3:1.

^{1}H NMR (700 MHz, DMSO) δ 10.19 (s, 1H), 7.73 – 7.64 (m, 2H), 7.17 (t, *J* = 8.8 Hz, 2H), 6.41 (dd, *J* = 17.0, 10.2 Hz, 1H), 6.26 (d, *J* = 16.8 Hz, 1H), 5.76 (d, *J* = 10.2 Hz, 1H).

^{13}C NMR (176 MHz, DMSO) δ 162.92, 158.01 (d, *J* = 240.0 Hz), 135.31 (d, *J* = 2.4 Hz), 131.62, 126.84, 120.95 (d, *J* = 7.8 Hz), 115.26 (d, *J* = 22.2 Hz).

^{19}F NMR (376 MHz, DMSO) δ -119.0.

HRMS (ESI-TOF) m/z: [M+H]$^{+}$: Calcd. for $C_{9}H_{9}FNO^{+}$ 166.0663, Found: 166.0666.

m.p. 146-149 °C

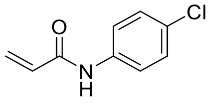

***N*-(4-chlorophenyl)acrylamide (3al)**

17.0 mg, 94% yield, white solid. Eluent: petroleum ether/ethyl acetate = 3:1.

^{1}H NMR (700 MHz, DMSO) δ 10.27 (s, 1H), 7.70 (d, *J* = 8.9 Hz, 2H), 7.38 (d, *J* = 8.8 Hz, 2H), 6.42 (dd, *J* = 17.0, 10.2 Hz, 1H), 6.27 (dd, *J* = 17.0, 1.9 Hz, 1H), 5.78 (dd, *J* = 10.1, 1.9 Hz, 1H).

^{13}C NMR (176 MHz, DMSO) δ 163.1, 137.9, 131.5, 128.6, 127.2, 126.9, 120.8.

HRMS (ESI-TOF) m/z: [M+H]$^{+}$: Calcd. for $C_{9}H_{9}ClNO^{+}$ 182.0367, Found: 182.0370.

m.p. 188-192 °C

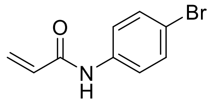

***N*-(4-bromophenyl)acrylamide (3am)**

22.4 mg, 99% yield, white solid. Eluent: petroleum ether/ethyl acetate = 3:1.

^{1}H NMR (700 MHz, DMSO) δ 10.26 (s, 1H), 7.57 (d, *J* = 91.8 Hz, 4H), 6.45 – 6.21 (m, 2H), 5.77 (s, 1H).

^{13}C NMR (176 MHz, DMSO) δ 163.1, 138.3, 131.5, 127.2, 121.1, 115.0.

HRMS (ESI-TOF) m/z: [M+H]$^+$: Calcd. for $C_9H_9BrNO^+$ 225.9862, Found: 225.9854.

m.p. 194-196 ºC

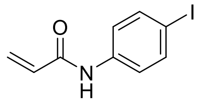

***N*-(4-iodophenyl)acrylamide (3an)**

24.5 mg, 90% yield, white solid. Eluent: petroleum ether/ethyl acetate = 3:1.

^{1}H NMR (700 MHz, DMSO) δ 10.23 (s, 1H), 7.66 (d, *J* = 8.7 Hz, 2H), 7.51 (d, *J* = 8.5 Hz, 2H), 6.41 (dd, *J* = 17.0, 10.1 Hz, 1H), 6.26 (dd, *J* = 17.0, 1.4 Hz, 1H), 5.77 (dd, *J* = 10.2, 1.5 Hz, 1H).

^{13}C NMR (176 MHz, DMSO) δ 163.1, 138.7, 137.3, 131.5, 127.2, 121.4, 87.0.

HRMS (ESI-TOF) m/z: [M+H]$^+$: Calcd. for $C_9H_9INO_2^+$ 273.9723, Found: 273.9730.

m.p. 202-205 ºC

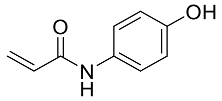

***N*-(4-hydroxyphenyl)acrylamide (3ao)**

10.3 mg, 63% yield, white solid. Eluent: petroleum ether/ethyl acetate = 2:1.

^{1}H NMR (700 MHz, DMSO) δ 9.88 (s, 1H), 9.23 (s, 1H), 7.44 (d, *J* = 8.8 Hz, 2H), 6.71 (d, *J* = 8.8 Hz, 2H), 6.39 (dd, *J* = 17.0, 10.2 Hz, 1H), 6.20 (dd, *J* = 17.0, 1.9 Hz, 1H), 5.69 (dd, *J* = 10.1, 1.8 Hz, 1H).

^{13}C NMR (176 MHz, DMSO) δ 162.4, 153.4, 132.0, 130.6, 125.9, 120.9, 115.0.

HRMS (ESI-TOF) m/z: [M+H]$^+$: Calcd. for $C_9H_{10}NO_2^+$ 164.0706, Found: 164.0705.

m.p. 185-190 ºC

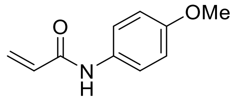

***N*-(4-methoxyphenyl)acrylamide (3ap)**

15.9 mg, % yield, white solid. Eluent: petroleum ether/ethyl acetate = 3:1.

^{1}H NMR (700 MHz, DMSO) δ 10.00 (s, 1H), 7.58 (d, *J* = 9.0 Hz, 2H), 6.90 (d, *J* = 9.0 Hz, 2H), 6.40 (dd, *J* = 17.0, 10.2 Hz, 1H), 6.23 (dd, *J* = 17.0, 1.9 Hz, 1H), 5.71 (dd, *J* = 10.1, 1.9 Hz, 1H), 3.73 (s, 3H).

^{13}C NMR (176 MHz, DMSO) δ 162.6, 155.3, 132.1, 131.9, 126.2, 120.7, 113.8, 55.1.

HRMS (ESI-TOF) m/z: [M+H]$^+$: Calcd. for $C_{10}H_{12}NO_2^+$ 178.0863, Found: 178.0863.

m.p. 97-100 ºC

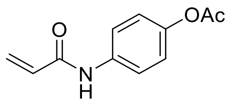

4-acrylamidophenyl acetate (3aq)

19.9 mg, 97% yield, white solid. Eluent: petroleum ether/ethyl acetate = 3:1.

^{1}H NMR (700 MHz, DMSO) δ 10.21 (s, 1H), 7.69 (d, *J* = 8.9 Hz, 2H), 7.09 (d, *J* = 8.9 Hz, 2H), 6.43 (dd, *J* = 17.0, 10.2 Hz, 1H), 6.27 (dd, *J* = 17.0, 1.7 Hz, 1H), 5.76 (dd, *J* = 10.2, 1.7 Hz, 1H), 2.25 (s, 3H).

^{13}C NMR (176 MHz, DMSO) δ 169.2, 163.0, 146.0, 136.5, 131.7, 126.9, 121.9, 120.1, 20.7.

HRMS (ESI-TOF) m/z: [M+H] $^+$: Calcd. for $C_{11}H_{12}NO_3^+$ 206.0812, Found: 206.0811.

m.p. 145-147 ºC

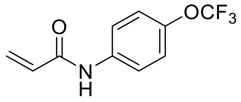

***N*-(4-(trifluoromethoxy)phenyl)acrylamide (3ar)**

23.0 mg, 99% yield, white solid. Eluent: petroleum ether/ethyl acetate = 3:1.

^{1}H NMR (700 MHz, DMSO) δ 10.33 (s, 1H), 7.77 (d, *J* = 8.7 Hz, 2H), 7.34 (d, *J* = 8.4 Hz, 2H), 6.43 (dd, *J* = 16.9, 10.2 Hz, 1H), 6.28 (d, *J* = 16.9 Hz, 1H), 5.79 (d, *J* = 10.1 Hz, 1H).

^{13}C NMR (176 MHz, DMSO) δ 163.2, 143.6, 138.1, 131.5, 127.3, 121.6, 120.6, 119.5 (q, *J* = 255.2 Hz).

^{19}F NMR (376 MHz, DMSO) δ -60.4.

HRMS (ESI-TOF) m/z: [M+H] $^+$: Calcd. for $C_{10}H_9F_3NO_2^+$ 232.0580, Found: 232.0581.

m.p. 133-135 ºC

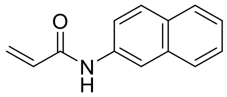

***N*-(naphthalen-2-yl)acrylamide (3as)**

19.5 mg, 99% yield, white solid. Eluent: petroleum ether/ethyl acetate = 3:1.

^{1}H NMR (700 MHz, DMSO) δ 10.36 (s, 1H), 8.40 (s, 1H), 7.90 – 7.82 (m, 3H), 7.66 (d, *J* = 8.6 Hz, 1H), 7.45 (dt, *J* = 46.7, 7.2 Hz, 2H), 6.51 (dd, *J* = 16.9, 10.1 Hz, 1H), 6.32 (d, *J* = 16.9 Hz, 1H), 5.80 (d, *J* = 10.1 Hz, 1H).

^{13}C NMR (176 MHz, DMSO) δ 163.3, 136.5, 133.3, 131.7, 129.8, 128.3, 127.4, 127.3, 126.9, 126.4, 124.6, 119.9, 115.4.

HRMS (ESI-TOF) m/z: [M+H] $^+$: Calcd. for $C_{13}H_{12}NO^+$ 198.0913, Found: 198.0916.

m.p. 122-124 ºC

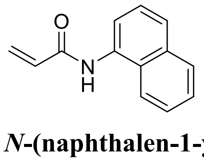

***N*-(naphthalen-1-yl)acrylamide (3at)**

18.6 mg, 94% yield, white solid. Eluent: petroleum ether/ethyl acetate = 3:1.

^{1}H NMR (700 MHz, DMSO) δ 10.53 (s, 1H), 8.17 (s, 1H), 7.95 (s, 1H), 7.78 (d, *J* = 7.3 Hz, 2H), 7.57 – 7.49 (m, 3H), 7.03 – 6.87 (m, 1H), 6.30 (d, *J* = 17.0 Hz, 1H), 5.79 (d, *J* = 9.4 Hz, 1H).

^{13}C NMR (176 MHz, DMSO) δ 163.9, 133.6, 133.3, 131.9, 128.0, 127.5, 126.5, 125.9, 125.7, 125.4, 125.1, 122.9, 121.4.

HRMS (ESI-TOF) m/z: [M+H] $^+$: Calcd. for $C_{13}H_{12}NO^+$ 198.0913, Found: 198.0914.

m.p. 141-143 ºC

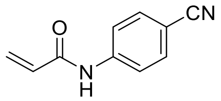

***N*-(4-cyanophenyl)acrylamide (3au)**

14.1 mg, 76% yield, white solid. Eluent: petroleum ether/ethyl acetate = 3:1.

^{1}H NMR (700 MHz, DMSO) δ 10.57 (s, 1H), 7.85 (d, *J* = 8.7 Hz, 2H), 7.80 (d, *J* = 8.7 Hz, 2H), 6.45 (dd, *J* = 17.0, 10.2 Hz, 1H), 6.33 (dd, *J* = 17.0, 1.6 Hz, 1H), 5.84 (dd, *J* = 10.2, 1.6 Hz, 1H).

^{13}C NMR (176 MHz, DMSO) δ 163.7, 143.1, 133.2, 131.2, 128.2, 119.3, 118.9, 105.1.

HRMS (ESI-TOF) m/z: [M+H]$^+$: Calcd. for $C_{10}H_9N_2O^+$ 173.0709, Found: 173.0725.

m.p. 198-201 ºC

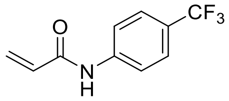

***N*-(4-(trifluoromethyl)phenyl)acrylamide (3av)**

21.3 mg, 99% yield, white solid. Eluent: petroleum ether/ethyl acetate = 3:1.

^{1}H NMR (700 MHz, DMSO) δ 10.50 (s, 1H), 7.89 (d, *J* = 8.5 Hz, 2H), 7.70 (d, *J* = 8.6 Hz, 2H), 6.47 (dd, *J* = 17.0, 10.2 Hz, 1H), 6.33 (dd, *J* = 17.0, 1.7 Hz, 1H), 5.83 (dd, *J* = 10.2, 1.6 Hz, 1H).

^{13}C NMR (176 MHz, DMSO) δ 163.6, 142.5, 131.4, 127.8, 126.0 (q, *J* = 3.5 Hz), 124.3 (q, *J* = 269.3 Hz), 123.4 (q, *J* = 31.7 Hz), 119.2.

^{19}F NMR (376 MHz, DMSO) δ -57.0.

HRMS (ESI-TOF) m/z: [M+H]$^+$: Calcd. for $C_{10}H_9F_3NO^+$ 216.0631, Found: 216.0632.

m.p. 156-159 ºC

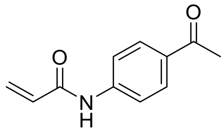

***N*-(4-acetylphenyl)acrylamide (3aw)**

15.5 mg, 82% yield, white solid. Eluent: petroleum ether/ethyl acetate = 3:1.

^{1}H NMR (700 MHz, DMSO) δ 10.47 (s, 1H), 7.95 (d, *J* = 8.7 Hz, 2H), 7.81 (d, *J* = 8.7 Hz, 2H), 6.47 (dd, *J* = 17.0, 10.2 Hz, 1H), 6.31 (dd, *J* = 17.0, 1.5 Hz, 1H), 5.82 (dd, *J* = 10.2, 1.5 Hz, 1H), 2.54 (s, 3H).

^{13}C NMR (176 MHz, DMSO) δ 196.4, 163.5, 143.2, 131.8, 131.4, 129.4, 127.7, 118.5, 26.3.

HRMS (ESI-TOF) m/z: [M+H]$^+$: Calcd. for $C_{11}H_{12}NO_2^+$ 190.0863, Found: 190.0860.

m.p. 140-144 ºC

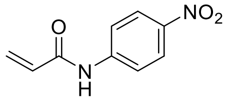

***N*-(4-nitrophenyl)acrylamide (3ax)**

19.2 mg, 99% yield, white solid. Eluent: petroleum ether/ethyl acetate = 3:1.

^{1}H NMR (700 MHz, DMSO) δ 10.74 (s, 1H), 8.26 – 8.23 (m, 2H), 7.93 – 7.91 (m, 2H), 6.47 (dd, *J* = 17.0, 10.1 Hz, 1H), 6.35 (dd, *J* = 17.0, 1.6 Hz, 1H), 5.87 (dd, *J* = 10.1, 1.6 Hz, 1H).

^{13}C NMR (176 MHz, DMSO) δ 163.8, 145.1, 142.3, 131.1, 128.5, 124.9, 119.0.

HRMS (ESI-TOF) m/z: [M+H]$^+$: Calcd. for $C_9H_9N_2O_3^+$ 193.0608, Found: 193.0687.

m.p. 229-232 ºC

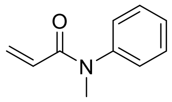

***N*-methyl-N-phenylacrylamide (4aa)**

14.8 mg, 92% yield, white solid. Eluent: petroleum ether/ethyl acetate = 3:1.

^{1}H NMR (700 MHz, DMSO) δ 7.46 (t, *J* = 7.7 Hz, 2H), 7.37 (t, *J* = 7.3 Hz, 1H), 7.29 (d, *J* = 7.4 Hz, 2H), 6.17 – 6.14 (m, 1H), 6.03 (s, 1H), 5.57 (d, *J* = 10.7 Hz, 1H), 3.25 (s, 3H).

^{13}C NMR (176 MHz, DMSO) δ 164.2, 143.0, 129.5, 128.5, 127.4, 127.1, 127.1, 36.8.

HRMS (ESI-TOF) m/z: [M+H]$^+$: Calcd. for $C_{10}H_{12}NO^+$ 162.0913, Found: 162.0913.

m.p. 75-78 ºC

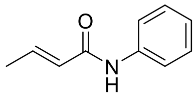

(*E*)-*N*-phenylbut-2-enamide (3ba)

15.1 mg, 94% yield, white solid. Eluent: petroleum ether/ethyl acetate = 3:1.

^{1}H NMR (700 MHz, DMSO) δ 9.92 (s, 1H), 7.64 (d, *J* = 7.9 Hz, 2H), 7.30 (t, *J* = 7.9 Hz, 2H), 7.03 (t, *J* = 7.3 Hz, 1H), 6.82 – 6.76 (m, 1H), 6.14 – 6.11 (m, 1H), 1.88 – 1.86 (m, 3H).

^{13}C NMR (176 MHz, DMSO) δ 163.3, 139.7, 139.2, 128.6, 125.9, 123.0, 119.1, 17.4.

HRMS (ESI-TOF) m/z: [M+H]$^+$: Calcd. for $C_{10}H_{12}NO^+$ 162.0913, Found: 162.0914.

m.p. 113-118 ºC

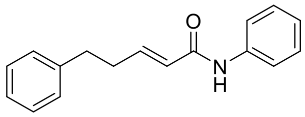

(*E*)-*N*,5-diphenylpent-2-enamide (3ca)

22.1 mg, 88% yield, white solid. Eluent: petroleum ether/ethyl acetate = 3:1.

^{1}H NMR (700 MHz, DMSO) δ 9.95 (s, 1H), 7.63 (d, *J* = 7.9 Hz, 2H), 7.32 – 7.28 (m, 4H), 7.25 (d, *J* = 7.3 Hz, 2H), 7.19 (t, *J* = 7.3 Hz, 1H), 7.03 (t, *J* = 7.4 Hz, 1H), 6.87 – 6.77 (m, 1H), 6.12 (d, *J* = 15.3 Hz, 1H), 2.77 (t, *J* = 7.6 Hz, 2H), 2.55 – 2.51 (m, 2H).

^{13}C NMR (176 MHz, DMSO) δ 163.3, 143.6, 140.9, 139.2, 128.6, 128.2, 128.2, 125.8, 124.9, 123.1, 119.1, 33.7, 33.0.

HRMS (ESI-TOF) m/z: [M+H]$^+$: Calcd. for $C_{17}H_{18}NO^+$ 252.1383, Found: 252.1386.

m.p. 115-117 ºC

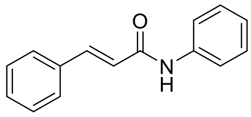

***N*-phenylcinnamamide (3da)**

22.3 mg, 99% yield, white solid. Eluent: petroleum ether/ethyl acetate = 3:1.

^{1}H NMR (700 MHz, DMSO) δ 10.20 (s, 1H), 7.70 (d, *J* = 7.9 Hz, 2H), 7.63 (d, *J* = 7.3 Hz, 2H), 7.59 (d, *J* = 15.7 Hz, 1H), 7.45 (t, *J* = 7.3 Hz, 2H), 7.41 (t, *J* = 7.2 Hz, 1H), 7.34 (t, *J* = 7.9 Hz, 2H), 7.07 (t, *J* = 7.3 Hz, 1H), 6.84 (d, *J* = 15.7 Hz, 1H).

^{13}C NMR (176 MHz, DMSO) δ 163.5, 140.1, 139.3, 134.7, 129.8, 129.0, 128.8, 127.7, 123.4, 122.3, 119.2.

HRMS (ESI-TOF) m/z: [M+H]$^+$: Calcd. for $C_{15}H_{14}NO^+$ 224.1070, Found: 224.1070.

m.p. 148-151 ºC

6. Mechanism study experiment

6.1 Radical quenching experiment

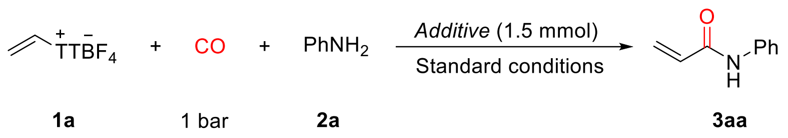

Entry	*Additive*	Yield (%)[a]
1.	—	99
2.	TEMPO	27
3.	BHT	89
4.	DPE	94

Reaction conditions: **1a** (0.1 mmol, 1.0 equiv.), **2a** (0.12 mmol, 1.2 equiv.), $Pd(OAc)_2$ (0.1 mol%), Xantphos (0.12 mol%), NaOAc (0.2 mmol, 2.0 equiv.), MeCN (1 mL), CO (1 bar), *Additives* (0.15 mmol, 1.5 equiv), 20 h. [a] Isolated yield.

6.2 Conditions without CO.

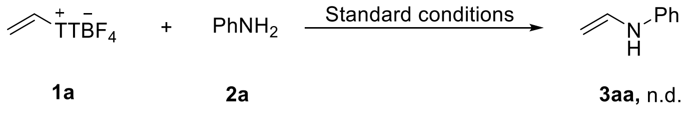

Reaction conditions: **1a** (0.1 mmol, 1.0 equiv.), **2a** (0.12 mmol, 1.2 equiv.), $Pd(OAc)_2$ (0.1 mol%), Xantphos (0.12 mol%), NaOAc (0.2 mmol, 2.0 equiv.), MeCN (1 mL), N_2 (1 bar), 20 h. The reaction was monitored by GC-MS.

7. References

1. Julia, F.; Yan, J.; Paulus, F.; Ritter, T. Vinyl Thianthrenium Tetrafluoroborate: A Practical and Versatile Vinylating Reagent Made from Ethylene. *J. Am. Chem. Soc.* **2021**, *143*, 12992-12998.
2. Liu, M. S.; Du, H. W.; Cui, J. F.; Shu, W. Intermolecular Metal-Free Cyclopropanation and Aziridination of Alkenes with XH(2) (X=N, C) by Thianthrenation. *Angew. Chem. Int. Ed. Engl.* **2022**, *61*, e202209929.
3. Xie, R.; Zhu, J.; Huang, Y. Cu-Catalyzed highly selective silylation and borylation of alkenylsulfonium salts. *Organic Chemistry Frontiers* **2021**, *8*, 5699-5704.

8. Copies of NMR Spectra

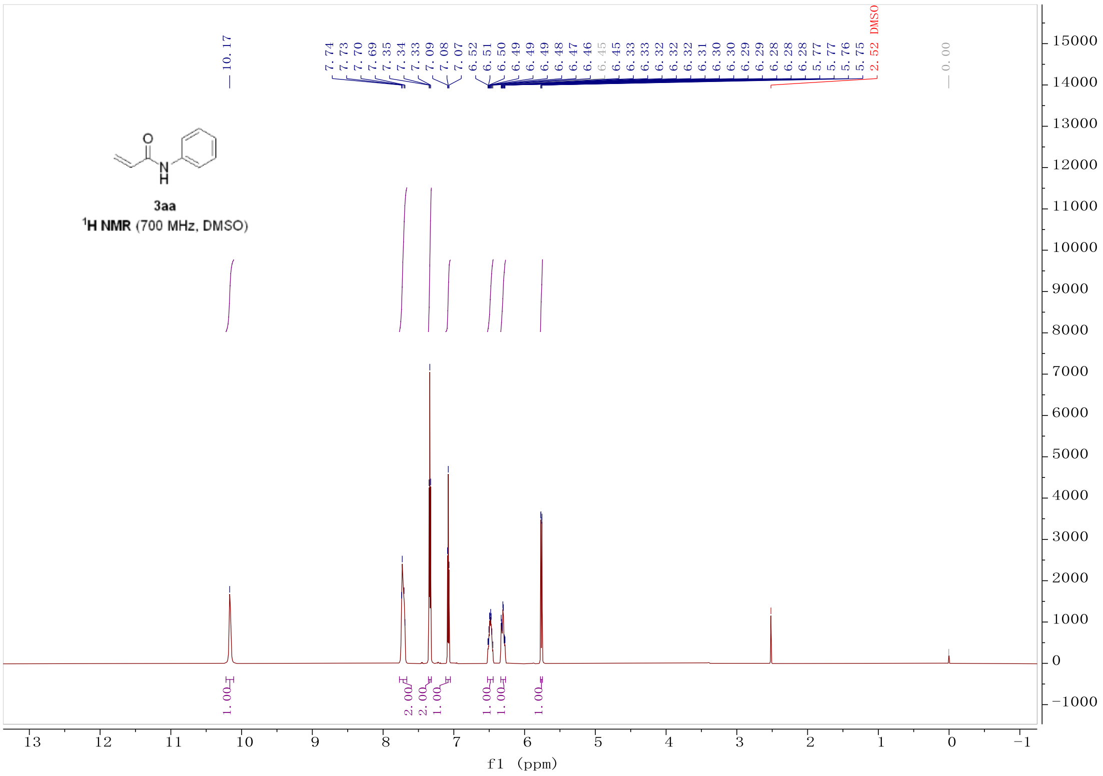

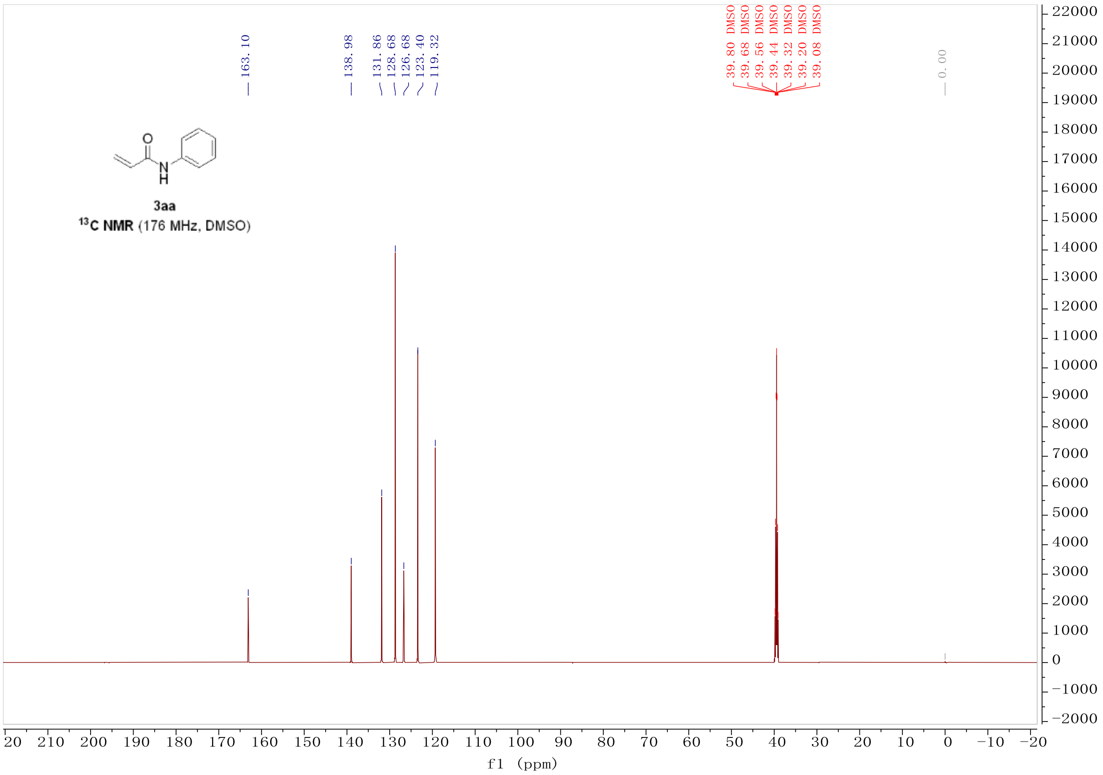

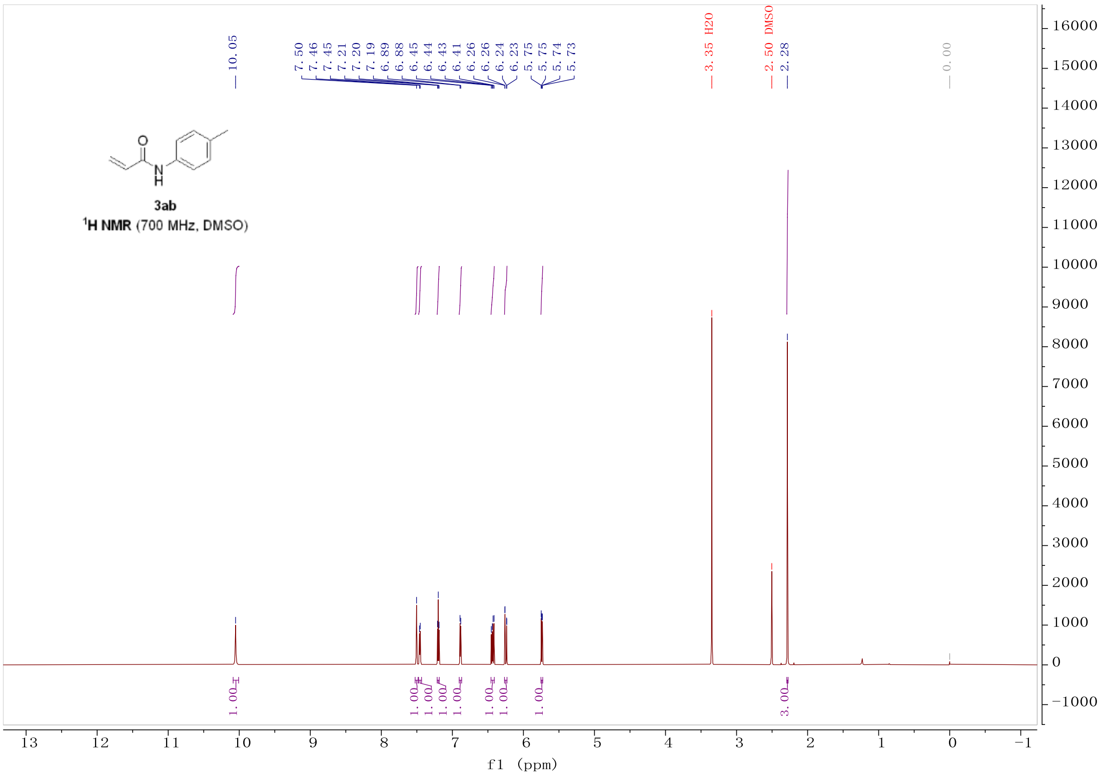
3ab
1H NMR (700 MHz, DMSO)
10.05
7.50 7.46 7.45 7.21 7.20 7.19 6.89 6.88 6.45 6.44 6.43 6.41 6.26 6.24 6.23 5.75 5.75 5.74 5.73
3.35 H2O
2.50 DMSO
2.28
0.00
1.00 1.00 1.00 1.00 1.00 1.00 1.00 1.00 3.00
f1 (ppm)

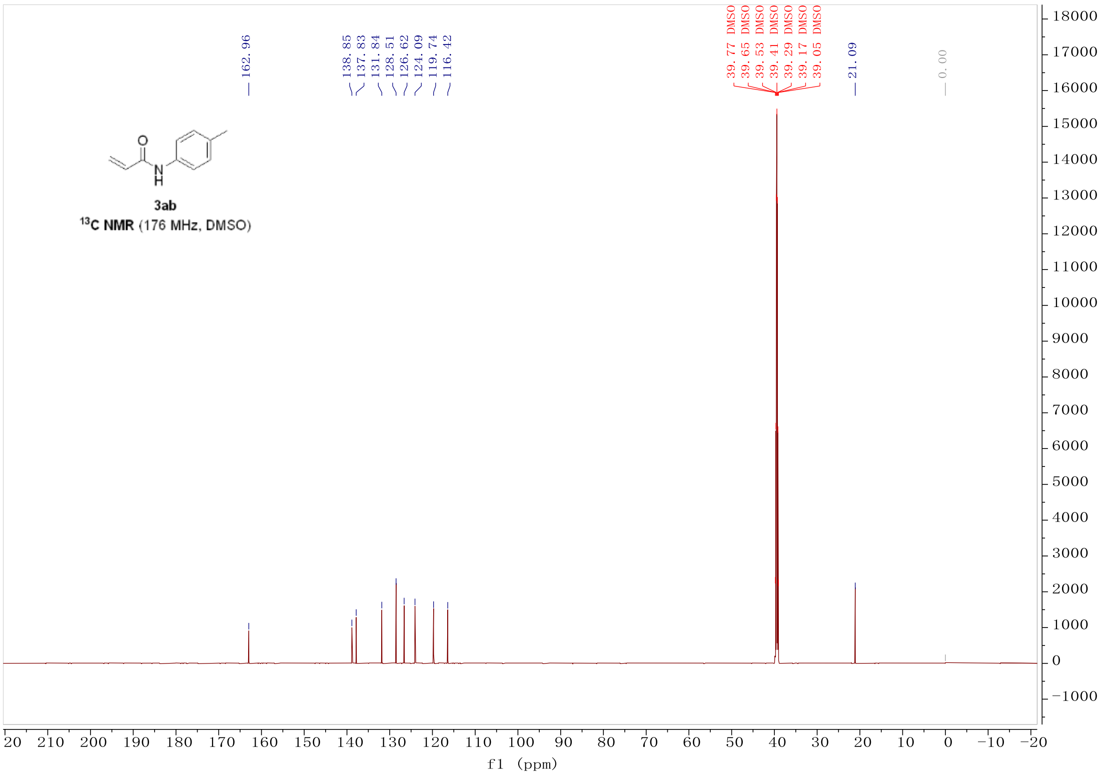
3ab
13C NMR (176 MHz, DMSO)
162.96
138.85 137.83 131.84 128.51 126.62 124.09 119.74 116.42
39.77 DMSO 39.65 DMSO 39.53 DMSO 39.41 DMSO 39.29 DMSO 39.17 DMSO 39.05 DMSO
21.09
0.00
f1 (ppm)

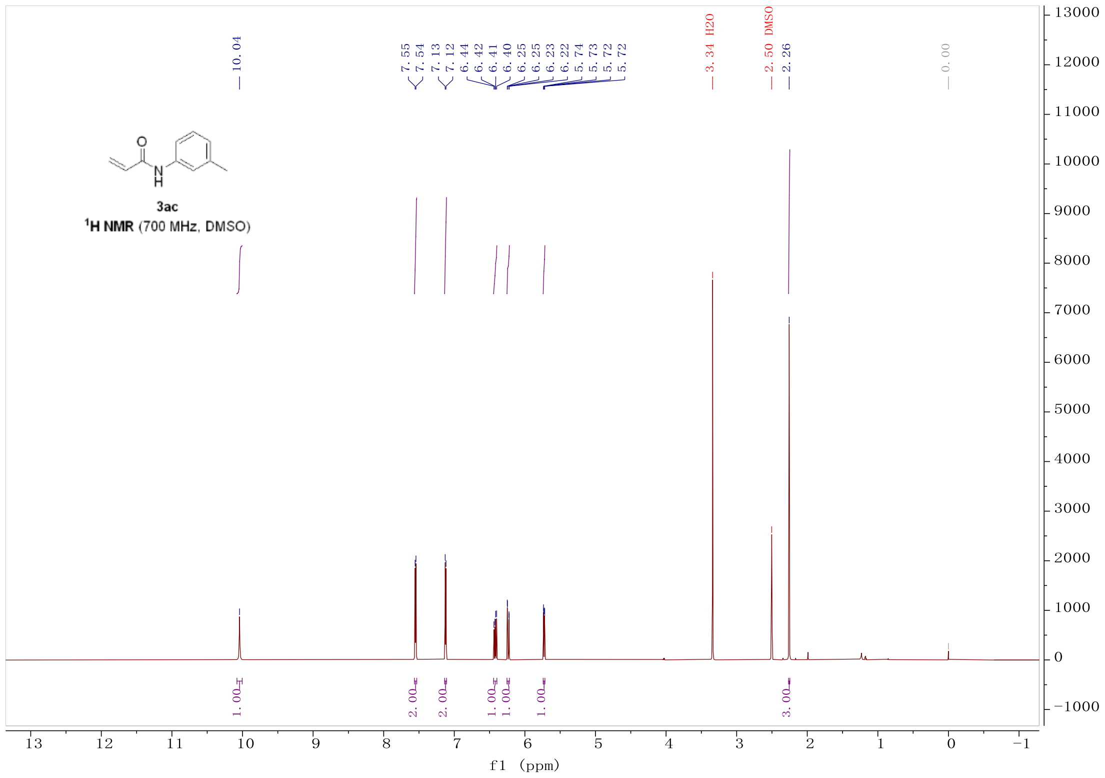

10.04
7.55
7.54
7.13
7.12
6.44
6.42
6.41
6.40
6.25
6.25
6.23
6.22
5.74
5.73
5.72
5.72
3.34 H2O
2.50 DMSO
2.26
0.00
3ac
1H NMR (700 MHz, DMSO)
1.00
2.00
2.00
1.00
1.00
1.00
3.00
f1 (ppm)

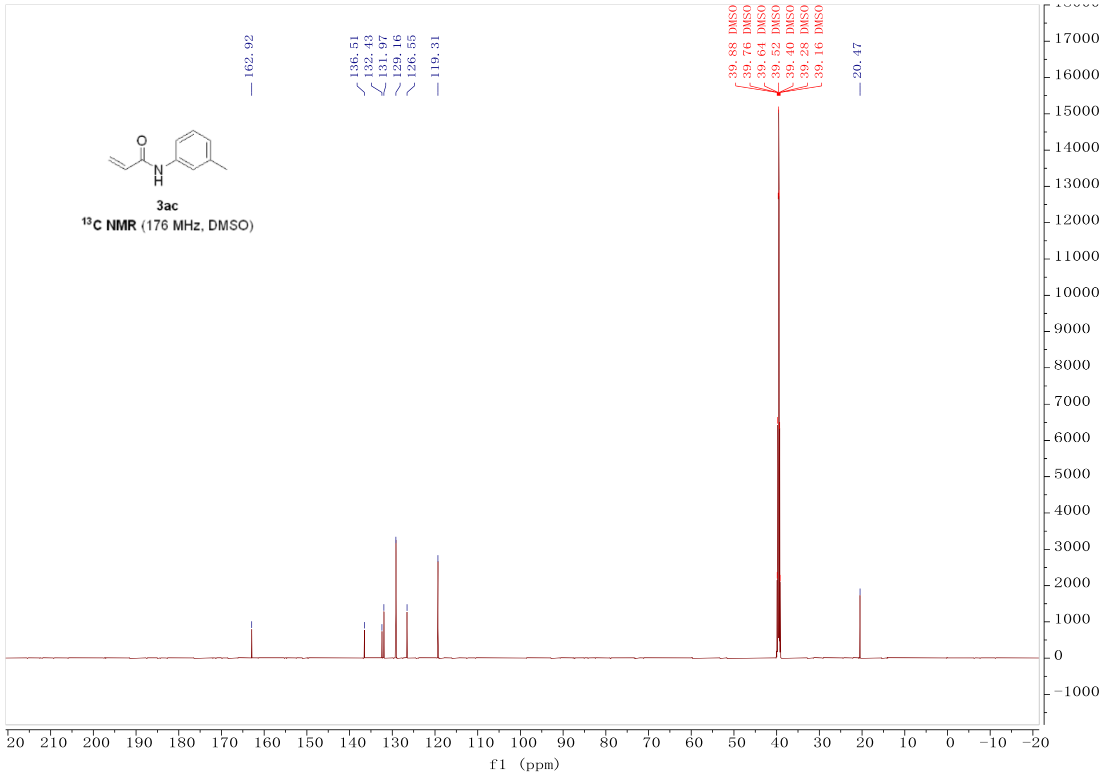

162.92
136.51
132.43
131.97
129.16
126.55
119.31
39.88 DMSO
39.76 DMSO
39.64 DMSO
39.52 DMSO
39.40 DMSO
39.28 DMSO
39.16 DMSO
20.47
3ac
13C NMR (176 MHz, DMSO)
f1 (ppm)

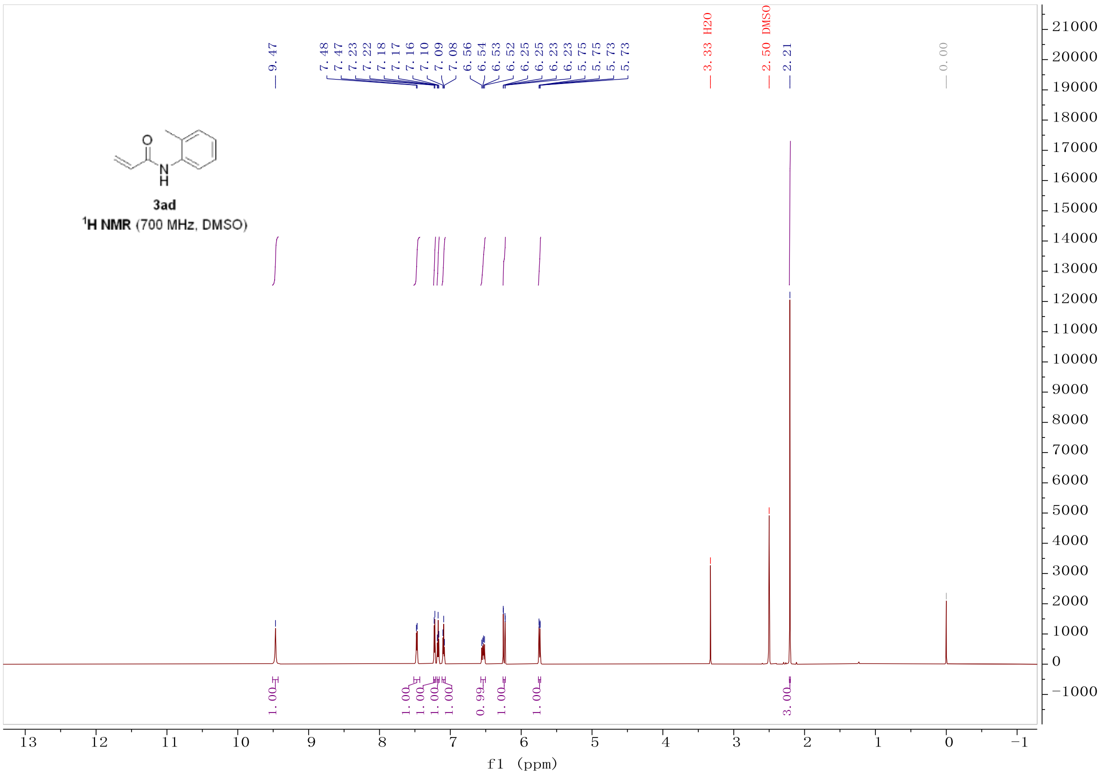
3ad
1H NMR (700 MHz, DMSO)
9.47
7.48
7.47
7.23
7.22
7.18
7.17
7.16
7.10
7.09
7.08
6.56
6.54
6.53
6.52
6.25
6.25
6.23
6.23
5.75
5.75
5.73
5.73
3.33 H2O
2.50 DMSO
2.21
0.00
1.00
1.00
1.00
1.00
1.00
0.99
1.00
1.00
3.00
f1 (ppm)

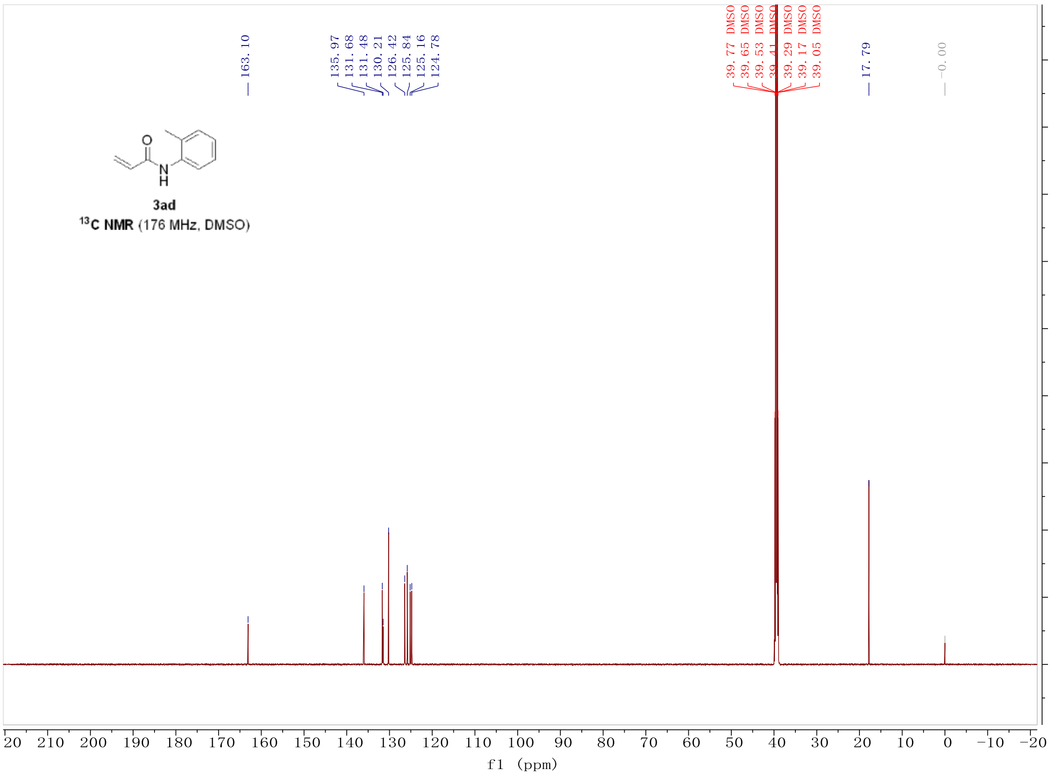
3ad
13C NMR (176 MHz, DMSO)
163.10
135.97
131.68
131.48
130.21
126.42
125.84
125.16
124.78
39.77 DMSO
39.65 DMSO
39.53 DMSO
39.41 DMSO
39.29 DMSO
39.17 DMSO
39.05 DMSO
17.79
-0.00
f1 (ppm)

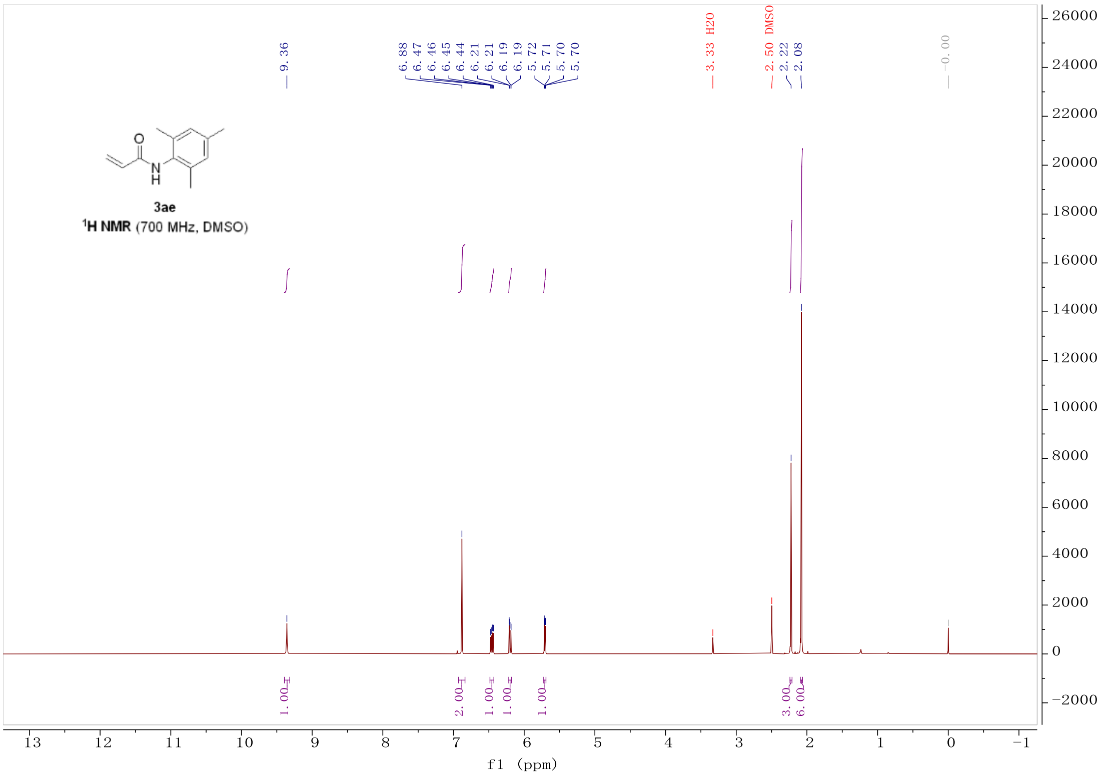
3ae
1H NMR (700 MHz, DMSO)
9.36
6.88
6.47
6.46
6.45
6.44
6.21
6.21
6.19
6.19
5.72
5.71
5.70
5.70
3.33 H2O
2.50 DMSO
2.22
2.08
-0.00
1.00
2.00
1.00
1.00
1.00
3.00
6.00
f1 (ppm)

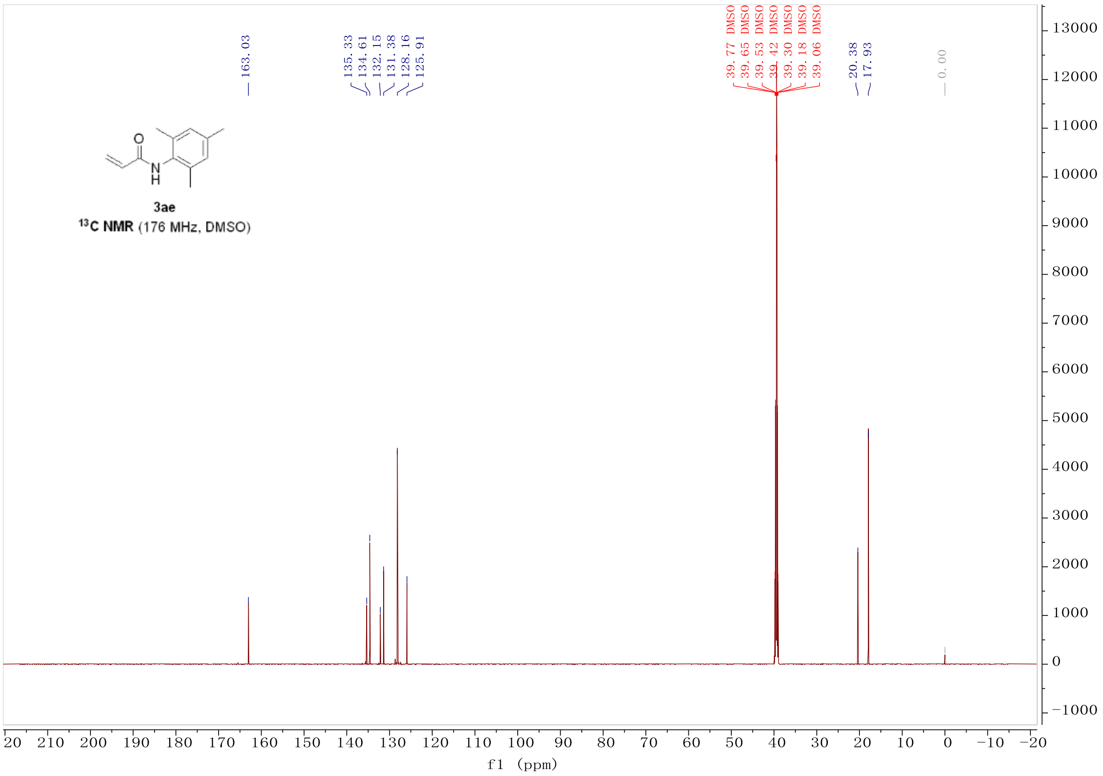
3ae
13C NMR (176 MHz, DMSO)
163.03
135.33
134.61
132.15
131.38
128.16
125.91
39.77 DMSO
39.65 DMSO
39.53 DMSO
39.42 DMSO
39.30 DMSO
39.18 DMSO
39.06 DMSO
20.38
17.93
0.00
f1 (ppm)

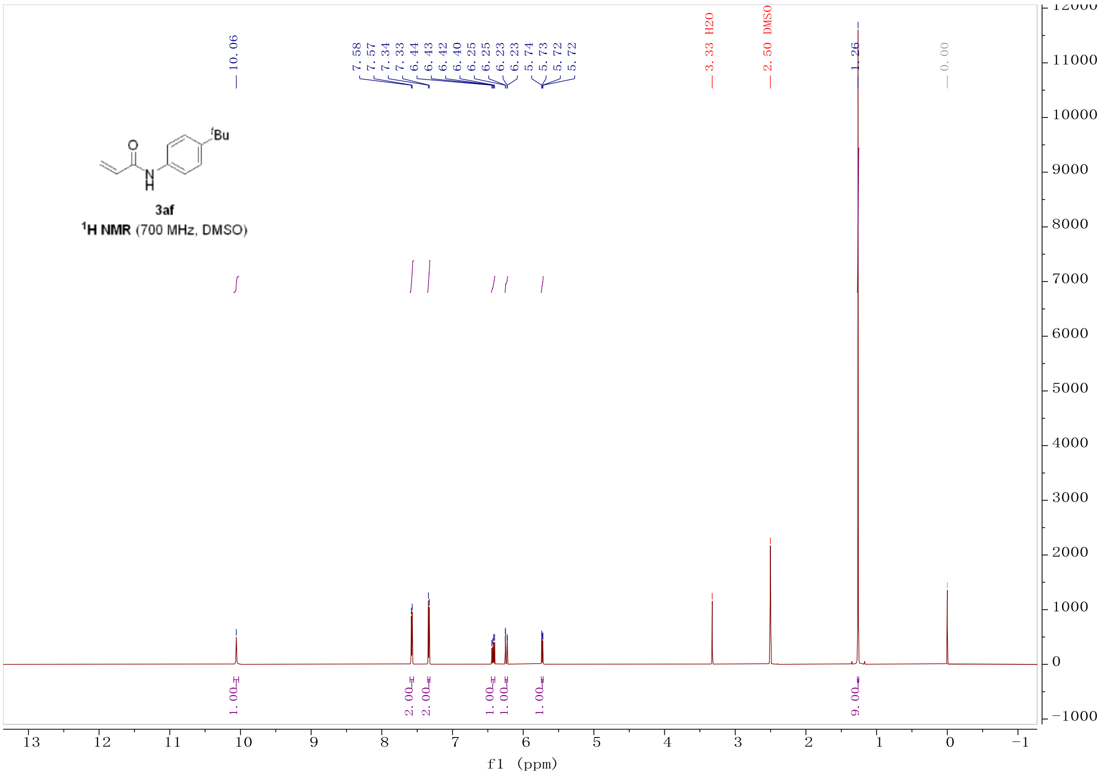

10.06
7.58
7.57
7.34
7.33
6.44
6.43
6.42
6.40
6.25
6.23
6.23
5.74
5.73
5.72
5.72
3.33 H2O
2.50 DMSO
1.26
0.00
tBu
3af
1H NMR (700 MHz, DMSO)
1.00
2.00
2.00
1.00
1.00
1.00
9.00
f1 (ppm)

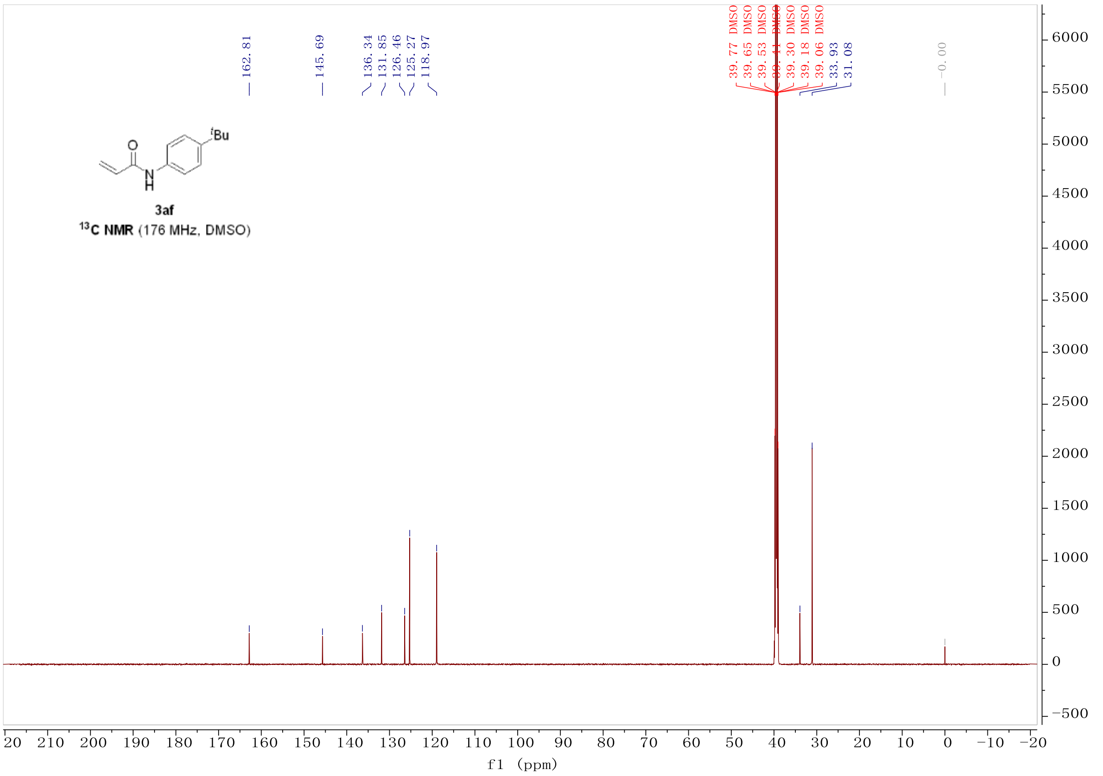

162.81
145.69
136.34
131.85
126.46
125.27
118.97
39.77 DMSO
39.65 DMSO
39.53 DMSO
39.30 DMSO
39.18 DMSO
39.06 DMSO
33.93
31.08
0.00
tBu
3af
13C NMR (176 MHz, DMSO)
f1 (ppm)

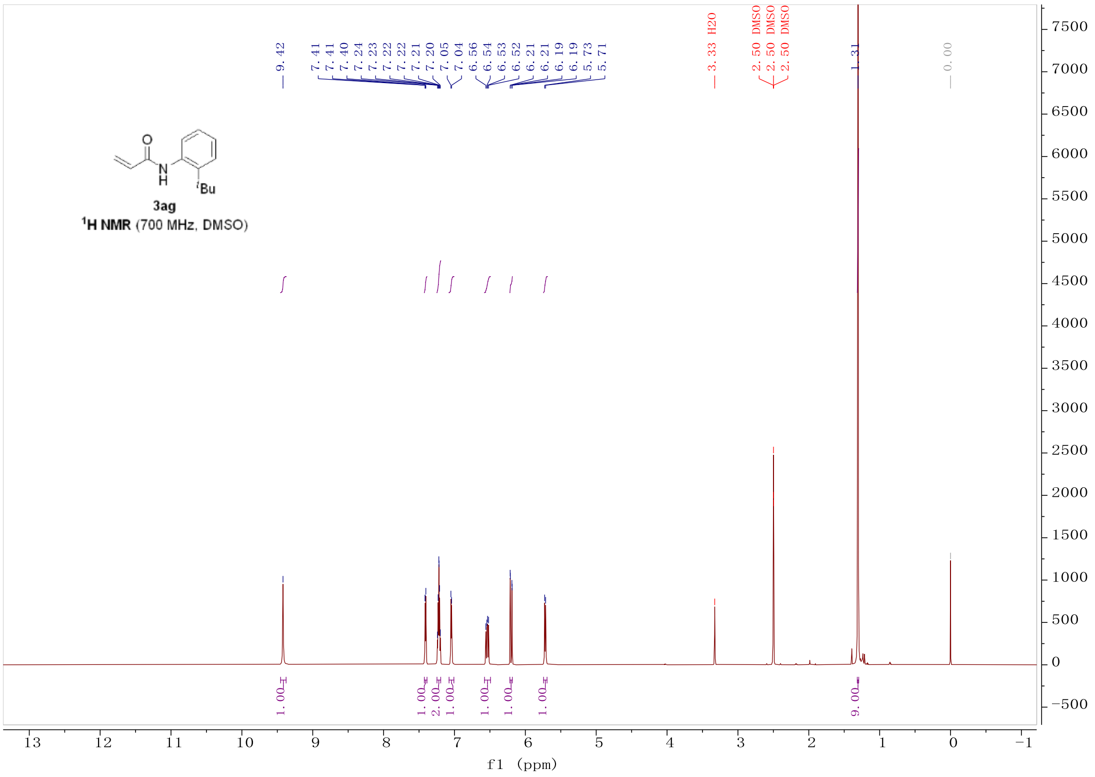
3ag
¹H NMR (700 MHz, DMSO)
f1 (ppm)

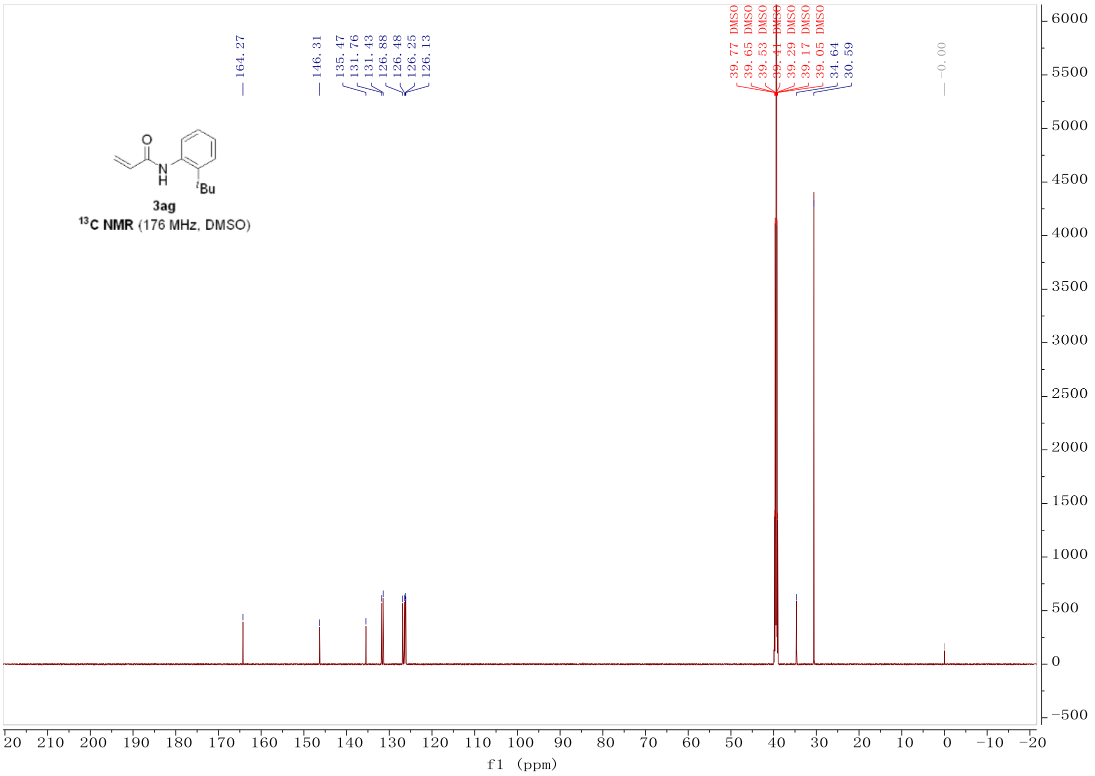
3ag
¹³C NMR (176 MHz, DMSO)
f1 (ppm)

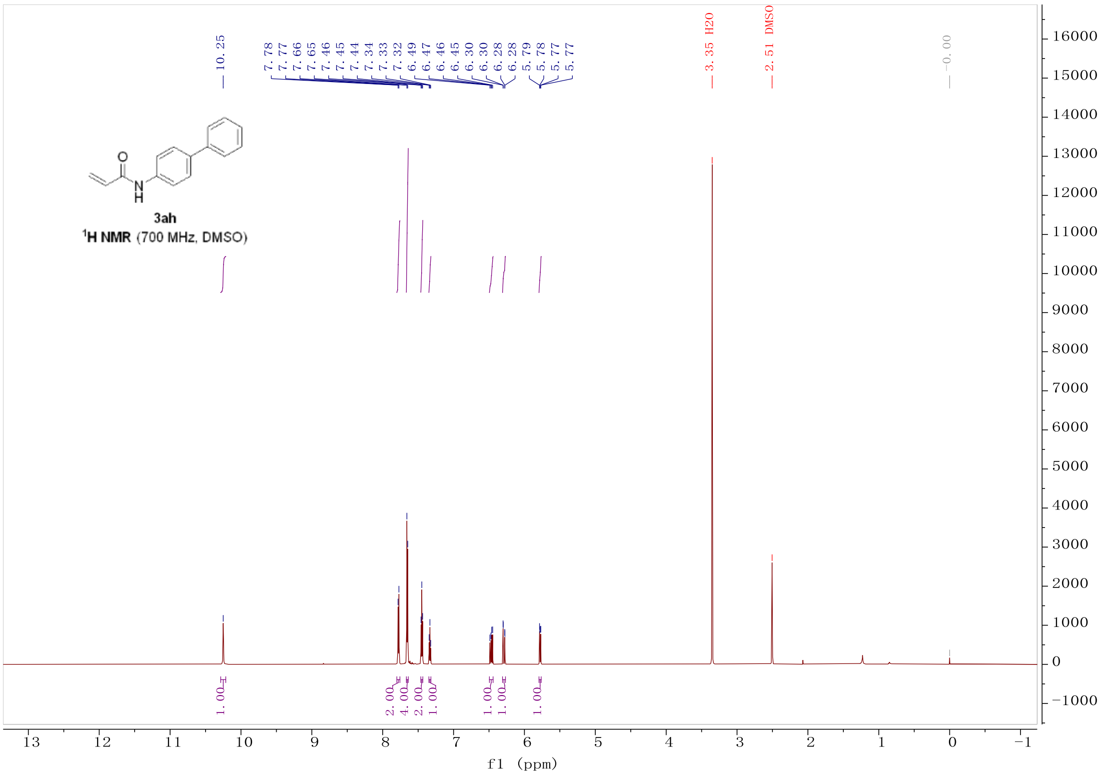
3ah
1H NMR (700 MHz, DMSO)
10.25
7.78 7.77 7.66 7.65 7.46 7.45 7.44 7.34 7.33 7.32 6.49 6.47 6.46 6.45 6.30 6.30 6.28 6.28 5.79 5.78 5.77 5.77
3.35 H2O
2.51 DMSO
0.00
1.00 2.00 4.00 2.00 1.00 1.00 1.00 1.00
f1 (ppm)

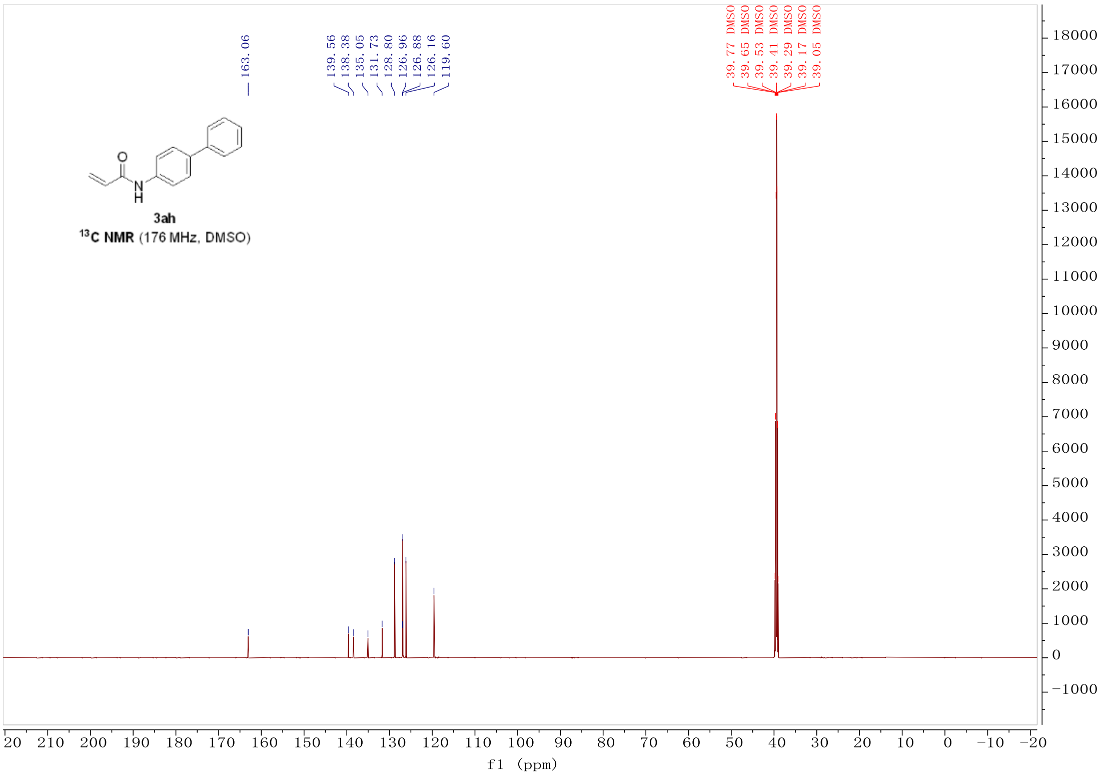
3ah
13C NMR (176 MHz, DMSO)
163.06
139.56 138.38 135.05 131.73 128.80 126.96 126.88 126.16 119.60
39.77 DMSO 39.65 DMSO 39.53 DMSO 39.41 DMSO 39.29 DMSO 39.17 DMSO 39.05 DMSO
f1 (ppm)

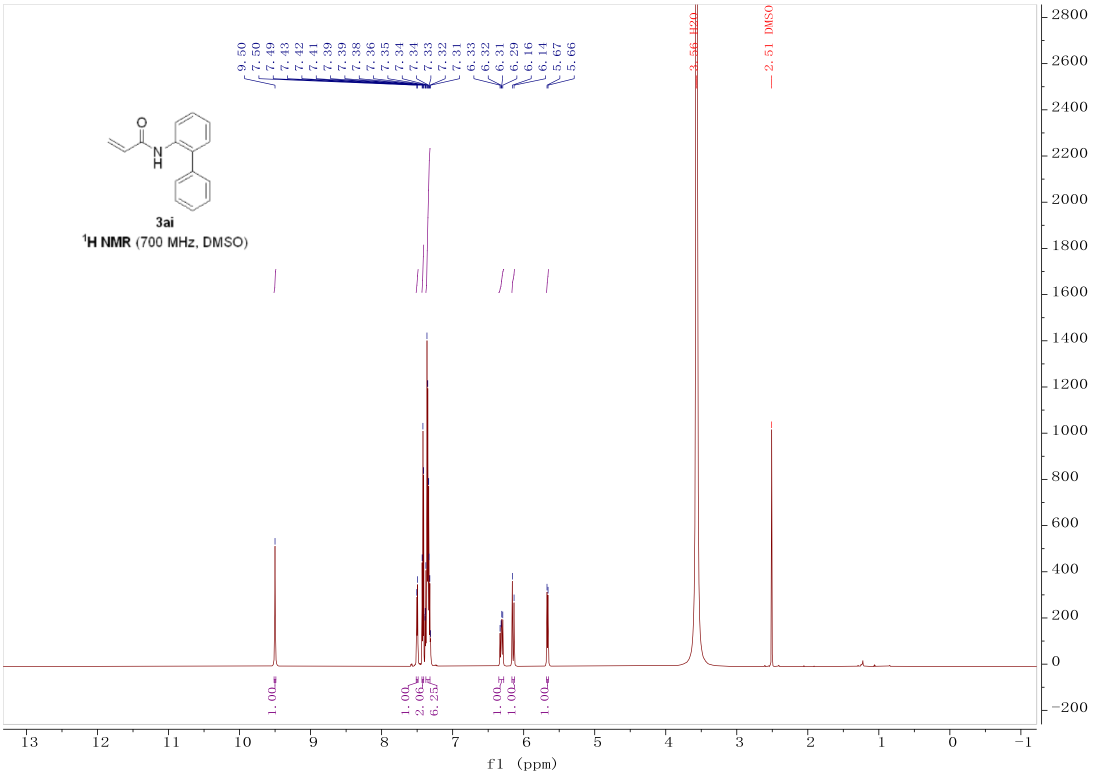
9.50 7.50 7.49 7.43 7.42 7.41 7.39 7.39 7.38 7.36 7.35 7.34 7.34 7.33 7.32 7.31 6.33 6.32 6.31 6.29 6.16 6.14 5.67 5.66
3.56 H2O
2.51 DMSO
O
N
H
3ai
1H NMR (700 MHz, DMSO)
1.00
1.00
2.06
6.25
1.00
1.00
1.00
f1 (ppm)

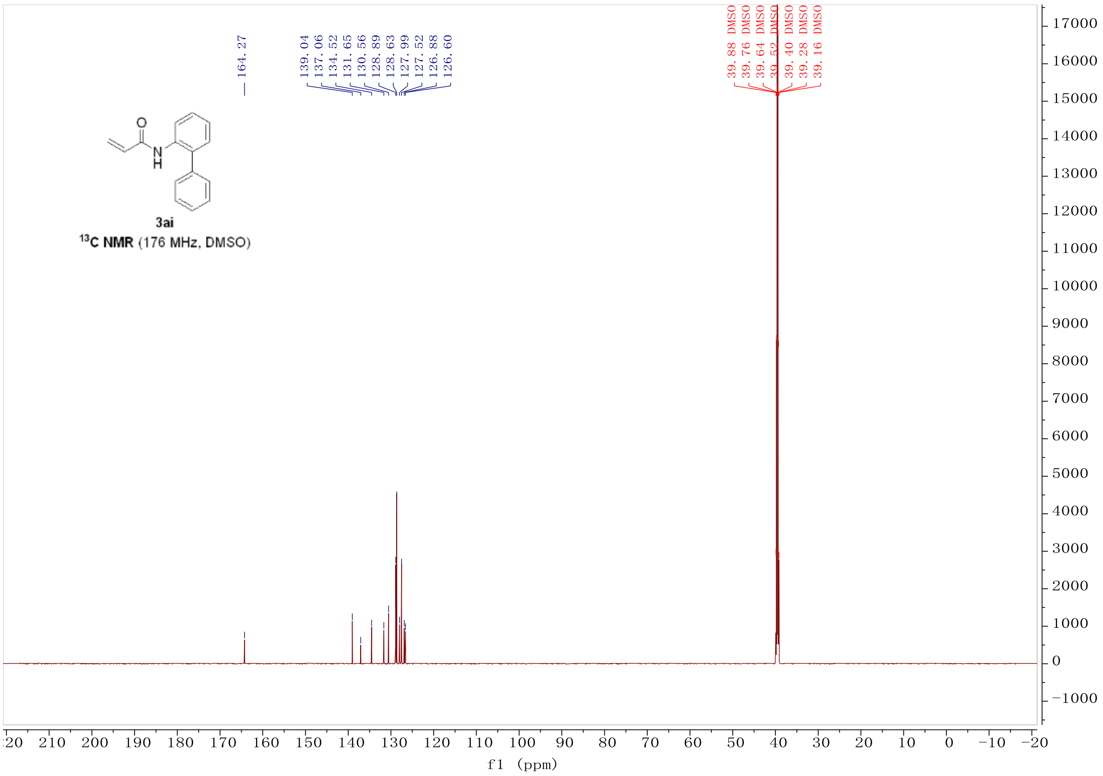
164.27
139.04 137.06 134.52 131.65 130.56 128.89 128.63 127.99 127.52 126.88 126.60
39.88 DMSO 39.76 DMSO 39.64 DMSO 39.52 DMSO 39.40 DMSO 39.28 DMSO 39.16 DMSO
O
N
H
3ai
13C NMR (176 MHz, DMSO)
f1 (ppm)

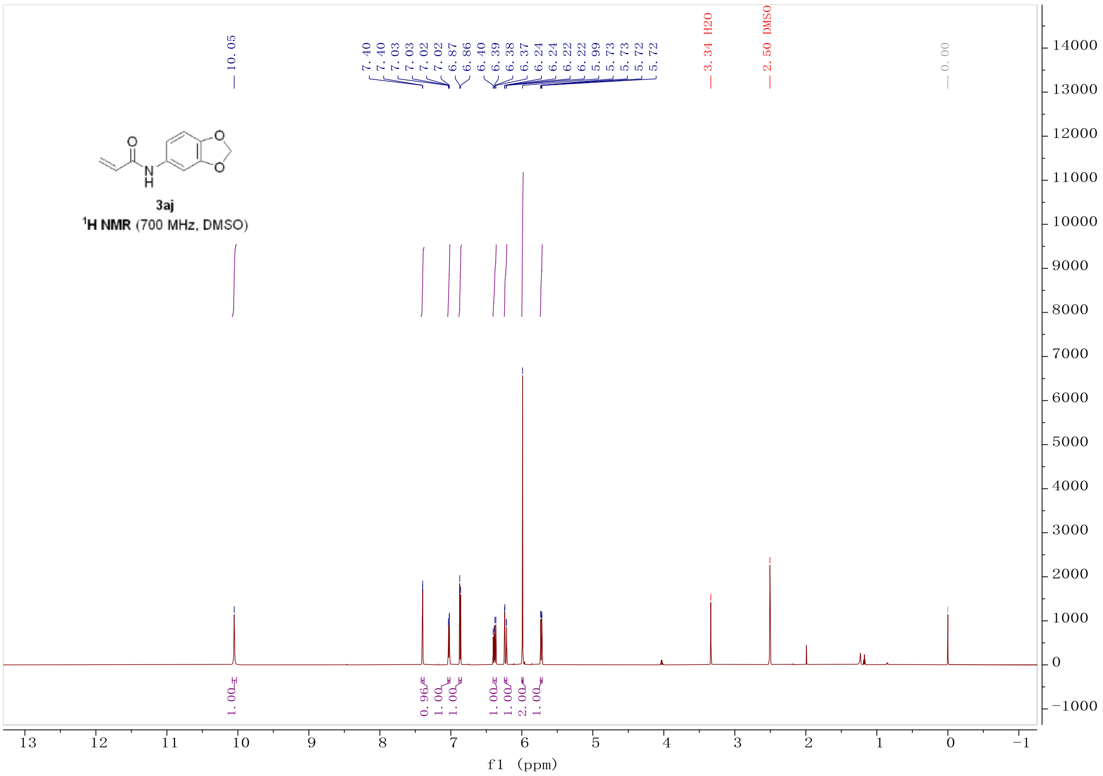
3aj
¹H NMR (700 MHz, DMSO)
10.05
7.40
7.40
7.03
7.03
7.02
7.02
6.87
6.86
6.40
6.39
6.38
6.37
6.24
6.24
6.22
6.22
5.99
5.73
5.73
5.72
5.72
3.34 H2O
2.50 DMSO
0.00
1.00
0.96
1.00
1.00
1.00
1.00
2.00
1.00
f1 (ppm)

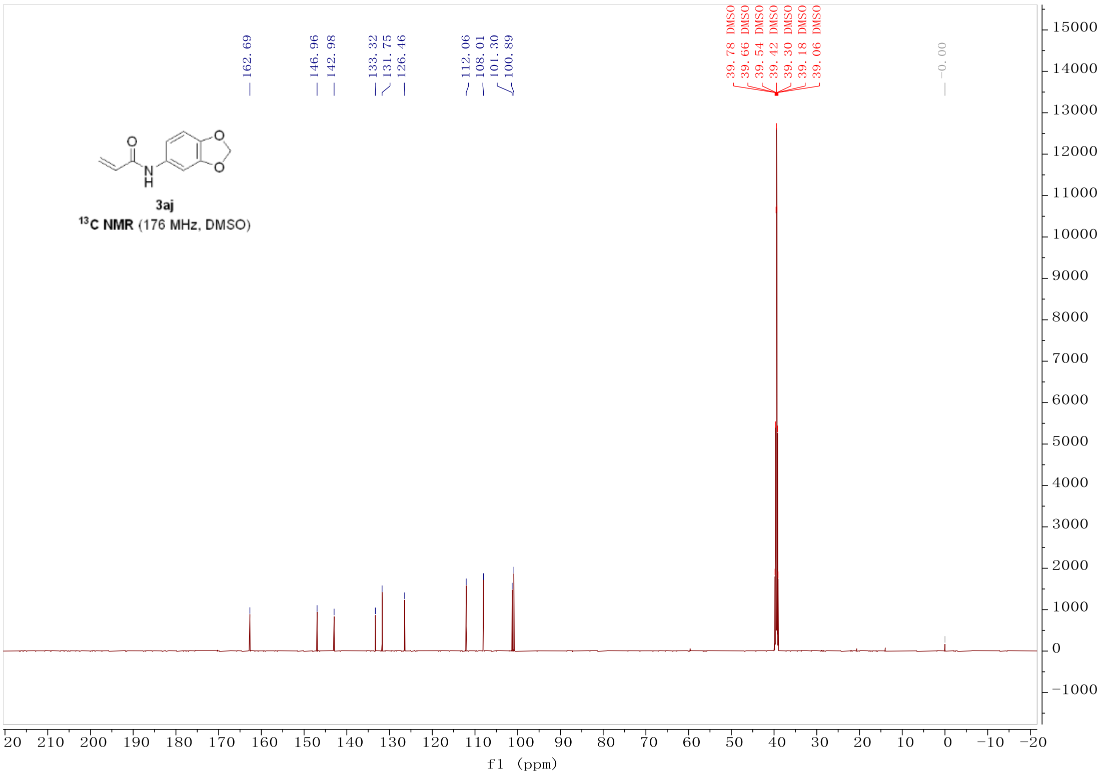
3aj
¹³C NMR (176 MHz, DMSO)
162.69
146.96
142.98
133.32
131.75
126.46
112.06
108.01
101.30
100.89
39.78 DMSO
39.66 DMSO
39.54 DMSO
39.42 DMSO
39.30 DMSO
39.18 DMSO
39.06 DMSO
-0.00
f1 (ppm)

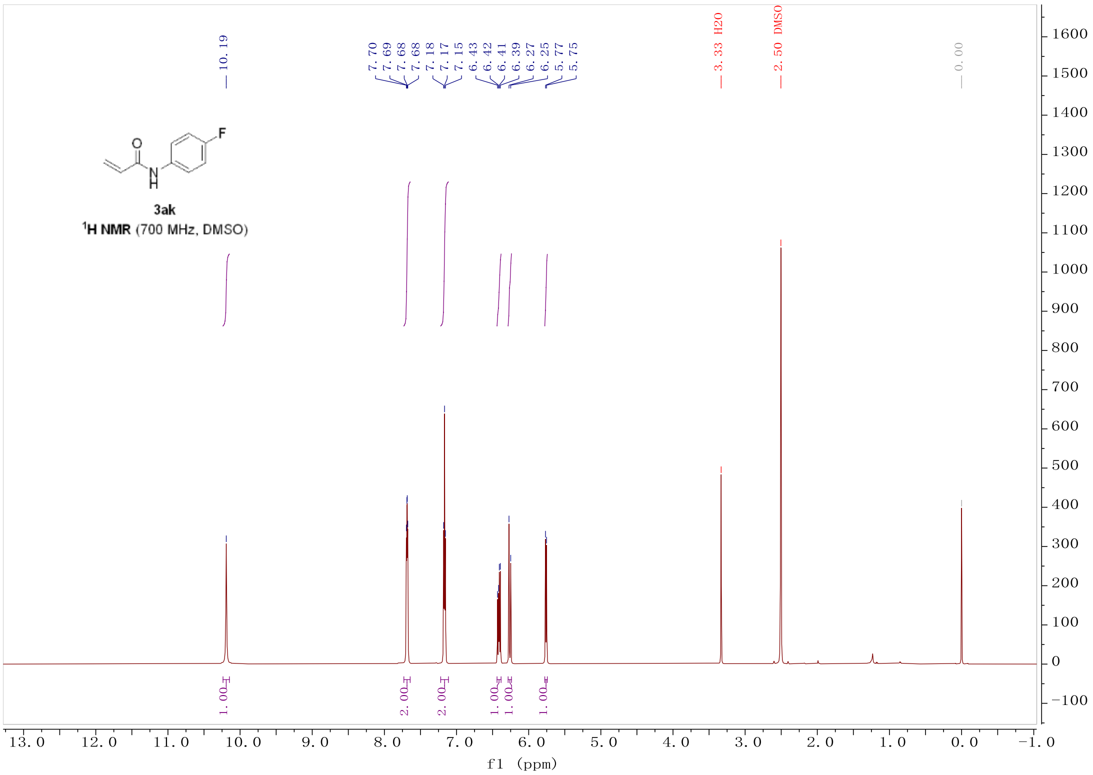
10.19
7.70
7.69
7.68
7.68
7.18
7.17
7.15
6.43
6.42
6.41
6.39
6.27
6.25
5.77
5.75
3.33 H2O
2.50 DMSO
0.00
3ak
1H NMR (700 MHz, DMSO)
1.00
2.00
2.00
1.00
1.00
1.00
f1 (ppm)

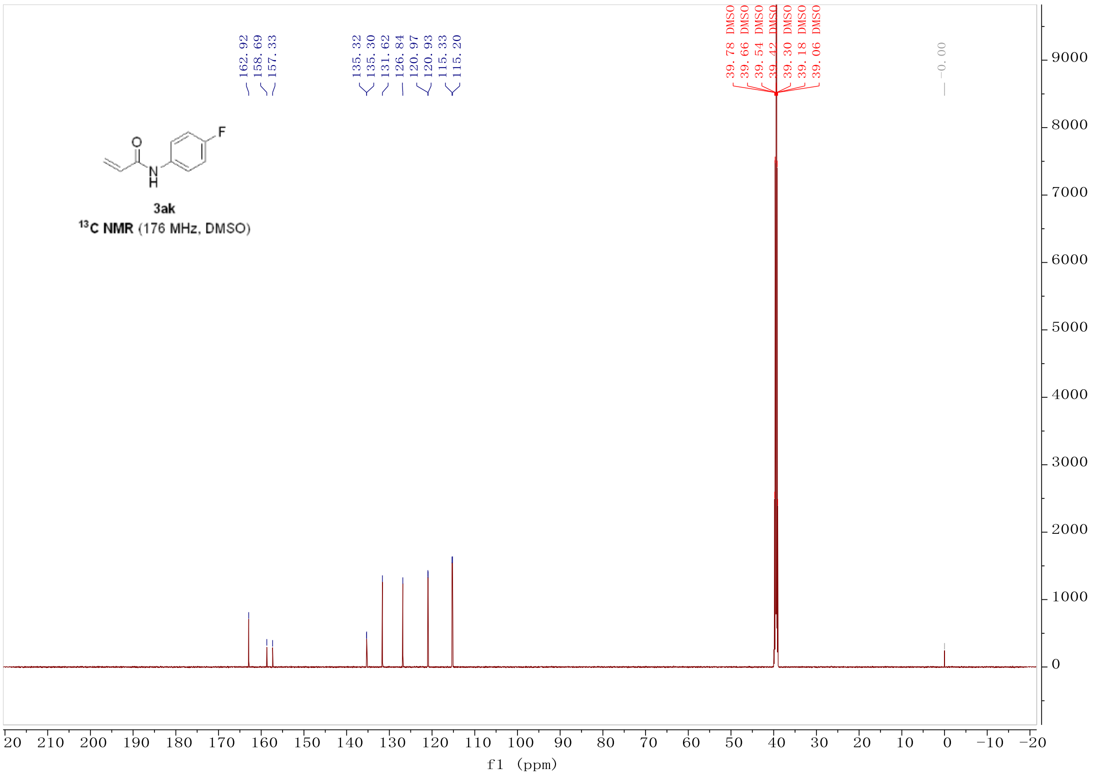
162.92
158.69
157.33
135.32
135.30
131.62
126.84
120.97
120.93
115.33
115.20
39.78 DMSO
39.66 DMSO
39.54 DMSO
39.42 DMSO
39.30 DMSO
39.18 DMSO
39.06 DMSO
-0.00
3ak
13C NMR (176 MHz, DMSO)
f1 (ppm)

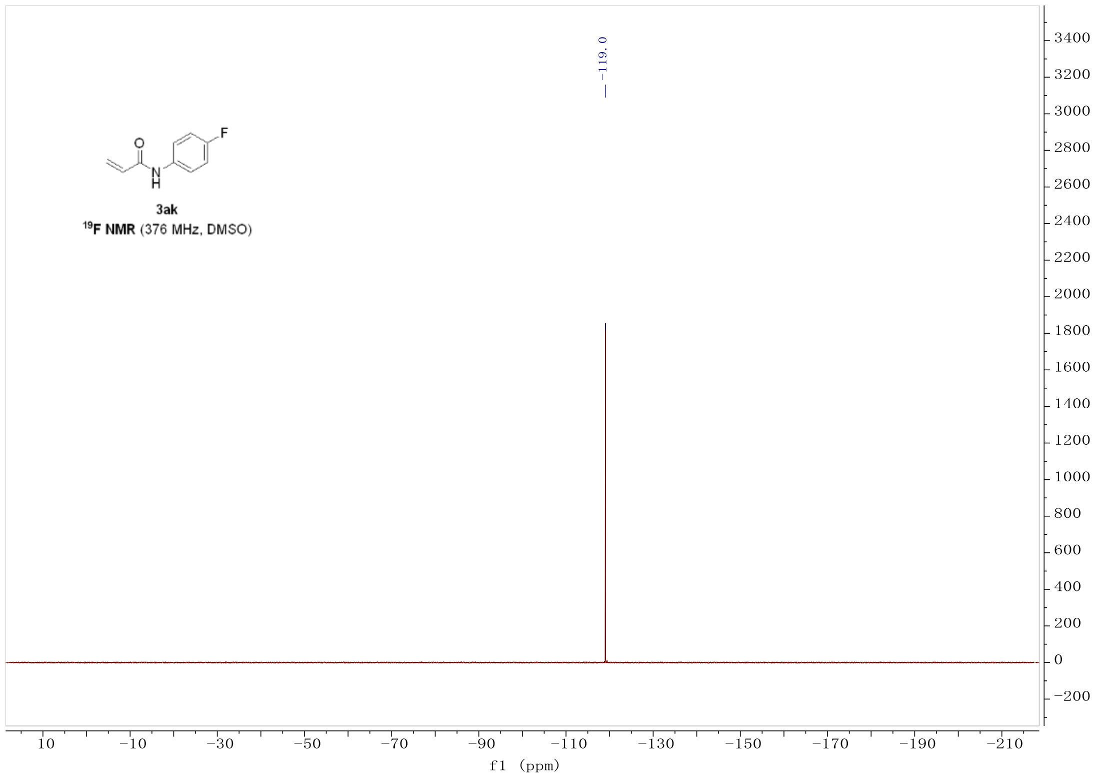

−119.0
3ak
19F NMR (376 MHz, DMSO)
f1 (ppm)

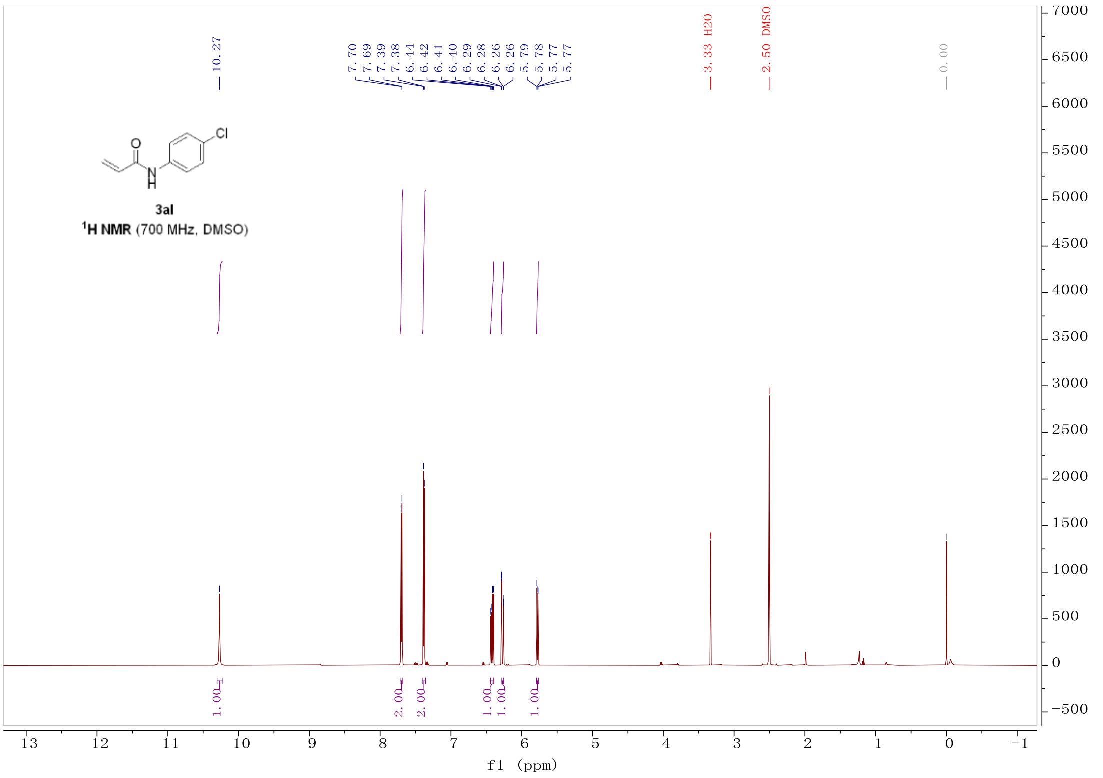
10.27
7.70 7.69 7.39 7.38 6.44 6.42 6.41 6.40 6.29 6.28 6.26 6.26 5.79 5.78 5.77 5.77
3.33 H2O
2.50 DMSO
0.00
3al
1H NMR (700 MHz, DMSO)
1.00 2.00 2.00 1.00 1.00 1.00
f1 (ppm)

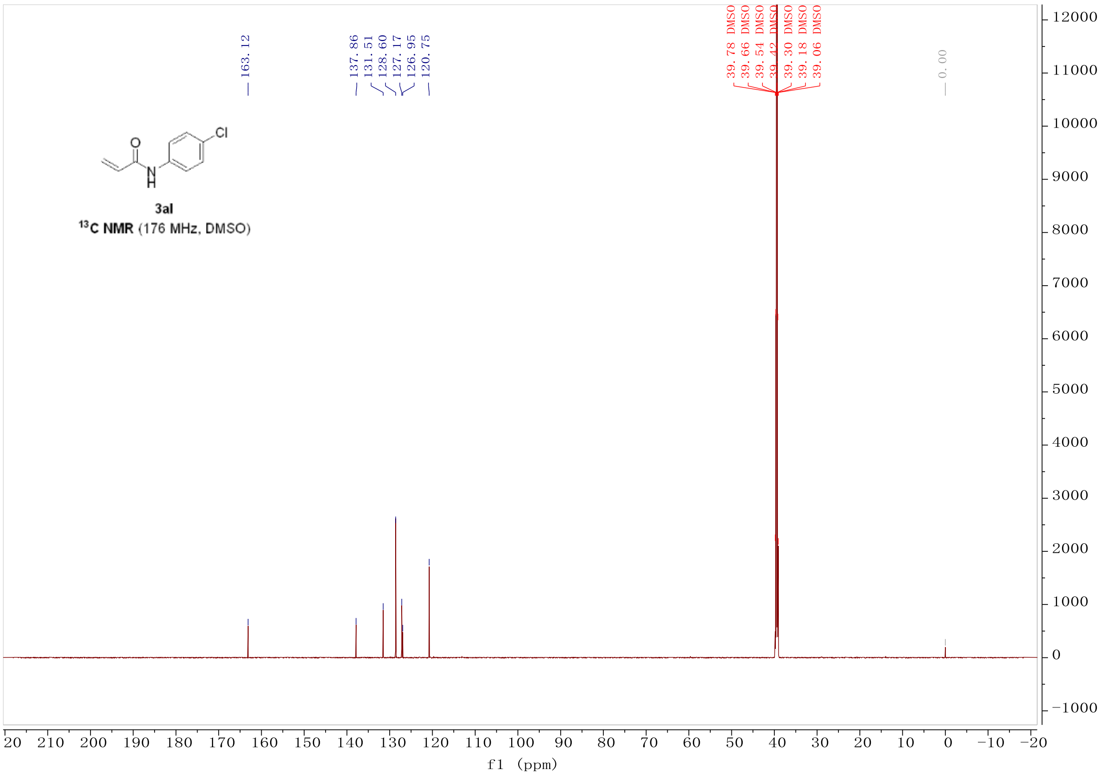
163.12
137.86 131.51 128.60 127.17 126.95 120.75
39.78 DMSO 39.66 DMSO 39.54 DMSO 39.42 DMSO 39.30 DMSO 39.18 DMSO 39.06 DMSO
0.00
3al
13C NMR (176 MHz, DMSO)
f1 (ppm)

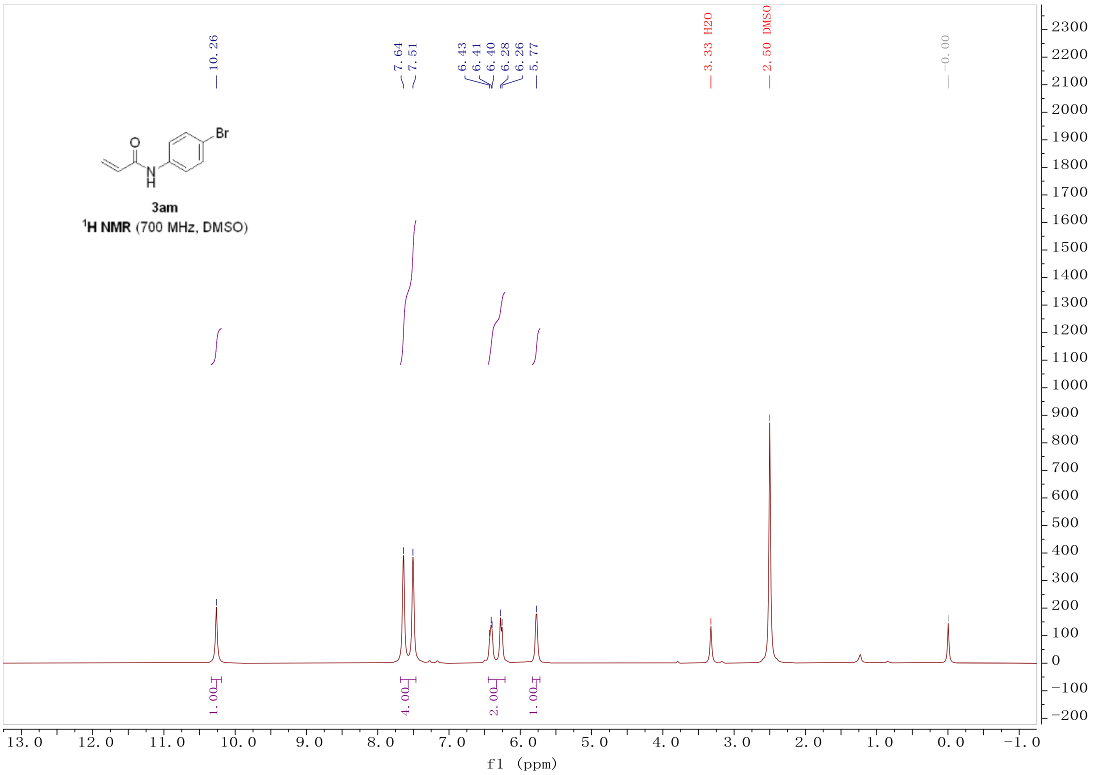

10.26
7.64
7.51
6.43
6.41
6.40
6.28
6.26
5.77
3.33 H2O
2.50 DMSO
-0.00
Br
O
N
H
3am
1H NMR (700 MHz, DMSO)
1.00
4.00
2.00
1.00
f1 (ppm)

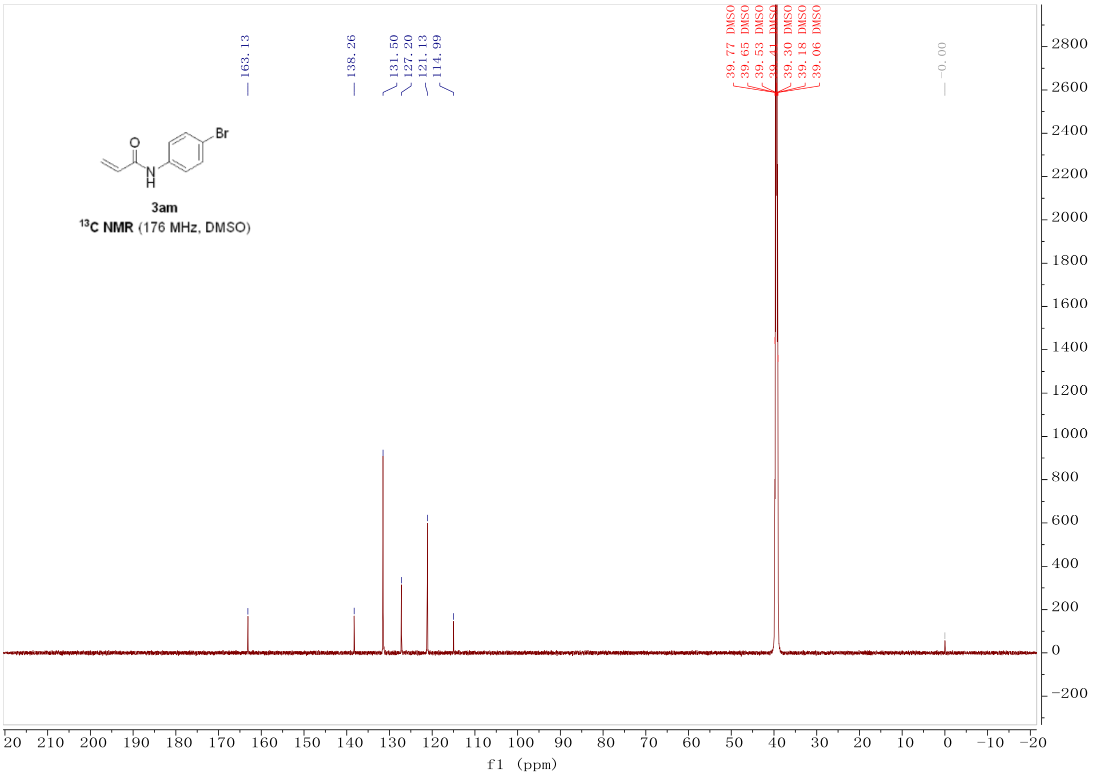

163.13
138.26
131.50
127.20
121.13
114.99
39.77 DMSO
39.65 DMSO
39.53 DMSO
39.41 DMSO
39.30 DMSO
39.18 DMSO
39.06 DMSO
-0.00
Br
O
N
H
3am
13C NMR (176 MHz, DMSO)
f1 (ppm)

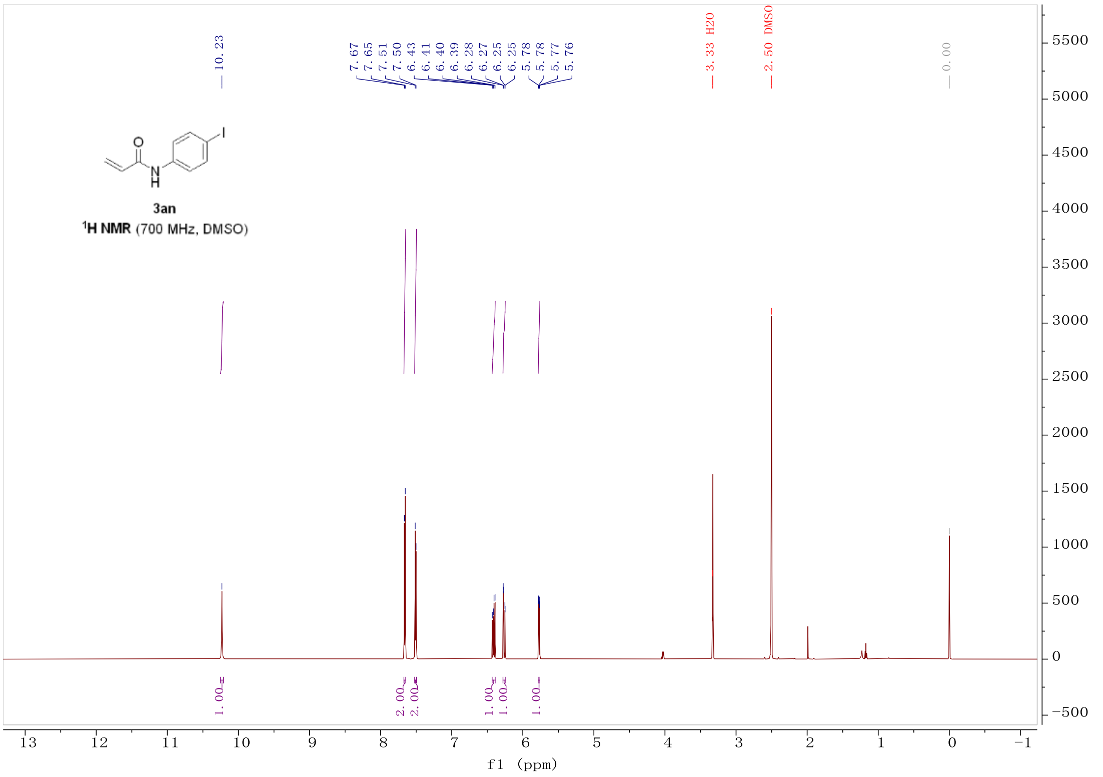
3an
1H NMR (700 MHz, DMSO)
10.23
7.67
7.65
7.51
7.50
6.43
6.41
6.40
6.39
6.28
6.27
6.25
6.25
5.78
5.77
5.76
3.33 H2O
2.50 DMSO
0.00
1.00
2.00
2.00
1.00
1.00
1.00
f1 (ppm)

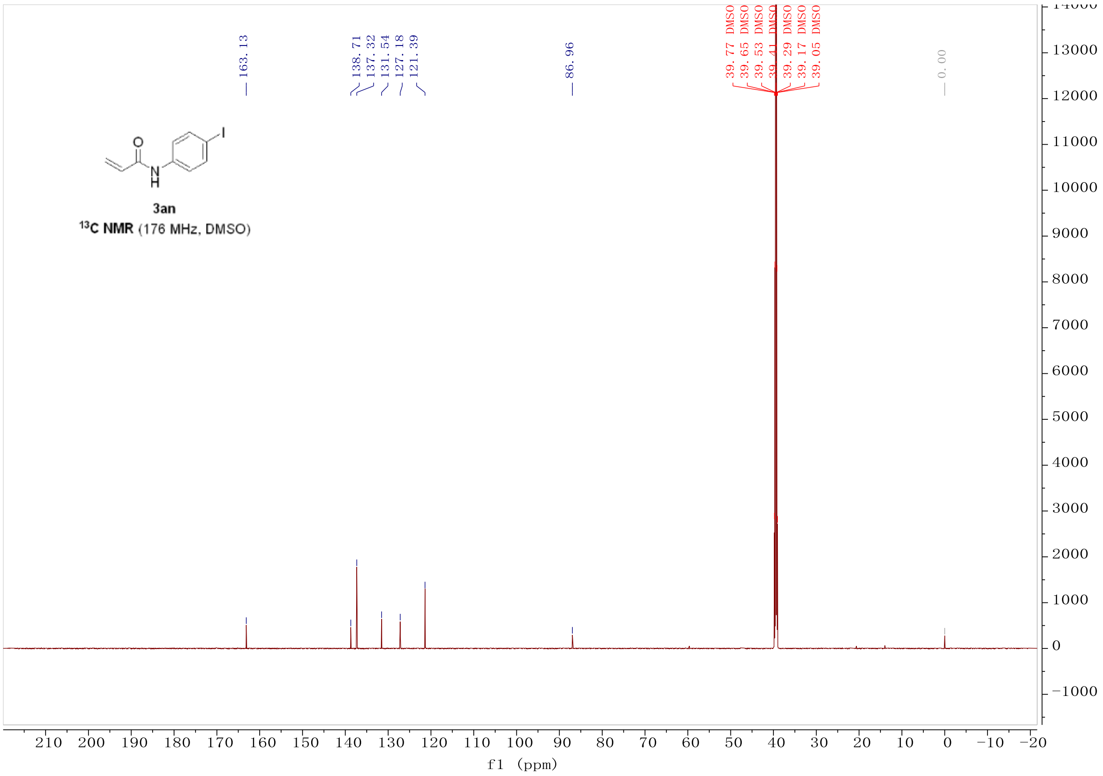
3an
13C NMR (176 MHz, DMSO)
163.13
138.71
137.32
131.54
127.18
121.39
86.96
39.77 DMSO
39.65 DMSO
39.53 DMSO
39.41 DMSO
39.29 DMSO
39.17 DMSO
39.05 DMSO
0.00
f1 (ppm)

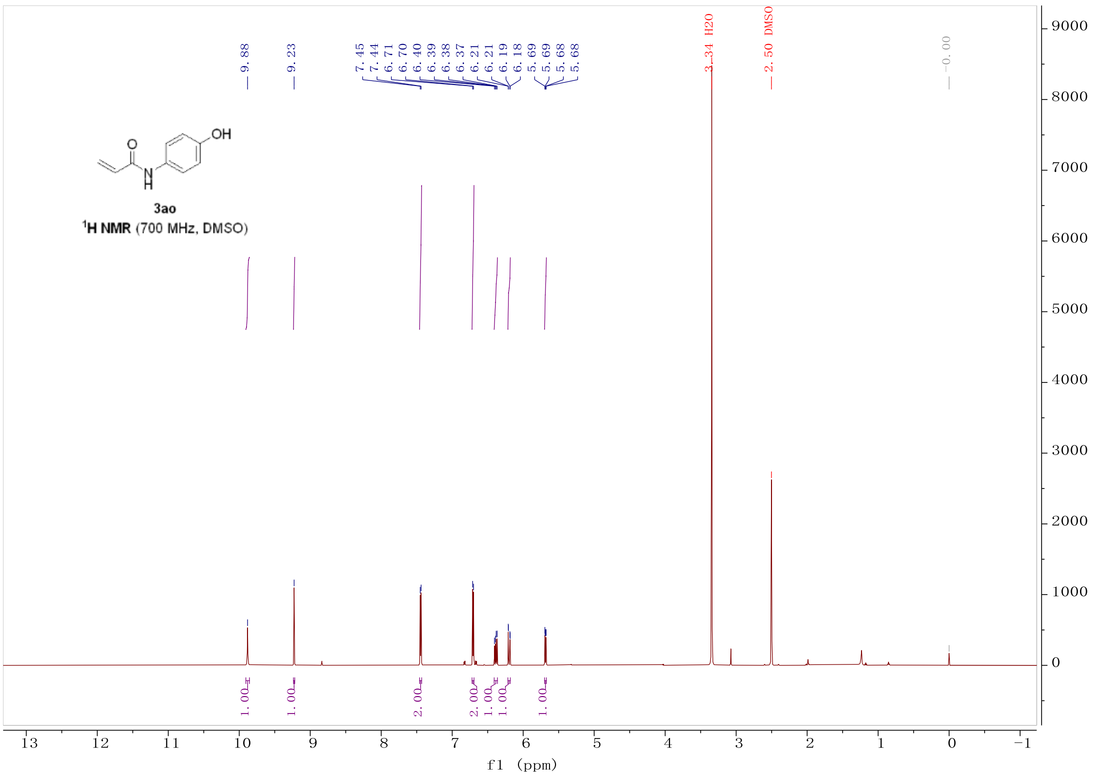
OH
O
N
H
3ao
1H NMR (700 MHz, DMSO)
9.88
9.23
7.45
7.44
6.71
6.70
6.40
6.39
6.38
6.37
6.21
6.21
6.19
6.18
5.69
5.69
5.68
5.68
3.34 H2O
2.50 DMSO
0.00
1.00
1.00
2.00
2.00
1.00
1.00
1.00
f1 (ppm)

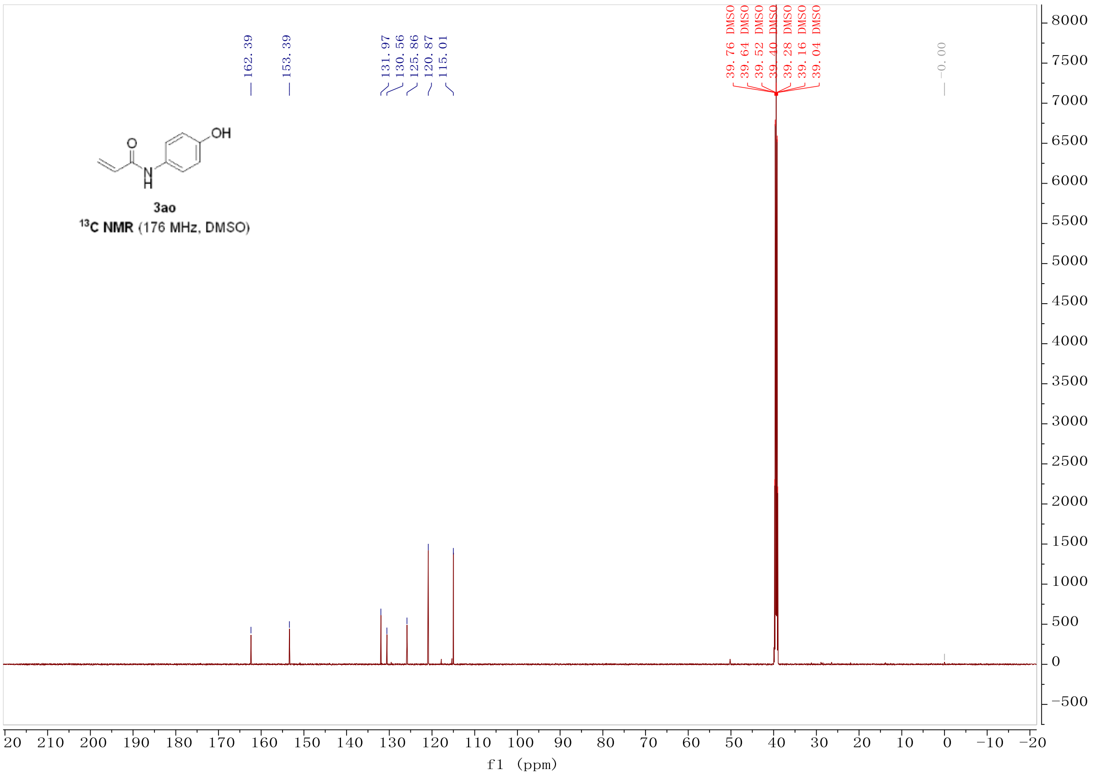
OH
O
N
H
3ao
13C NMR (176 MHz, DMSO)
162.39
153.39
131.97
130.56
125.86
120.87
115.01
39.76 DMSO
39.64 DMSO
39.52 DMSO
39.40 DMSO
39.28 DMSO
39.16 DMSO
39.04 DMSO
0.00
f1 (ppm)

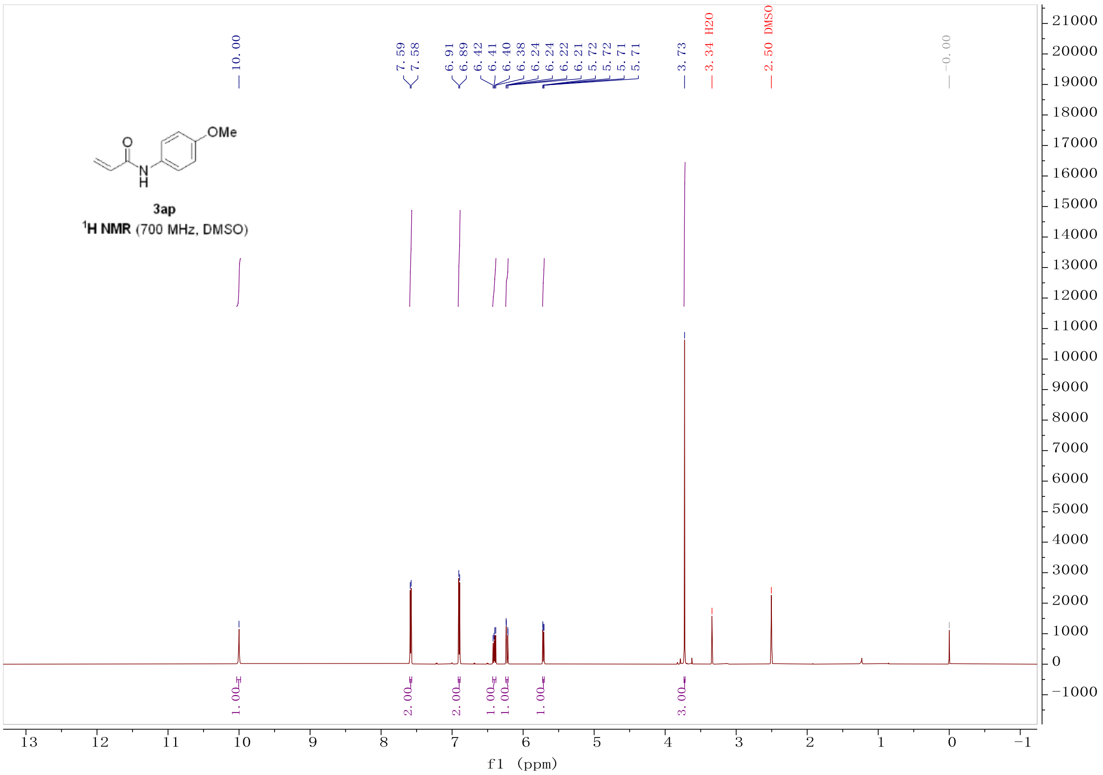
10.00
7.59
7.58
6.91
6.89
6.42
6.41
6.40
6.38
6.24
6.24
6.22
6.21
5.72
5.72
5.71
5.71
3.73
3.34 H2O
2.50 DMSO
-0.00
OMe
O
N
H
3ap
1H NMR (700 MHz, DMSO)
1.00
2.00
2.00
1.00
1.00
1.00
3.00
f1 (ppm)

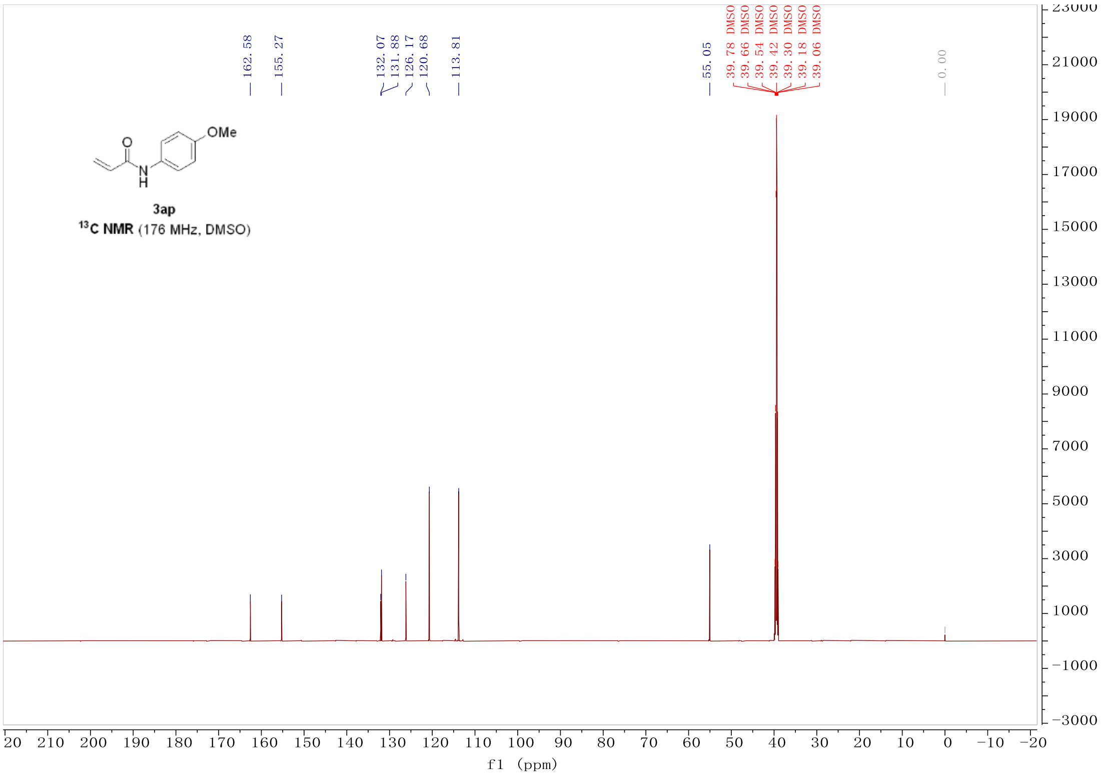
162.58
155.27
132.07
131.88
126.17
120.68
113.81
55.05
39.78 DMSO
39.66 DMSO
39.54 DMSO
39.42 DMSO
39.30 DMSO
39.18 DMSO
39.06 DMSO
0.00
OMe
O
N
H
3ap
13C NMR (176 MHz, DMSO)
f1 (ppm)

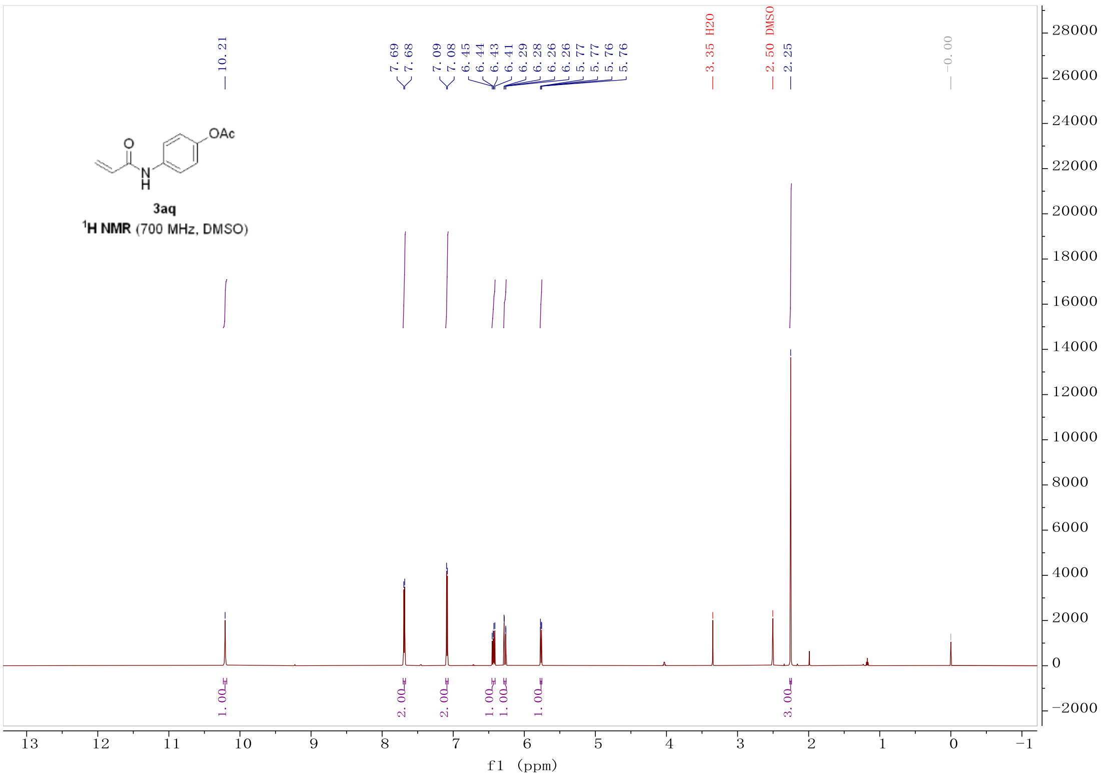
3aq
¹H NMR (700 MHz, DMSO)
10.21
7.69
7.68
7.09
7.08
6.45
6.44
6.43
6.41
6.29
6.28
6.26
6.26
5.77
5.76
5.76
3.35 H2O
2.50 DMSO
2.25
0.00
1.00
2.00
2.00
1.00
1.00
1.00
3.00
f1 (ppm)

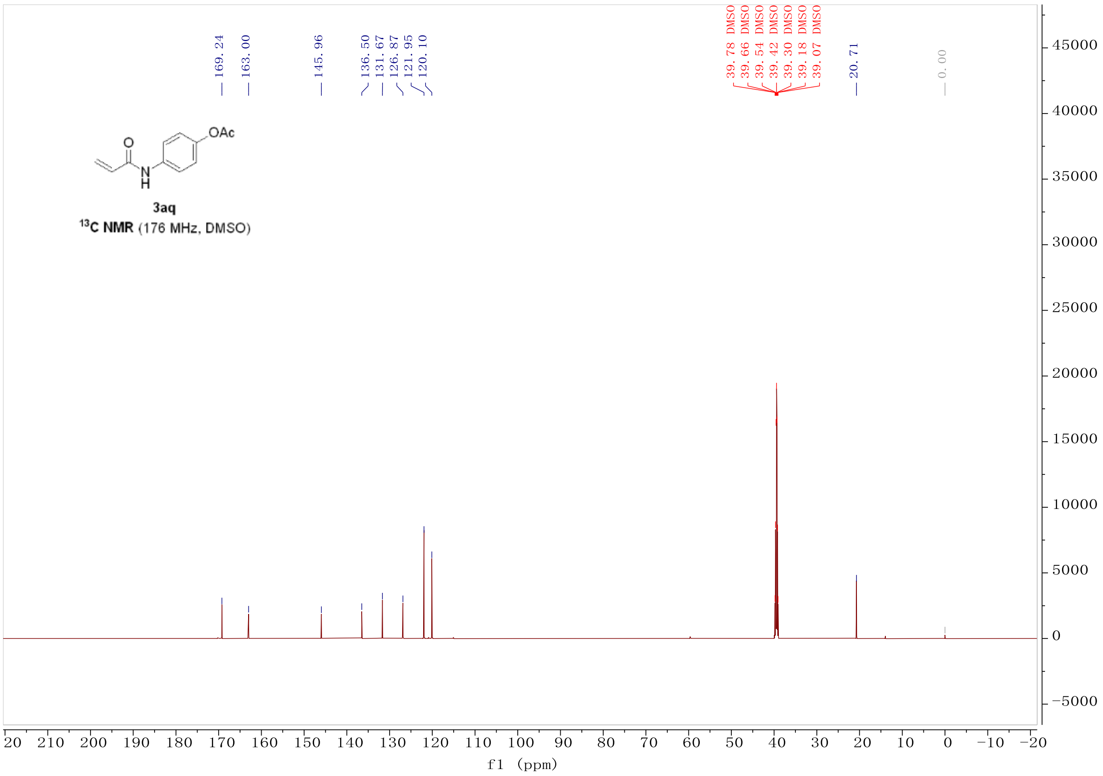
3aq
¹³C NMR (176 MHz, DMSO)
169.24
163.00
145.96
136.50
131.67
126.87
121.95
120.10
39.78 DMSO
39.66 DMSO
39.54 DMSO
39.42 DMSO
39.30 DMSO
39.18 DMSO
39.07 DMSO
20.71
0.00
f1 (ppm)

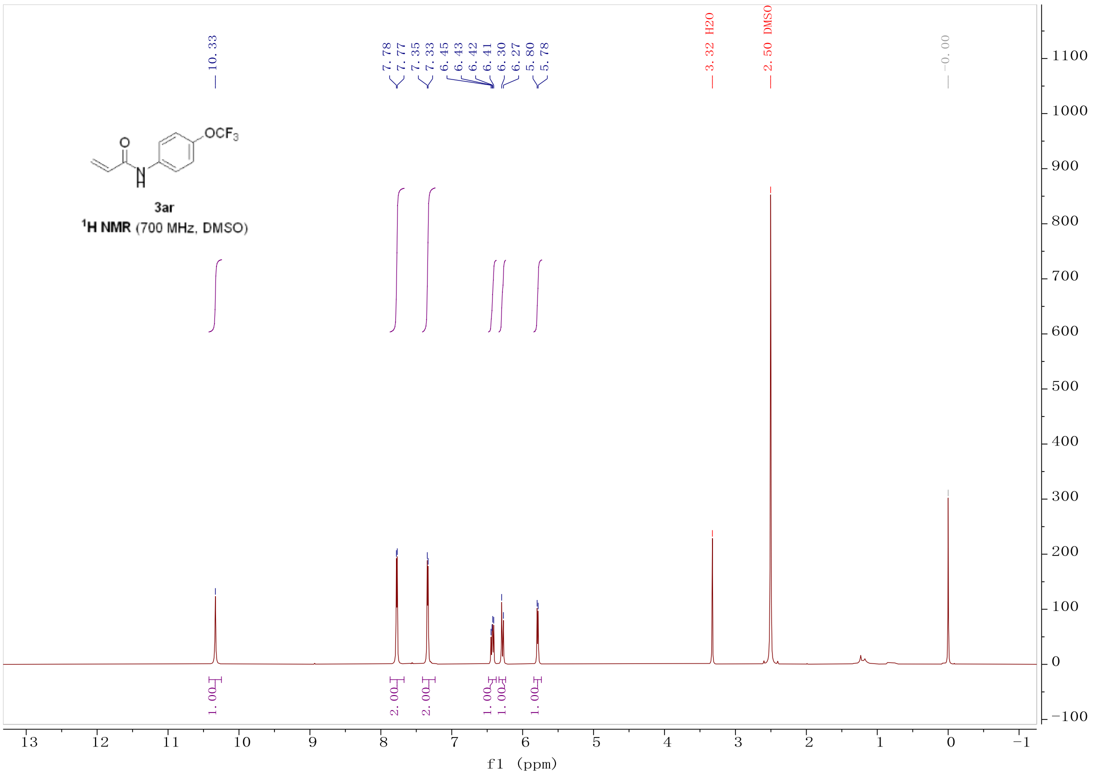
10.33
7.78
7.77
7.35
7.33
6.45
6.43
6.42
6.41
6.30
6.27
5.80
5.78
3.32 H2O
2.50 DMSO
0.00
OCF3
3ar
1H NMR (700 MHz, DMSO)
1.00
2.00
2.00
1.00
1.00
1.00
f1 (ppm)

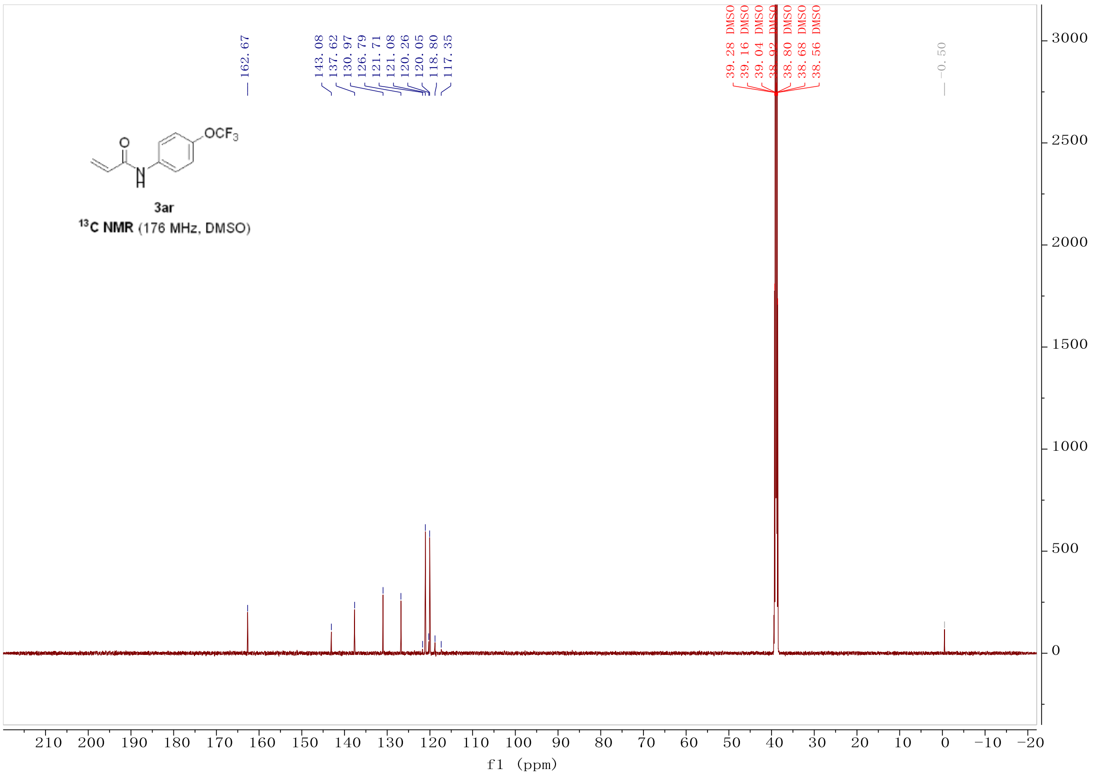
162.67
143.08
137.62
130.97
126.79
121.71
121.08
120.26
120.05
118.80
117.35
39.28 DMSO
39.16 DMSO
39.04 DMSO
38.80 DMSO
38.68 DMSO
38.56 DMSO
-0.50
OCF3
3ar
13C NMR (176 MHz, DMSO)
f1 (ppm)

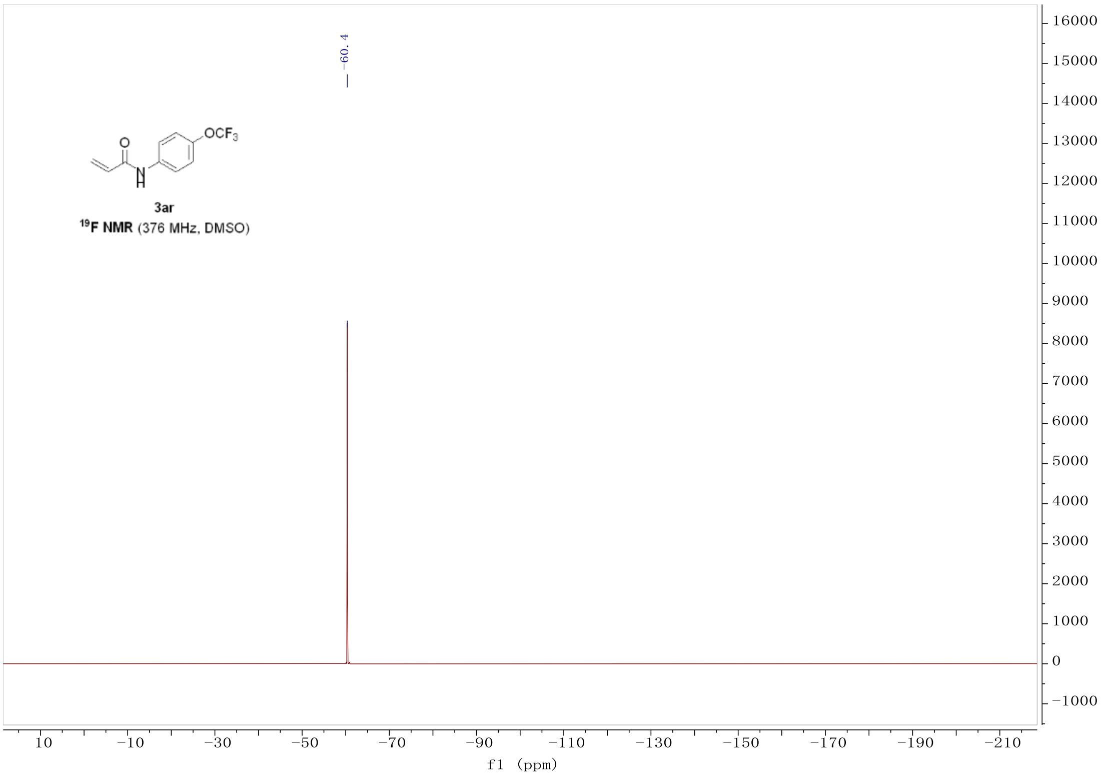

−60.4
OCF3
O
N
H
3ar
19F NMR (376 MHz, DMSO)
16000
15000
14000
13000
12000
11000
10000
9000
8000
7000
6000
5000
4000
3000
2000
1000
0
−1000
10
−10
−30
−50
−70
−90
−110
−130
−150
−170
−190
−210
f1 (ppm)

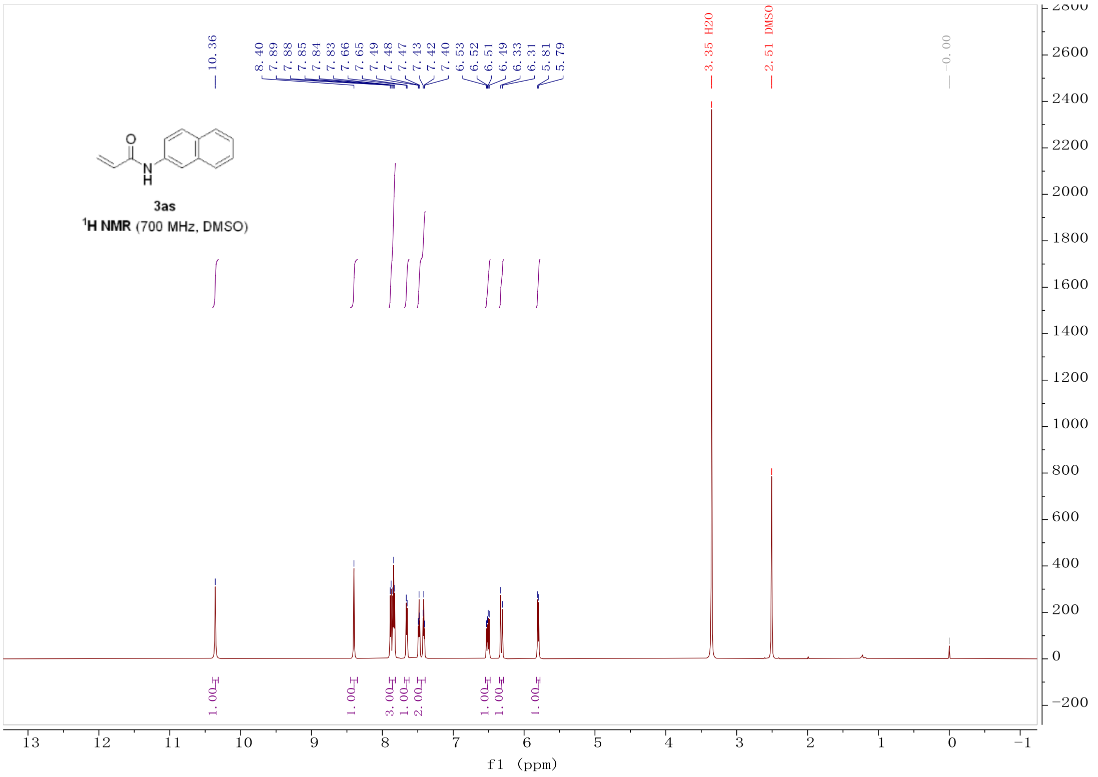

3as
1H NMR (700 MHz, DMSO)
10.36
8.40 7.89 7.88 7.85 7.84 7.83 7.66 7.65 7.49 7.48 7.47 7.43 7.42 7.40 6.53 6.52 6.51 6.49 6.33 6.31 5.81 5.79
3.35 H2O
2.51 DMSO
-0.00
1.00 1.00 3.00 1.00 2.00 1.00 1.00 1.00
f1 (ppm)

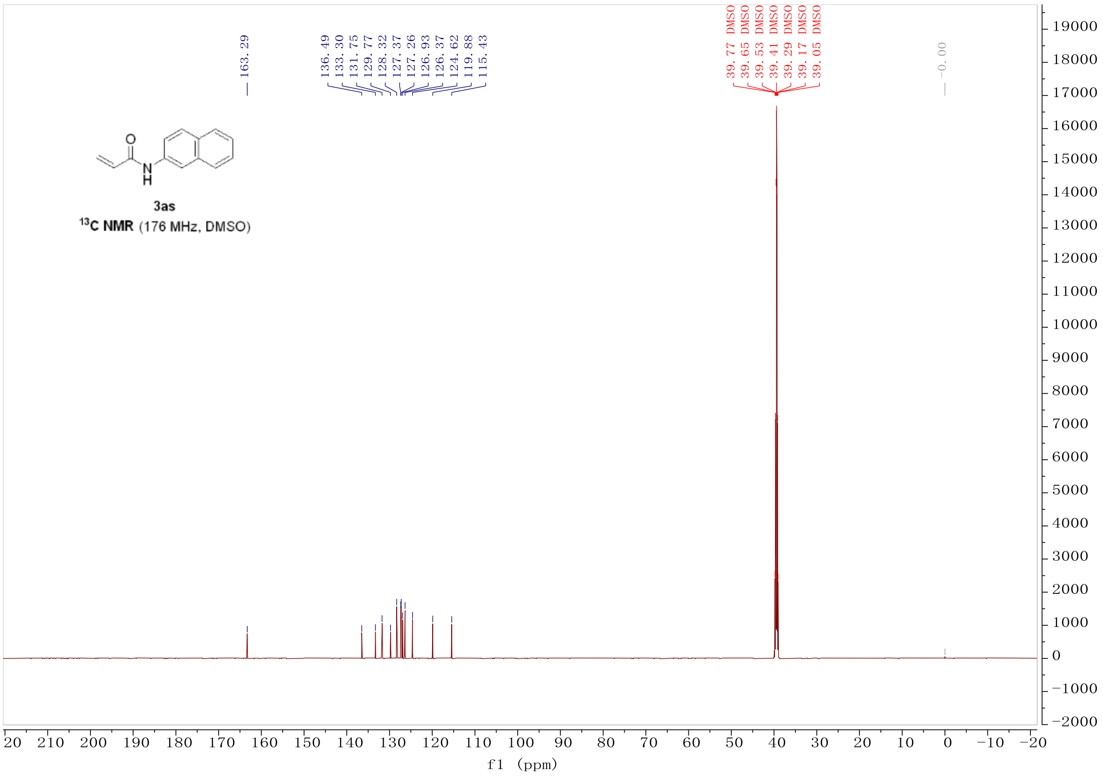

3as
13C NMR (176 MHz, DMSO)
163.29
136.49 133.30 131.75 129.77 128.32 127.37 127.26 126.93 126.37 124.62 119.88 115.43
39.77 DMSO 39.65 DMSO 39.53 DMSO 39.41 DMSO 39.29 DMSO 39.17 DMSO 39.05 DMSO
-0.00
f1 (ppm)

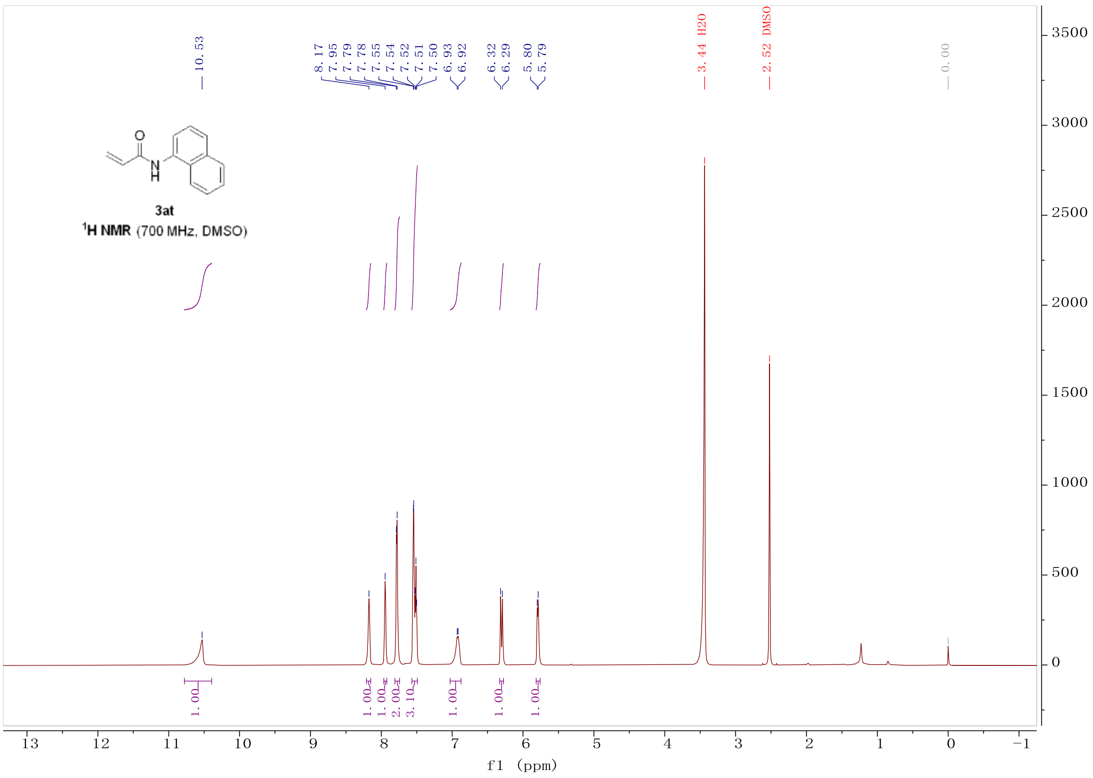
10.53
8.17
7.95
7.79
7.78
7.55
7.54
7.52
7.51
7.50
6.93
6.92
6.32
6.29
5.80
5.79
3.44 H2O
2.52 DMSO
0.00
3at
1H NMR (700 MHz, DMSO)
1.00
1.00
1.00
2.00
3.10
1.00
1.00
1.00
f1 (ppm)

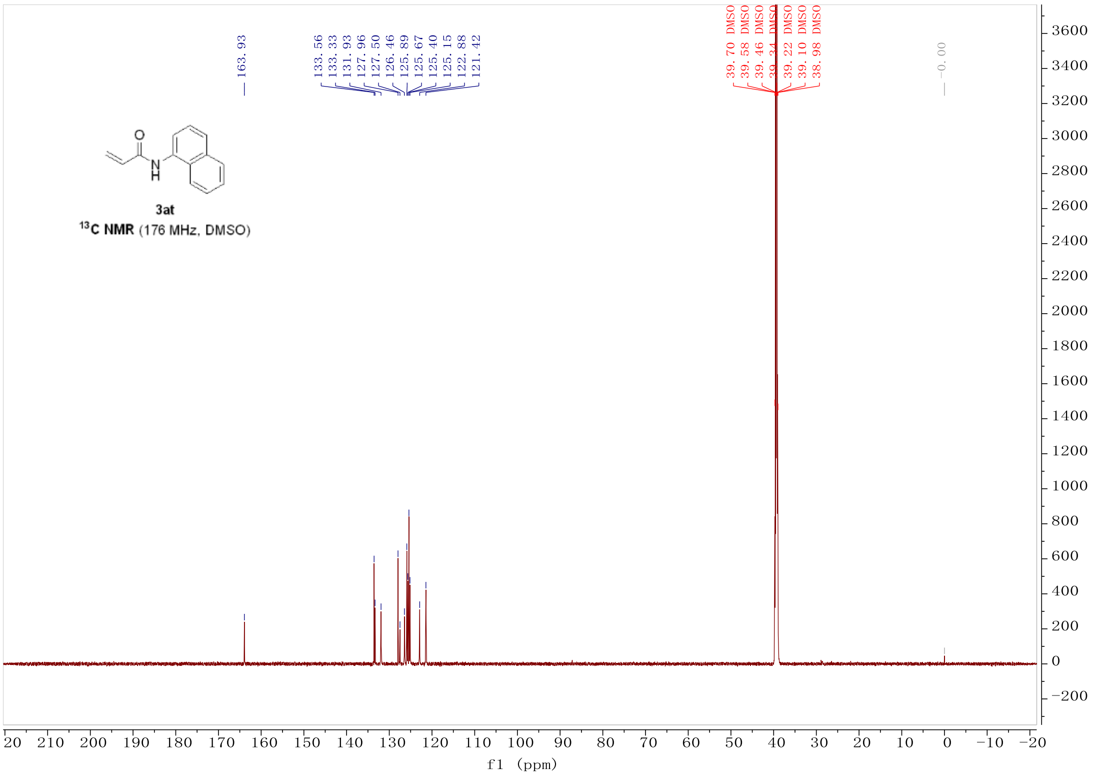
163.93
133.56
133.33
131.93
127.96
127.50
126.46
125.89
125.67
125.40
125.15
122.88
121.42
39.70 DMSO
39.58 DMSO
39.46 DMSO
39.22 DMSO
39.10 DMSO
38.98 DMSO
-0.00
3at
13C NMR (176 MHz, DMSO)
f1 (ppm)

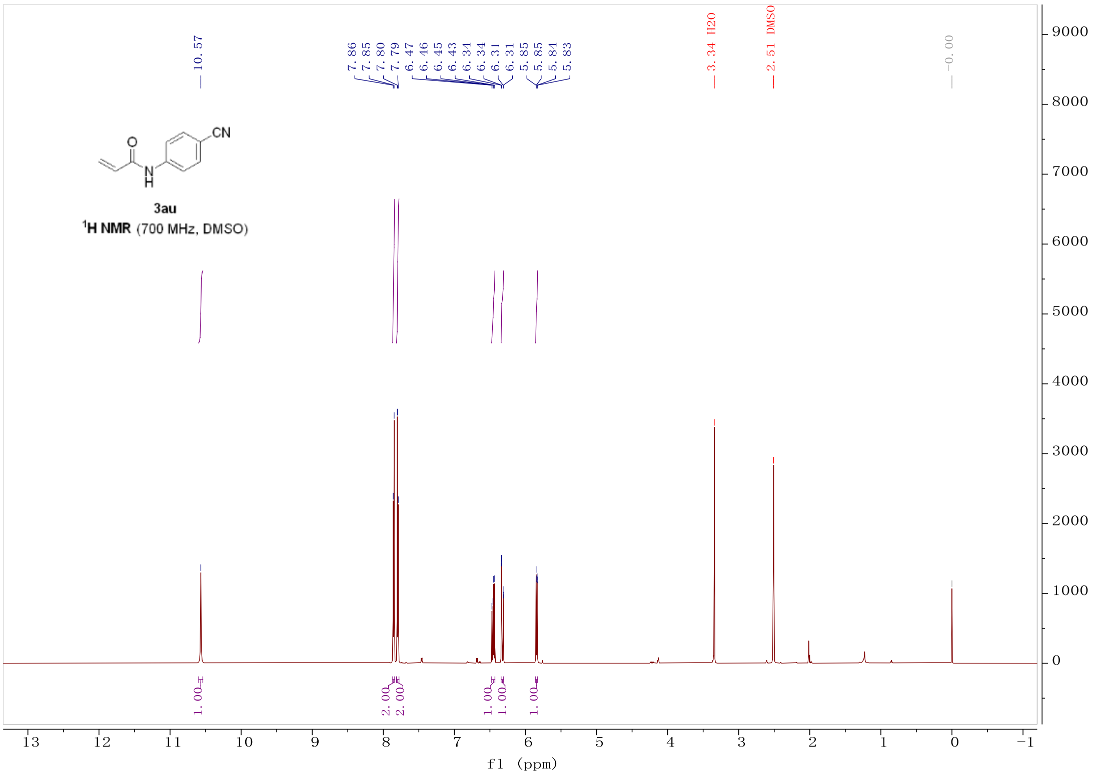
10.57
7.86
7.85
7.80
7.79
6.47
6.46
6.45
6.43
6.34
6.34
6.31
6.31
5.85
5.85
5.84
5.83
3.34 H2O
2.51 DMSO
-0.00
CN
O
N
H
3au
1H NMR (700 MHz, DMSO)
1.00
2.00
2.00
1.00
1.00
1.00
f1 (ppm)

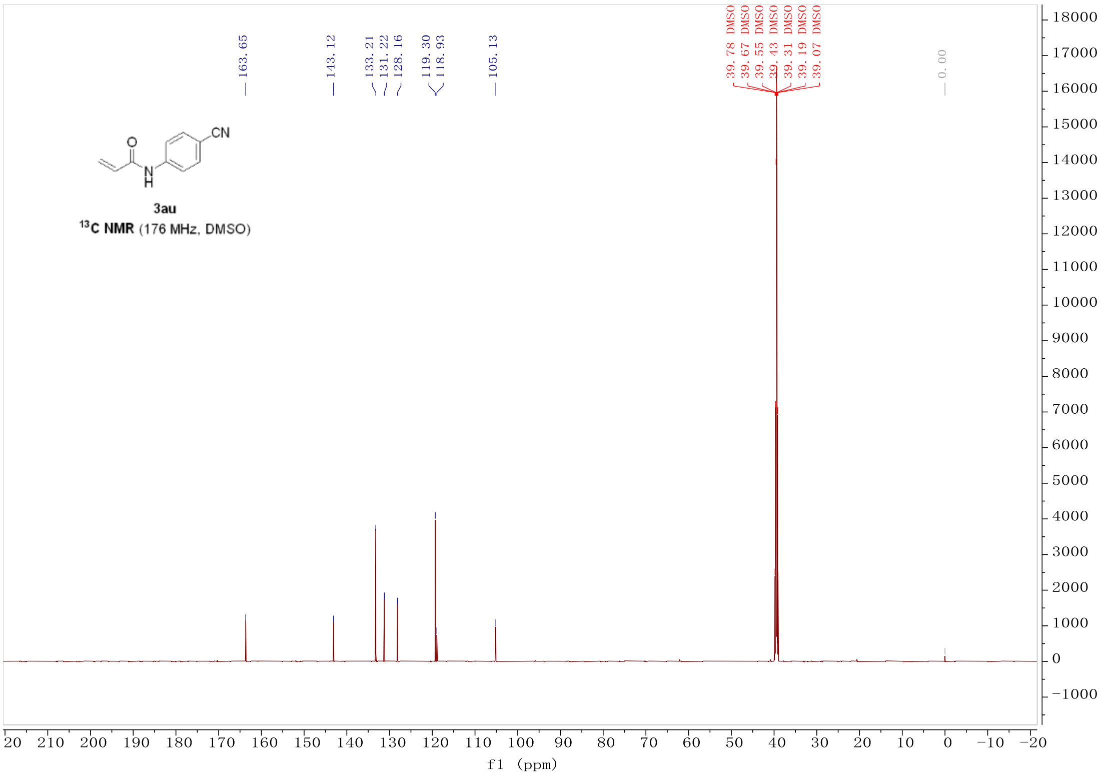
163.65
143.12
133.21
131.22
128.16
119.30
118.93
105.13
39.78 DMSO
39.67 DMSO
39.55 DMSO
39.43 DMSO
39.31 DMSO
39.19 DMSO
39.07 DMSO
0.00
CN
O
N
H
3au
13C NMR (176 MHz, DMSO)
f1 (ppm)

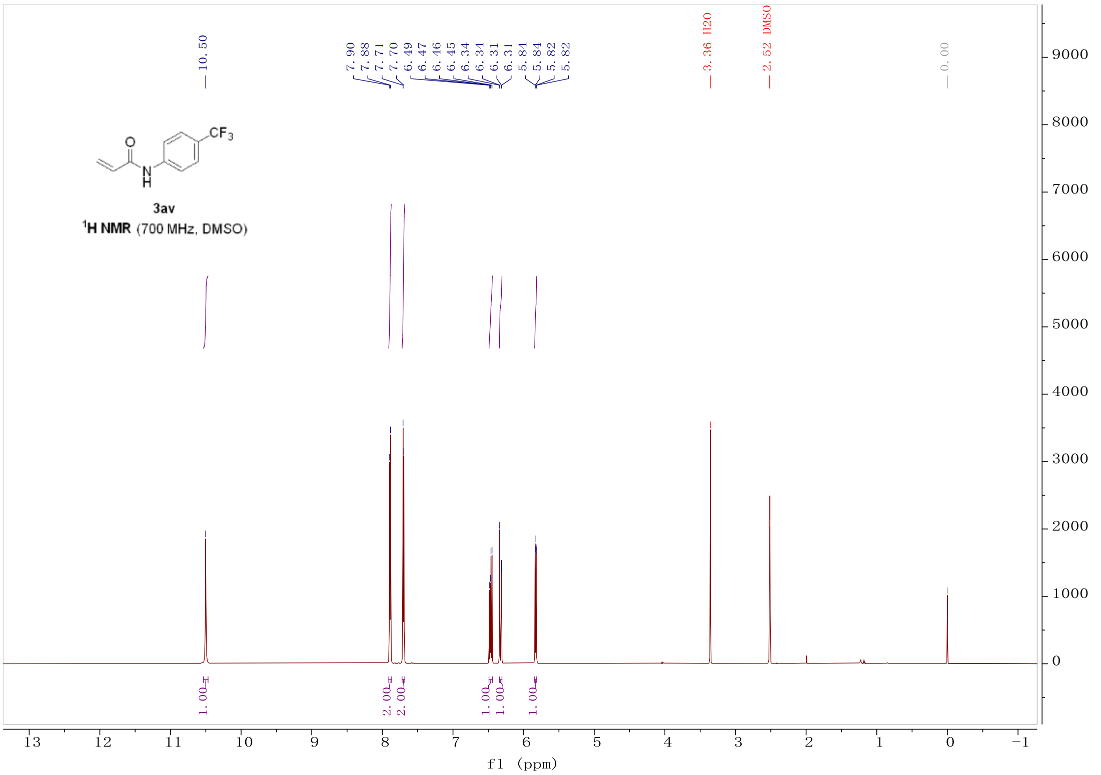
CF3
3av
1H NMR (700 MHz, DMSO)
f1 (ppm)

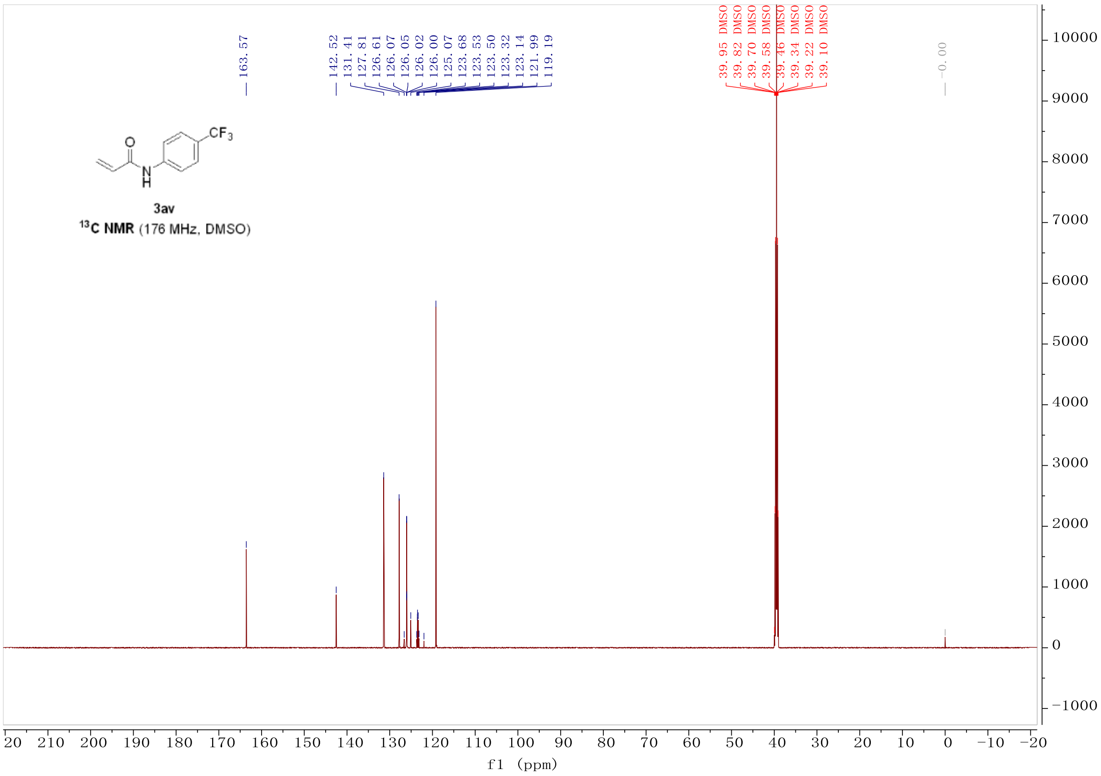
CF3
3av
13C NMR (176 MHz, DMSO)
f1 (ppm)

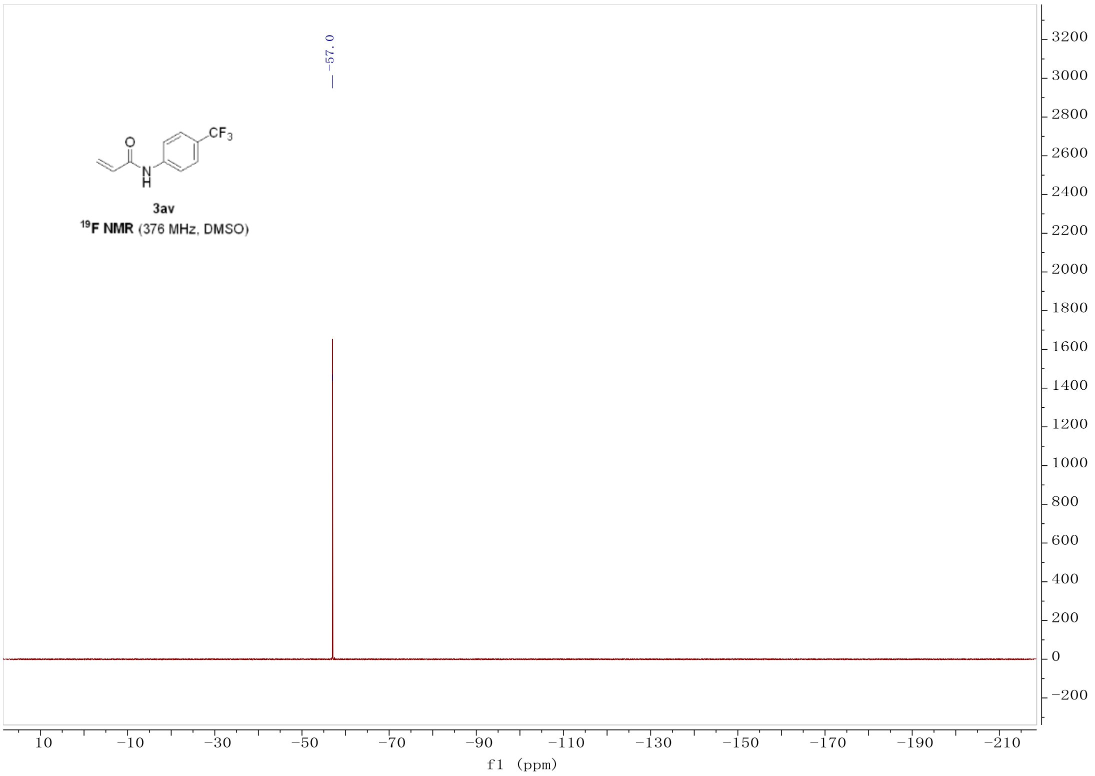

−57.0
CF3
O
N
H
3av
19F NMR (376 MHz, DMSO)
3200
3000
2800
2600
2400
2200
2000
1800
1600
1400
1200
1000
800
600
400
200
0
−200
10
−10
−30
−50
−70
−90
−110
−130
−150
−170
−190
−210
f1 (ppm)

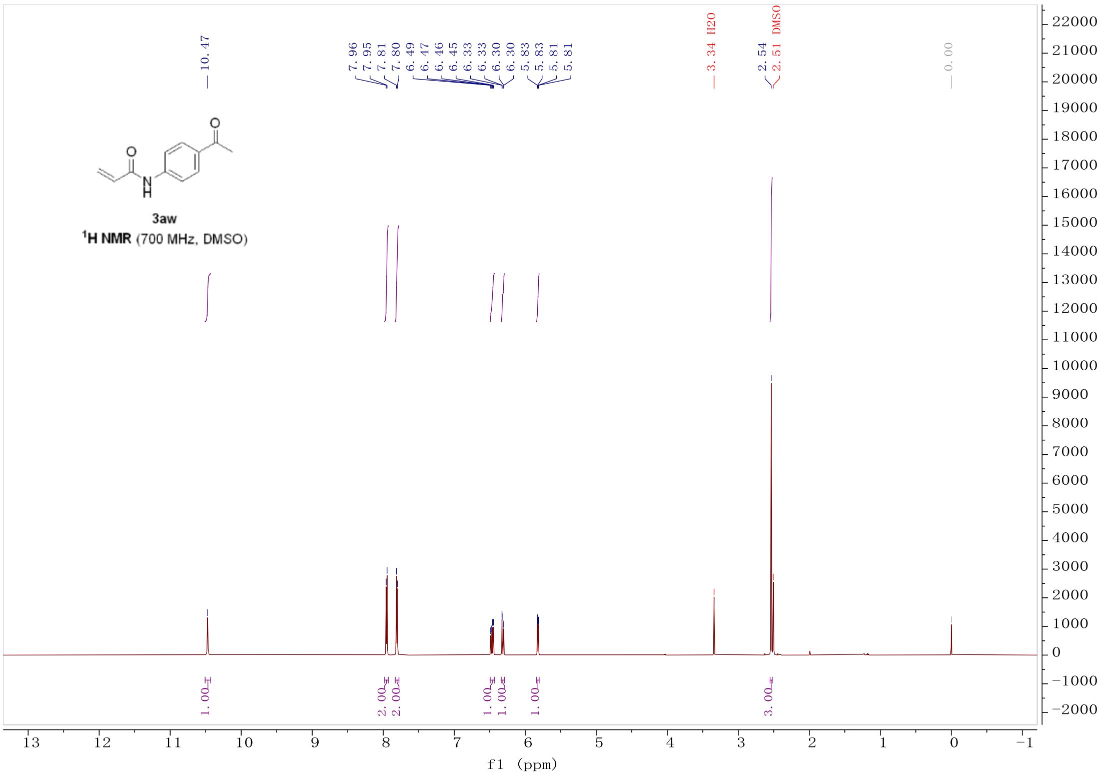
10.47
7.96
7.95
7.81
7.80
6.49
6.47
6.46
6.45
6.33
6.33
6.30
6.30
5.83
5.83
5.81
5.81
3.34 H2O
2.54
2.51 DMSO
0.00
3aw
^{1}H NMR (700 MHz, DMSO)
1.00
2.00
2.00
1.00
1.00
1.00
3.00
f1 (ppm)

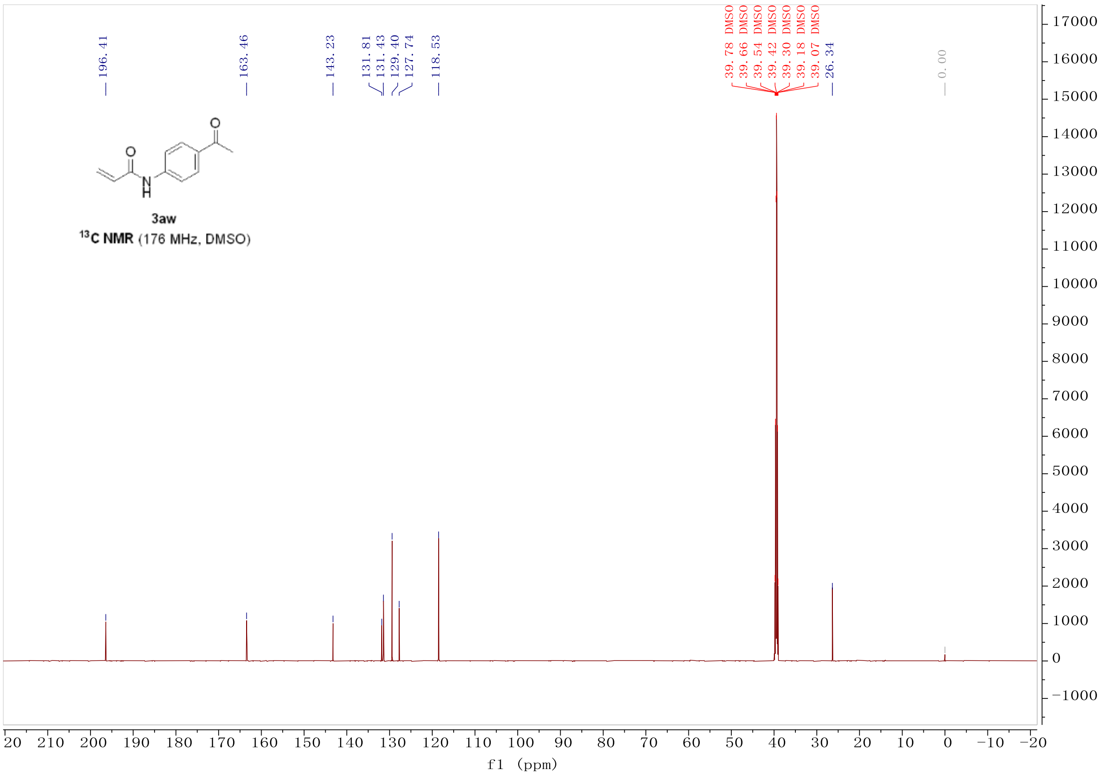
196.41
163.46
143.23
131.81
131.43
129.40
127.74
118.53
39.78 DMSO
39.66 DMSO
39.54 DMSO
39.42 DMSO
39.30 DMSO
39.18 DMSO
39.07 DMSO
26.34
0.00
3aw
^{13}C NMR (176 MHz, DMSO)
f1 (ppm)

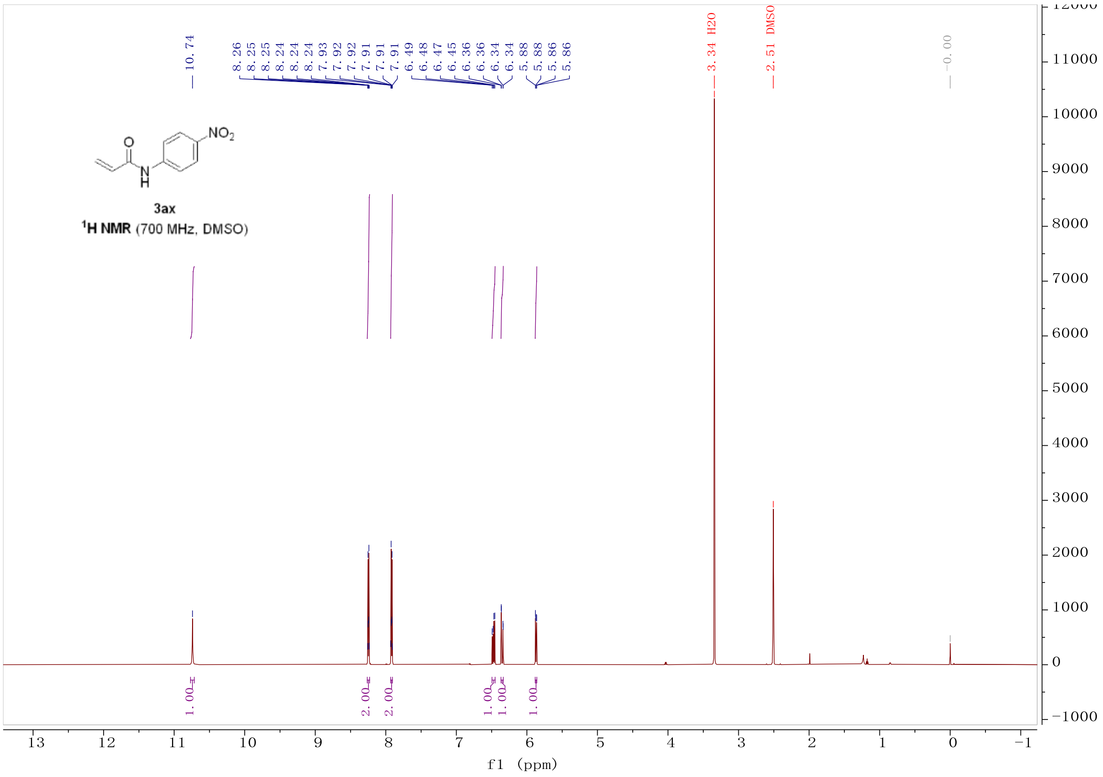
10.74
8.26
8.25
8.25
8.24
8.24
8.24
7.93
7.92
7.92
7.91
7.91
7.91
6.49
6.48
6.47
6.45
6.36
6.36
6.34
6.34
5.88
5.88
5.86
5.86
3.34 H2O
2.51 DMSO
-0.00
3ax
1H NMR (700 MHz, DMSO)
1.00
2.00
2.00
1.00
1.00
1.00
f1 (ppm)

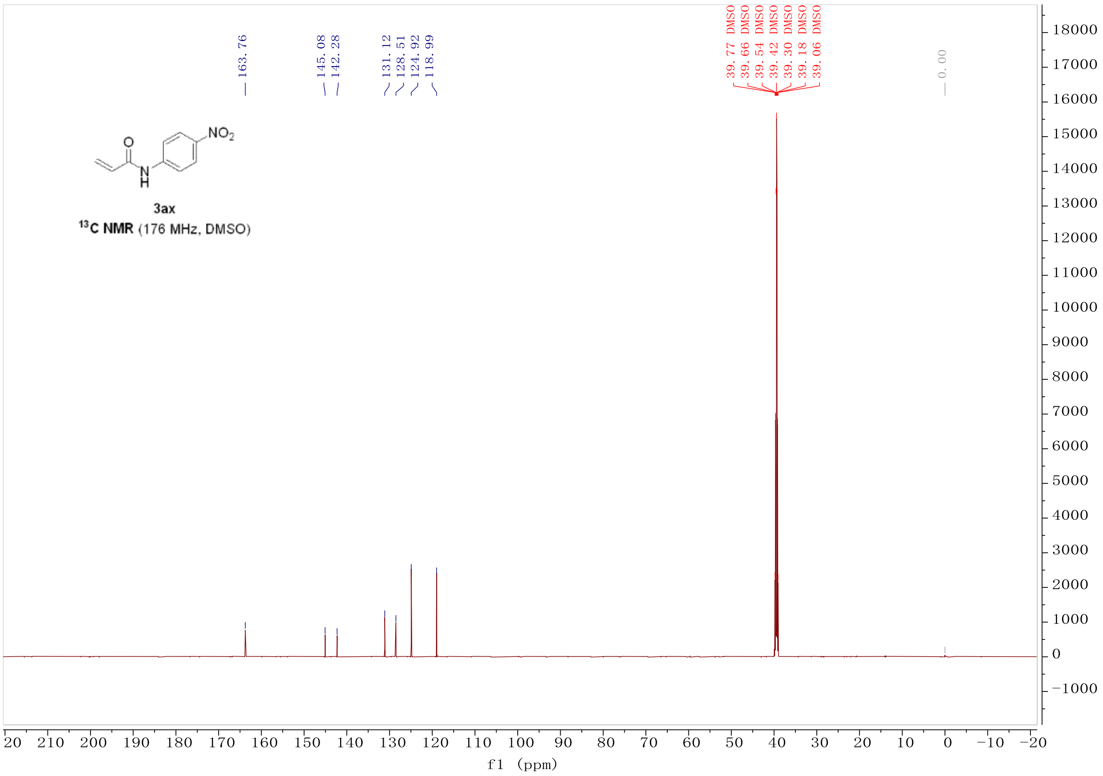
163.76
145.08
142.28
131.12
128.51
124.92
118.99
39.77 DMSO
39.66 DMSO
39.54 DMSO
39.42 DMSO
39.30 DMSO
39.18 DMSO
39.06 DMSO
0.00
3ax
13C NMR (176 MHz, DMSO)
f1 (ppm)

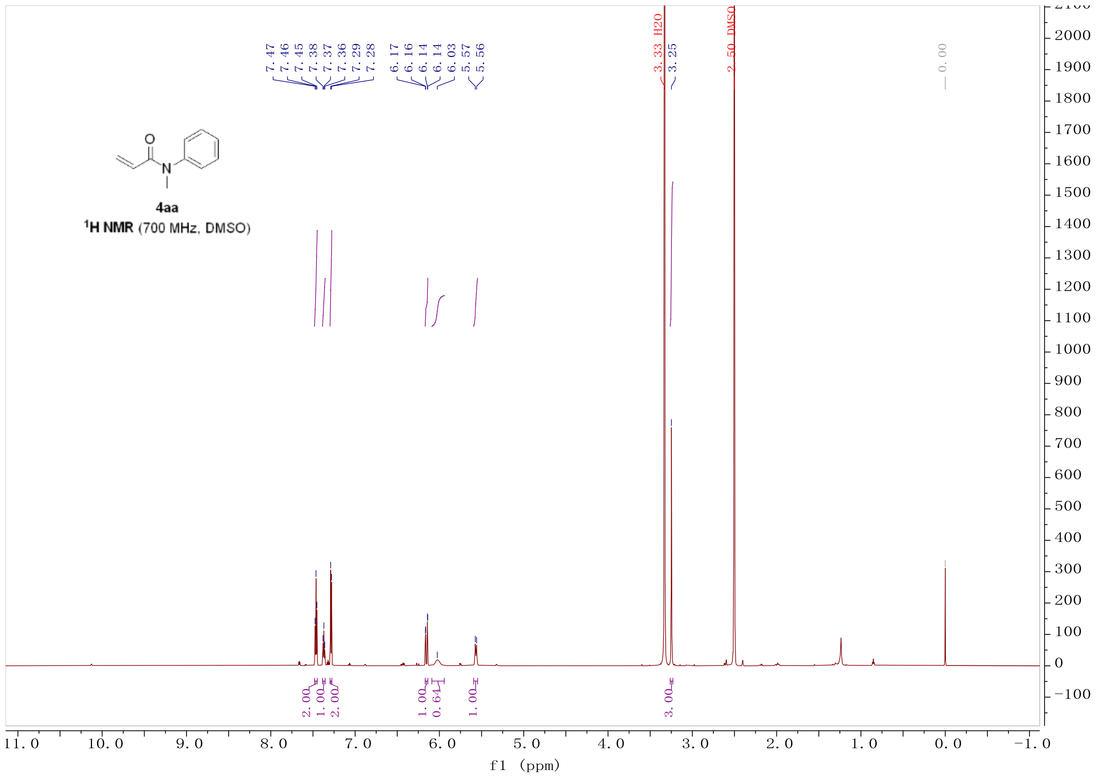
7.47
7.46
7.45
7.38
7.37
7.36
7.29
7.28
6.17
6.16
6.14
6.14
6.03
5.57
5.56
3.33 H2O
3.25
2.50 DMSO
0.00
O
N
4aa
1H NMR (700 MHz, DMSO)
2.00
1.00
2.00
1.00
0.64
1.00
3.00
f1 (ppm)

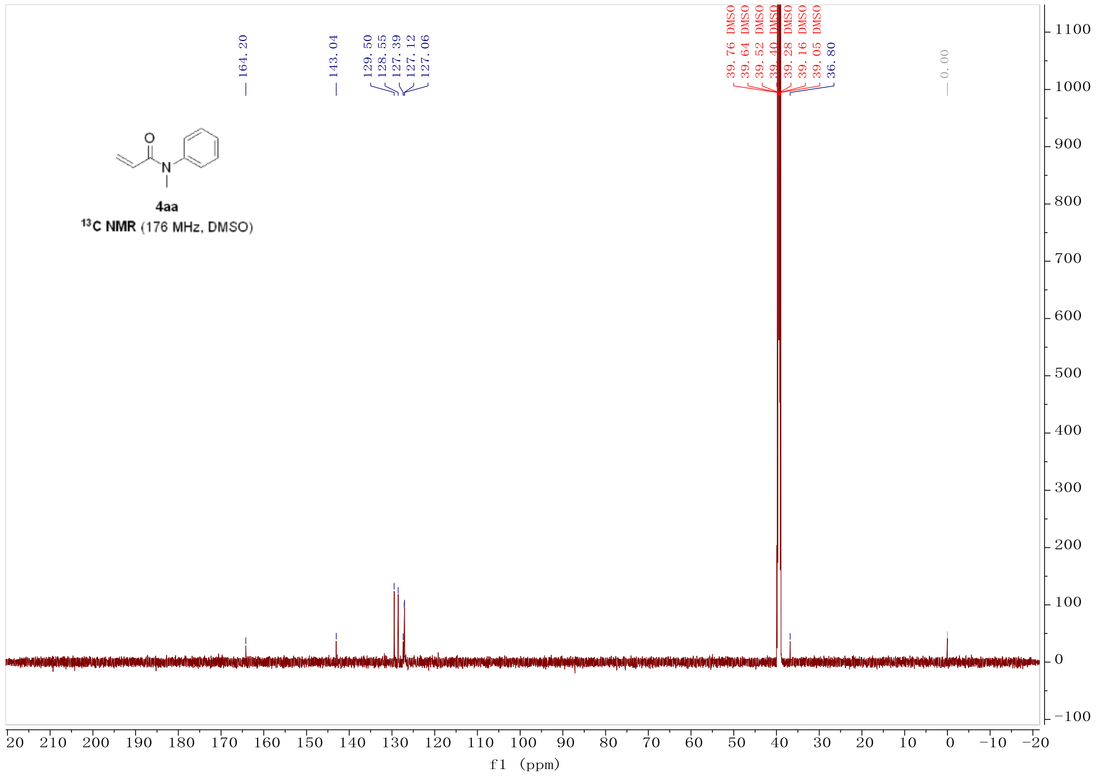
164.20
143.04
129.50
128.55
127.39
127.12
127.06
39.76 DMSO
39.64 DMSO
39.52 DMSO
39.40 DMSO
39.28 DMSO
39.16 DMSO
39.05 DMSO
36.80
0.00
O
N
4aa
13C NMR (176 MHz, DMSO)
f1 (ppm)

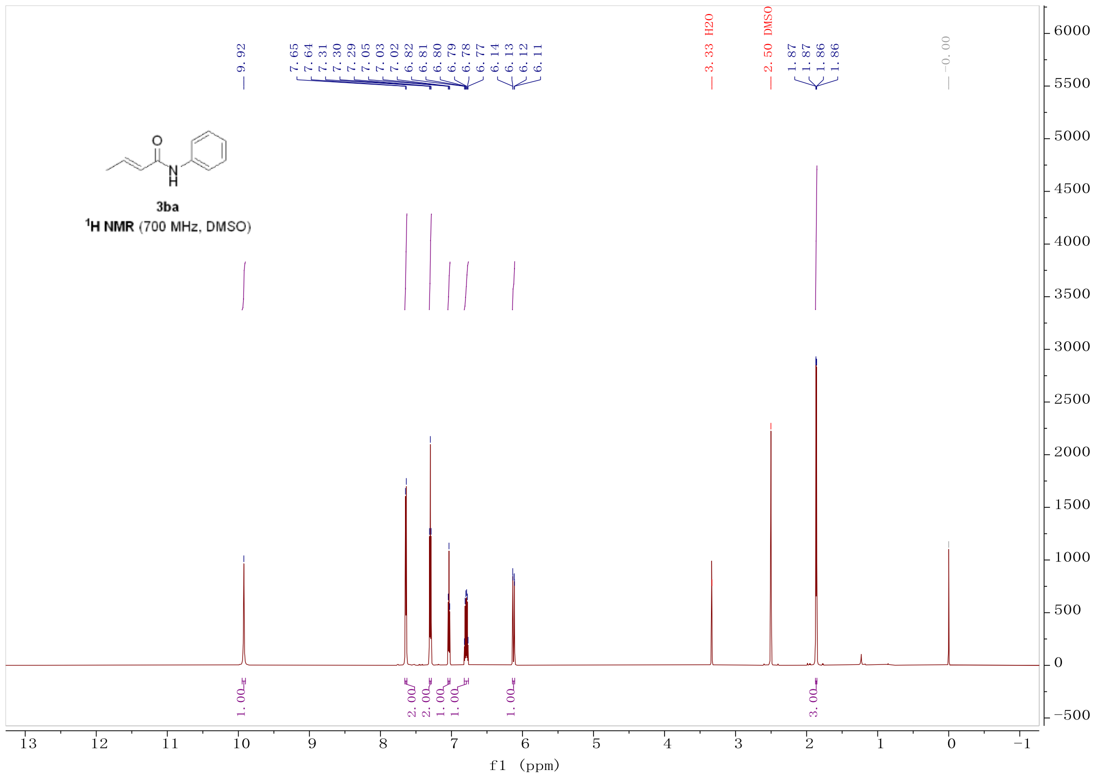
3ba
1H NMR (700 MHz, DMSO)
f1 (ppm)

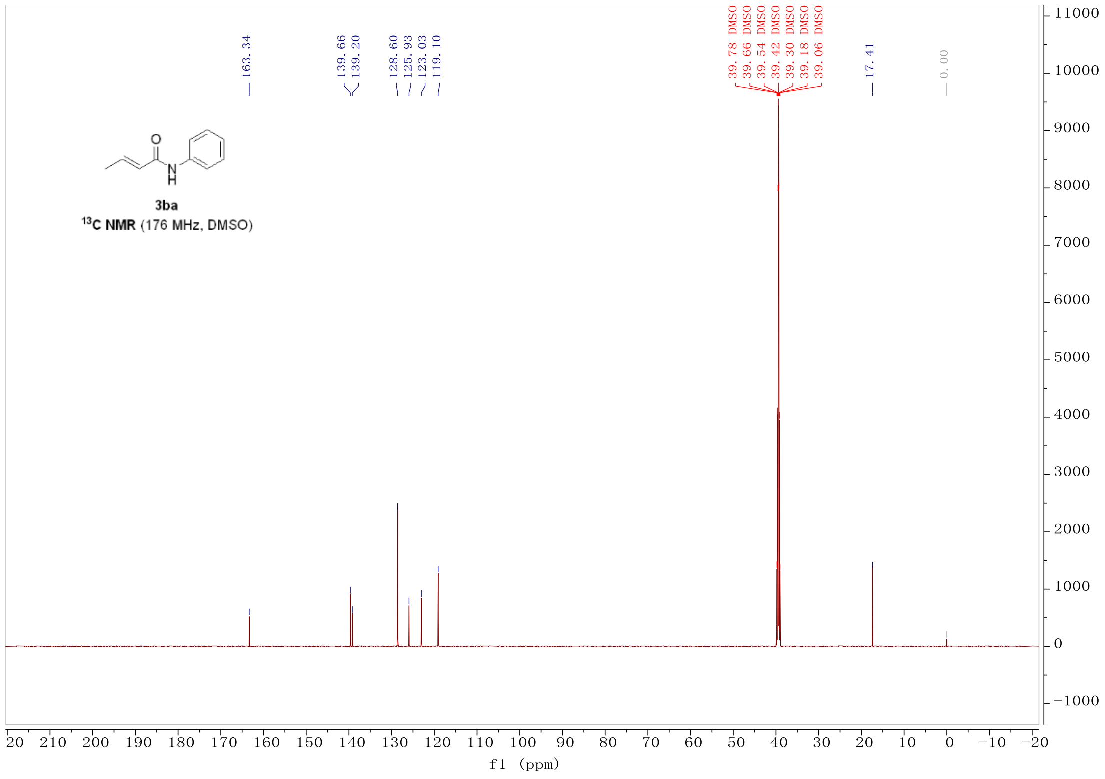
3ba
13C NMR (176 MHz, DMSO)
f1 (ppm)

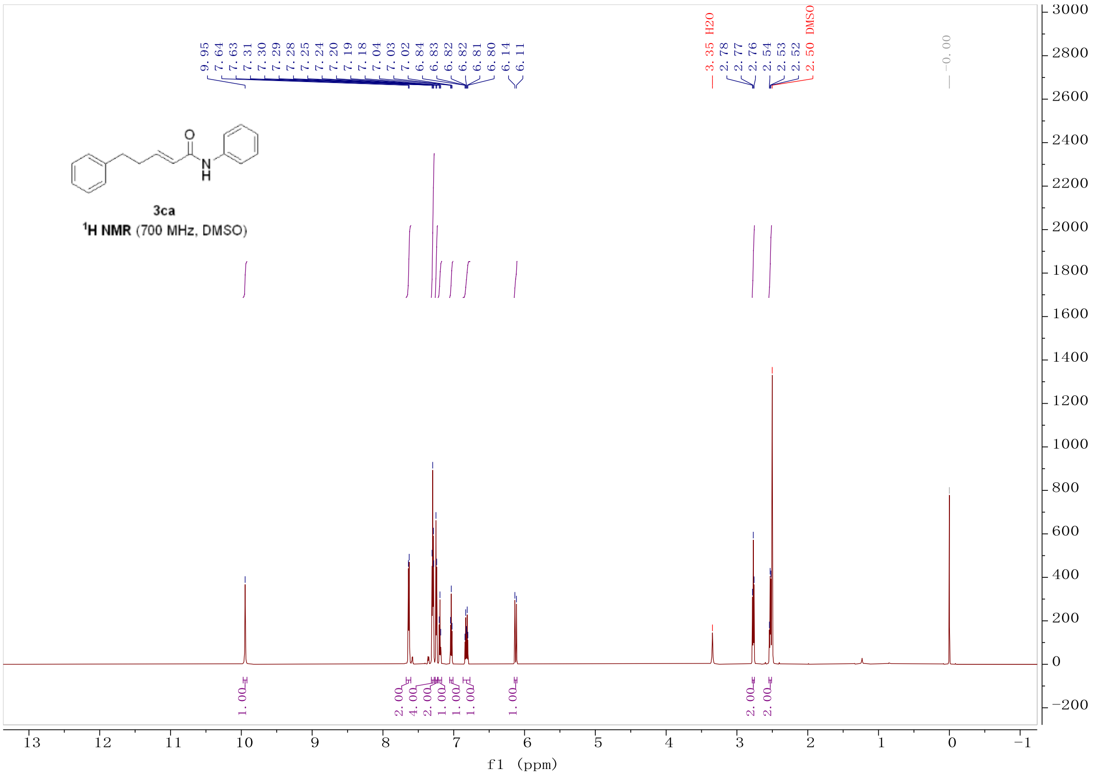
3ca
1H NMR (700 MHz, DMSO)
9.95 7.64 7.63 7.31 7.30 7.29 7.28 7.25 7.24 7.20 7.19 7.18 7.04 7.03 7.02 6.84 6.83 6.82 6.82 6.81 6.80 6.14 6.11
3.35 H2O
2.78 2.77 2.76 2.54 2.53 2.52
2.50 DMSO
-0.00
1.00 2.00 4.00 2.00 1.00 1.00 1.00 1.00 2.00 2.00
f1 (ppm)

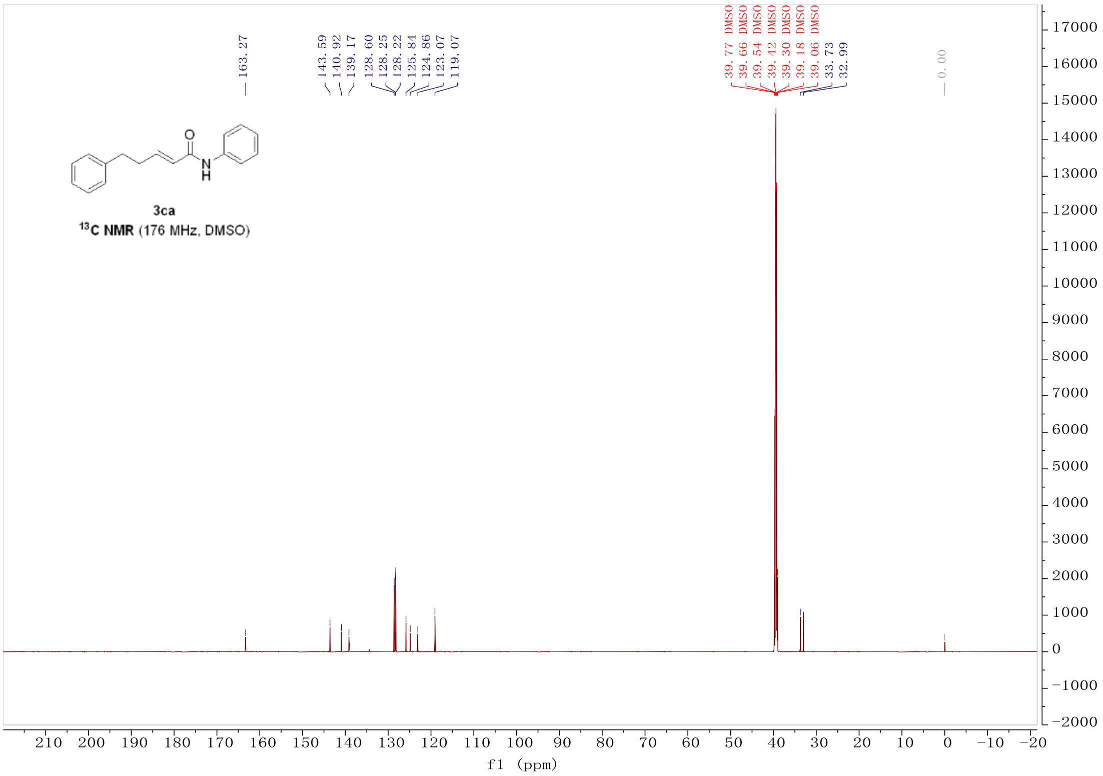
3ca
13C NMR (176 MHz, DMSO)
163.27
143.59 140.92 139.17 128.60 128.25 128.22 125.84 124.86 123.07 119.07
39.77 DMSO 39.66 DMSO 39.54 DMSO 39.42 DMSO 39.30 DMSO 39.18 DMSO 39.06 DMSO
33.73 32.99
0.00
f1 (ppm)

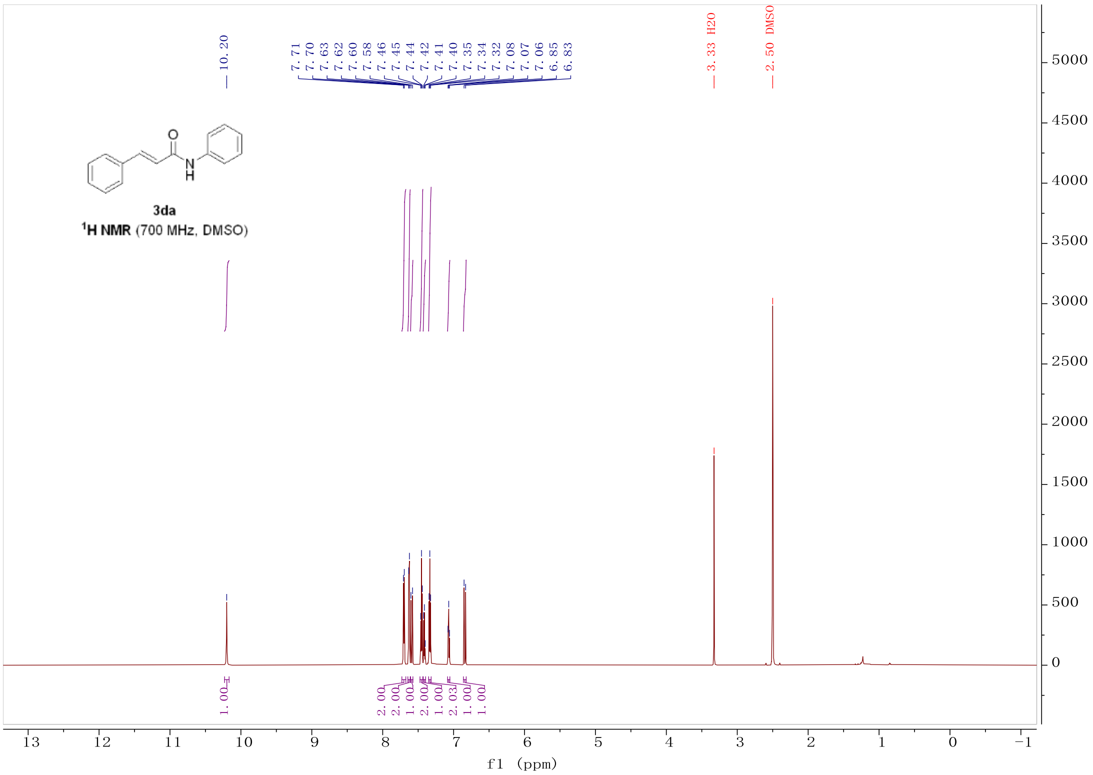
3da
1H NMR (700 MHz, DMSO)
10.20
7.71 7.70 7.63 7.62 7.60 7.58 7.46 7.45 7.44 7.42 7.41 7.40 7.35 7.34 7.32 7.08 7.07 7.06 6.85 6.83
3.33 H2O
2.50 DMSO
1.00
2.00 2.00 1.00 2.00 1.00 2.03 1.00 1.00
f1 (ppm)

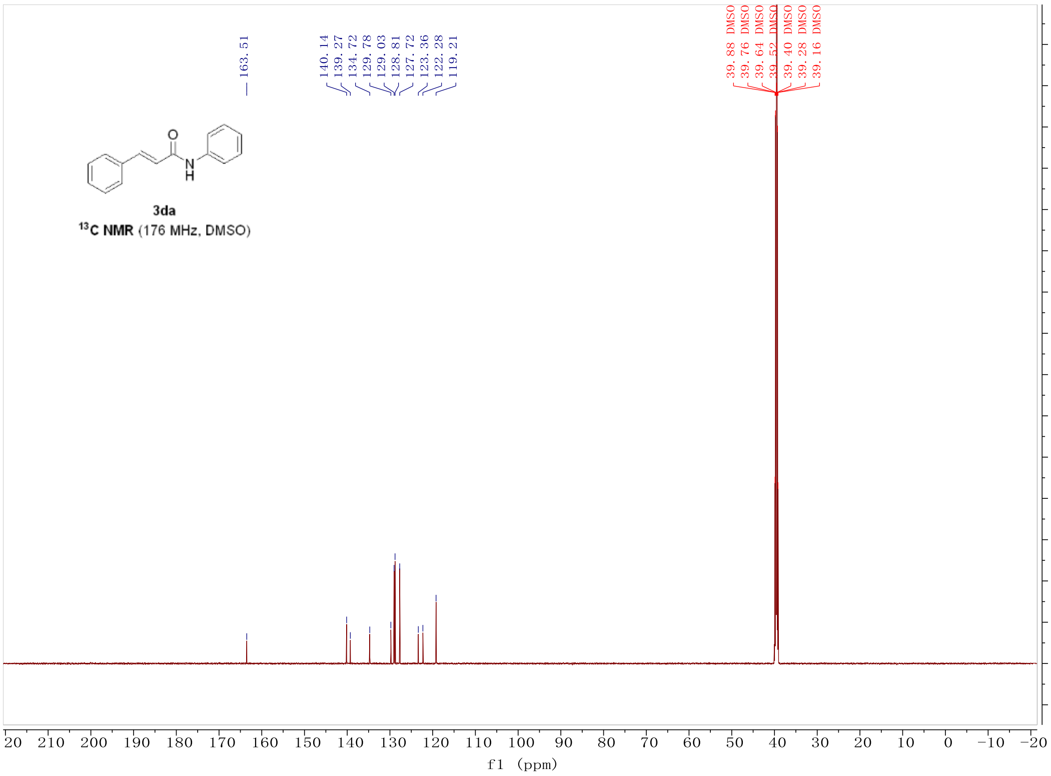
3da
13C NMR (176 MHz, DMSO)
163.51
140.14 139.27 134.72 129.78 129.03 128.81 127.72 123.36 122.28 119.21
39.88 DMSO 39.76 DMSO 39.64 DMSO 39.52 DMSO 39.40 DMSO 39.28 DMSO 39.16 DMSO
f1 (ppm)
